# Pyrosequencing of Antibiotic-Contaminated River Sediments Reveals High Levels of Resistance and Gene Transfer Elements

**DOI:** 10.1371/journal.pone.0017038

**Published:** 2011-02-16

**Authors:** Erik Kristiansson, Jerker Fick, Anders Janzon, Roman Grabic, Carolin Rutgersson, Birgitta Weijdegård, Hanna Söderström, D. G. Joakim Larsson

**Affiliations:** 1 Department of Neuroscience and Physiology, the Sahlgrenska Academy at the University of Gothenburg, Göteborg, Sweden; 2 Department of Mathematical Statistics, Chalmers University of Technology, Göteborg, Sweden; 3 Department of Chemistry, Umeå University, Umeå, Sweden; Universidad Miguel Hernandez, Spain

## Abstract

The high and sometimes inappropriate use of antibiotics has accelerated the development of antibiotic resistance, creating a major challenge for the sustainable treatment of infections world-wide. Bacterial communities often respond to antibiotic selection pressure by acquiring resistance genes, i.e. mobile genetic elements that can be shared horizontally between species. Environmental microbial communities maintain diverse collections of resistance genes, which can be mobilized into pathogenic bacteria. Recently, exceptional environmental releases of antibiotics have been documented, but the effects on the promotion of resistance genes and the potential for horizontal gene transfer have yet received limited attention. In this study, we have used culture-independent shotgun metagenomics to investigate microbial communities in river sediments exposed to waste water from the production of antibiotics in India. Our analysis identified very high levels of several classes of resistance genes as well as elements for horizontal gene transfer, including integrons, transposons and plasmids. In addition, two abundant previously uncharacterized resistance plasmids were identified. The results suggest that antibiotic contamination plays a role in the promotion of resistance genes and their mobilization from environmental microbes to other species and eventually to human pathogens. The entire life-cycle of antibiotic substances, both before, under and after usage, should therefore be considered to fully evaluate their role in the promotion of resistance.

## Introduction

The use, overuse and sometimes misuse of antibiotics in human and veterinary medicine are major promoters for the development and spread of multiresistant bacteria world-wide [1], [2]. The large usage of antibiotics also results in environmental releases [3], [4], [5]. Even though the levels found in the environment are low, in general below the minimum inhibitory concentration for most bacteria, they may still result in an increased selection pressure at some sites [4], [6]. Indeed, high levels of antibiotic resistance genes have been identified in a variety of milieus, particularly those exposed to antibiotics used in animal husbandry, including soils fertilized with manure and river waters contaminated by runoff from farms [5], [7], [8]. An environmental issue of potentially even higher concern is the releases of extraordinary concentrations of antibiotic substances from drug manufacturing sites. Recently, oxytetracycline was detected at up to 20 mg/L in the treated effluent from a drug production unit in China [9], [10]. From another treatment plant located in India, the final effluent contained up to 31 mg/L of broad-spectrum fluoroquinolone antibiotics, including ciprofloxacin [11], leading to unprecedented contamination of surface, ground and drinking water [12]. The levels of ciprofloxacin in surface water around the Indian plant exceeded human therapeutic blood plasma concentrations, and were thus presumably toxic for a wide range of bacteria. Due to the risk of development and rapid global transmission of resistant pathogens, the ultimate consequences of high environmental releases of antibiotics need to be thoroughly addressed.

Since antibiotics are also naturally produced by organisms such as fungi, actinomycetes and other bacteria, they are ubiquitously and perpetually present in ecosystems. Bacteria have consequently evolved a plethora of different resistance genes [6] of which many are mobile and can easily spread between species including human pathogens [13], [14]. Recent studies have demonstrated a surprisingly diverse collection of resistance genes maintained by environmental bacterial communities [15], [16], [17], [18], [19], [20]. This pool of genetic material, named the resistome, provides the molecular functions for protecting bacteria against most classes of clinically important antibiotics. The environmental resistome therefore constitutes a reservoir of resistance genes that can be mobilized into human pathogenic bacteria [13], [21].

The vast majority of all environmental bacteria cannot be grown in the laboratory using standard protocols (<1% are estimated to be cultivable, [22]). Assessment of antibiotic resistance in environmental microbial communities based solely on cultivable bacteria will therefore easily generate unrepresentative and biased results [20], [23] [23]. Furthermore, the large number of reported resistance genes renders targeted molecular approaches, such as PCR, inefficient. In contrast, shotgun metagenomics, where all DNA from a sample is extracted and sequenced, provides a culture-independent and explorative alternative to characterize the genetic basis for resistance within microbial communities.

The present study constitutes the first culture-independent metagenomic analysis of environmental bacterial communities polluted with high levels of antibiotics. Using high-throughput sequencing of DNA from river sediment up and downstream from the Indian treatment plant [11] we identified a high prevalence of resistance genes from multiple classes of antibiotics. We also found a high abundance of elements for horizontal transfer of resistance genes, including integrons, transposons and plasmids. The results suggest that environmental releases of effluent contaminated with antibiotics promote resistance genes and genetic elements for their mobility.

## Results and Discussion

**Figure 1:**
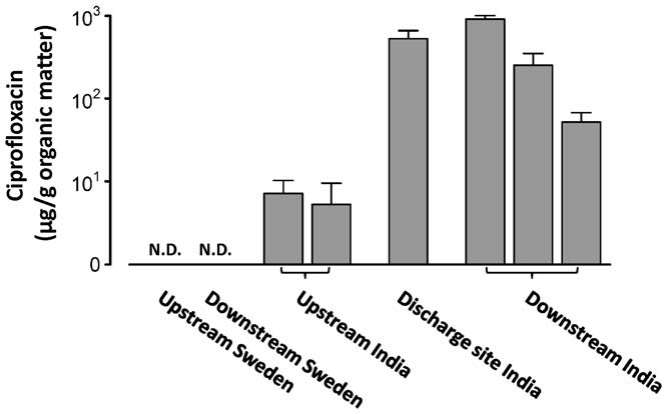
High levels of ciprofloxacin were found in river sediments downstream from the Indian treatment plant. The figure shows the amount of ciprofloxacin in relation to the total organic mass (µ*g/g*) measured by liquid chromatography mass spectrometry. The levels were considerable even at the most distant sampling site (located 17.5 km downstream from the discharge site). No detectable levels were found at the Swedish treatment plant (marked with N.D.). The error bars describe the standard error of the mean based on the analysis of three separate samples taken in close vicinity.

River sediment samples were collected in a gradient up and downstream from an Indian waste water treatment plant processing effluent from more than 90 bulk drug manufacturers producing a wide range of antibiotics and other pharmaceuticals [11]. High levels of several broad spectrum fluoroquinolone antibiotics were previously documented in effluent and surface water, ciprofloxacin being the most abundant [11], [12]. Here we show that the river sediments are also contaminated by high levels of ciprofloxacin downstream from the treatment plant (up to 914 µ*g/g* organic matter), and moderately elevated levels were found in upstream sediment (up to 7.1 µ*g/g* organic matter) (Figure 1, Tables S1, S2 and S3). As a control, additional samples were therefore collected up and downstream from a Swedish sewage effluent treatment plant not connected to any production of pharmaceuticals. No detectable levels of fluoroquinolones could be found in the Swedish sediment samples (Table S2).

The sediment DNA was analyzed by multiplexed massively parallel pyrosequencing, generating 441,523 reads comprising 161 million base pairs in total (Table S2). Half of the reads (44.8%) could be annotated of which the majority was of bacterial origin (85.5%) while smaller fractions came from viruses (9.5%), eukaryotes (2.9%) and archaea (2.1%) (Figure S1 and Table S4). Rarefaction curve analysis revealed that the bacterial diversity downstream from the Indian treatment plant was slightly lower than upstream, but the difference was surprisingly small considering the high levels of antibiotics present (Figure S2 and S3). All communities were dominated by *Proteobacteria*, *Bacteroidetes* and *Firmicutes* (Table S5 and Figure S4), but the distributions of bacterial genera were significantly different (p<10^−16^) and the up and downstream communities clustered separately (Figure S5). Functional analysis showed significant differences between the metagenomes and the downstream bacterial communities contained a high abundance of genes associated with antibiotic resistance (COG0294, 3570, 3231, 1357), replication and mobilization of DNA (COG3668, TIGR01629, TIGR02768) and membrane transporters (COG0488, TIGR02294) (Tables S6, S7, S8, S9, S10, S11).

**Figure 2:**
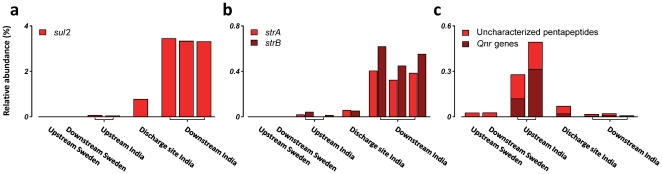
Exposure to antibiotic-contaminated effluent promotes resistance genes in bacterial communities in river sediment. The figure shows the relative abundance of **a**) *sul2*, **b**) *strA/B* and c) genes with a pentapeptide structure, including known *qnr*-genes, providing resistance against fluoroquinolones. The site in the immediate vicinity of the discharge had intermediate levels of both *sul2* and *strA/strB* which are likely to reflect the continuous transport and deposition of surface sediment from the upstream area. The relative abundance was calculated in relation to the total number of identified bacterial genes.

To characterize the resistome in detail we searched the metagenomes for signatures of known antibiotic resistance genes. We used a reference database consisting of 23,875 sequences describing 380 known resistance genes for 90 different modes of resistance and compared the relative abundance of matching genes using a Poisson linear model [24]. Significant differences in abundances were found for several resistance genes associated with resistance to several classes of antibiotics, including sulfonamides, fluoroquinolones and aminoglycosides (Figure 2 and Tables S12, S13, S14, S15, S16). The abundance was highest at the three Indian downstream sites where 2,726 (1.71%) of the reads could be matched to known resistance genes, while the numbers for the upstream sites and the Swedish up and downstream sites were 207 (0.22%), 31 (0.05%) and 6 (0.02%) respectively. The most abundant resistance gene was *sul2*, which encodes for a sulfonamide resistance variant of dihydropteroate synthase – an enzyme essential for folate synthesis [25]. The *sul2* gene was highly and consistently enriched downstream from the Indian treatment plan (66 times, FDR<10^−16^, Figure 2a). We also found high levels of *strA* and *strB*, two resistance genes encoding for the aminoglycoside phosphotransferases APH(3″)-Ib and APH(6)-Id which inactivates streptomycin by phosphorylation [26]. The levels of *strA* and *strB* detected downstream were 22 and 54 times higher than upstream levels, respectively (FDR<10^−16^), and no copies were found at the Swedish sites (Figure 2b).

Mobile resistance to quinolones is provided by pentapeptide repeat proteins (PRP) which interact with the target sites on gyrase and topoisomerase IV thereby preventing the inhibition of DNA supercoiling [27]. By searching the metagenomes for peptides with a PRP-like structure using a position-specific scoring matrix (PSSM) we found 157 reads classified as PRPs at the upstream sites (26 times higher than downstream, FDR<10^−16^) (Figure 2c). Ninety of these (57%) matched previously described classes of mobile quinolone resistance genes: *qnrD* (28.7%), *qnrS* (7.0%) and *qnrVC* (21.7%) [28], [29], [30]. Interestingly, many of the reads classified as PRPs did not have any clear match to known *qnr* genes (67 of 157, 42.8%) which may suggest the presence of bacteria with chromosomal PRPs and/or novel mobile *qnr* genes [31]. The considerably lower levels of PRPs at the downstream sites are likely a consequence of their inability to provide resistance to the high levels of fluoroquinolones present at these sites (Figure 1) [12], [32].

**Figure 3:**
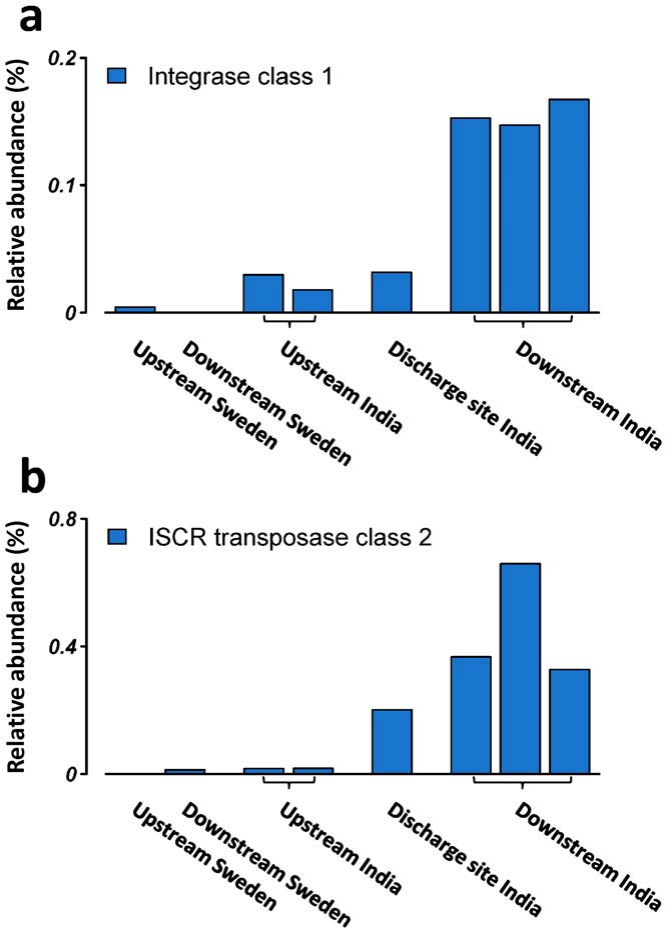
Enzymes involved in horizontal transfer of resistance genes were highly enriched in the river sediment exposed to antibiotics. The panel shows the relative abundance of **a**) integrase of class 1 and **b**) insertion sequence common region transposase of class 2. The relative abundance was calculated in relation to the total number of identified bacterial genes.

The mobility of resistance genes is dependent on integrons, transposons and plasmids [33], [34]. The relative abundance of class 1 integrases were significantly higher downstream compared to upstream from the Indian treatment plant (6.7 times higher, FDR = 1.5×10^−9^, Figure 3a), while no reads matched other forms of integrases (class 2 to 10). Integrons capture and disseminate gene-cassettes; short circular pieces of DNA that may contain resistance genes [35], and integrons associated with class 1 integrases are known to transfer many forms of resistance genes [36]. At the downstream sites, we also found 24 times higher levels of a transposase associated with insertion sequence common regions (ISCRs) (FDR<10^−16^, Figure 3b), a family of transposons that uses rolling-circle replication to mobilize larger segments of DNA [37]. The identified ISCR transposases were of class 2, which is known to mobilize several types of antibiotic resistance genes including *sul2* [38].

**Figure 4:**
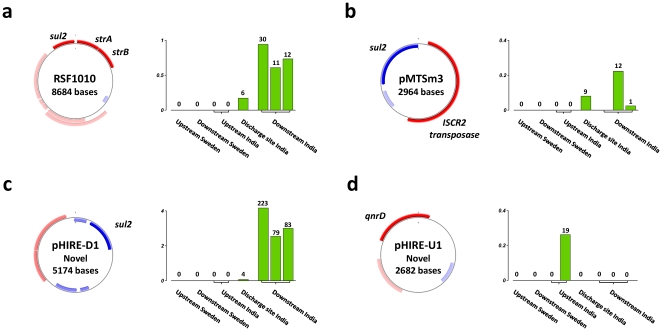
The abundance of resistance-carrying plasmids in environmental bacterial communities exposed to antibiotics. Four plasmids were detected at high levels, two already described (a–b) and two previously not characterized (c–d). Their relative abundance is given in relation to the total amount of sequenced DNA and the numbers above the bars show the estimated plasmid coverage in each metagenome.

Plasmids constitute the main vehicle for intercellular transmission of genetic material and we consequently screened the metagenomes for plasmid-associated DNA (based on 11,957 reported plasmid sequences). Two non-conjugative plasmids, RSF1010 (100% sequence similarity to NC_001740) and pMTSm3 (100% sequence similarity to AM182029), were highly abundant at downstream sites (Figure 4a–b). The RSF1010 plasmid is ubiquitous and harbors both *sul2* and *strA*/*strB* resistance genes as well as a truncated class 2 ISCR transposase [39], while pMTSm3 contains *sul2* and a full length class 2 ISCR transposase [38]. Two previously undescribed plasmids were assembled *de novo* (Figure 4c) and both were confirmed by PCR and gel-electrophoresis (Figure S6). The plasmid pHIRE-D1 (HQ540671) contained a *sul2* resistance gene in combination with a class 2 ISCR transposase in a similar structure as in pMTSm3. Two additional genes, *rep* and *mob*, showed high similarities to previously described genes for replication and mobilization of plasmids, however their exact identity could not be established (Figure 4c). The pHIRE-D1 plasmid represented 3.4% of the total sequenced DNA from the downstream sites (Figure 4c) which suggests that it may be one of the main vehicles for *sul2*. The second assembled plasmid (pHIRE-U1, HQ540672, Figure 4d) was highly abundant at the first upstream site (0.26% of the DNA). This plasmid contained the recently reported fluoroquinolone resistance gene *qnrD*, which to our best knowledge has up to now only been found in a different plasmid from *Salmonella enterica* isolated in China [28]. All of the four identified plasmids where highly abundant and together they corresponded to 0.13% and 4.1% of the DNA in the Indian up and downstream sites respectively.

Taken together, our data shows that exposure to effluent contaminated with antibiotics promote resistance genes in environmental bacterial communities. The chemical analysis revealed extraordinary levels of fluoroquinolones in effluent, river water and sediment (Figure 1) [11], [12]. Our finding of high levels of *sul2* led us to investigate the presence of sulfonamides. However, none of the 42 sulfonamides, sulfonamide metabolites or sulfonamide related compounds searched for in the effluent, river water and sediment could be detected (Table S2 and S3). This suggests that *sul2* may be a result of direct selective pressure by other antibiotic substances, or possibly a result of genetically linked co-resistance. Finally, there was also a surprisingly high diversity of mobile fluoroquinolone resistance genes where *qnrD*, *qnrS* and *qnrVC* were most abundant. To our best knowledge, these classes of *qnr* resistance genes have previously not been identified in India.

River sediment exposed to antibiotics show an enrichment of elements facilitating gene transfer. Integrons of class 1 and the recently described ISCR transposons were highly overrepresented, supporting the hypothesis that these enzymes are important elements for the flow of resistance determinants between environments [37]. Their importance is further emphasized by their ability to move larger regions of genetic material containing arrays of resistance genes. The proportion of resistance-carrying plasmid-associated DNA at the downstream sites was also notably high. These results suggest that the high levels of resistance genes are associated with different forms of elements for horizontal gene transfer.

The present study shows how metagenomics in combination with high-throughput sequencing forms a powerful way to detect high levels of resistance genes in bacterial communities exposed to antibiotics. However, due to the high biodiversity expected in soil and sediments, the 160 million DNA bases generated within this study is very likely to only cover a fraction of the entire metagenomes [40], [41]. The restricted sequencing depth will thus affect the statistical power of detecting less abundant resistance genes as well as assessing the frequencies of chromosomal mutations known to cause resistance. It also complicates the assembly of larger resistance plasmids which are in general present in lower copy numbers. However, as clearly showed, the sequencing depth applied in this study was sufficient to demonstrate significant differences in the resistomes of the investigated communities. Advances in high-throughput DNA sequencing and improved methods for *in silico* analysis of metagenomic data will be necessary to fully characterize these metagenomes and their associated resistomes.

Our results show that multiple classes of resistance genes are promoted in a highly antibiotic-contaminated environment. These results stress the role of the environmental microbial communities, in combination with unintentional antibiotic release, as potential recruitment pools for human pathogens. Further efforts to protect environmental bacteria against antibiotic pollution are therefore motivated.

## Materials and Methods

### Sampling and DNA sequencing

Samples were collected in a gradient up and downstream from a waste water treatment plant (WWTP) located in Patancheru, Hyderabad, India receiving process water from about 90 bulk drug manufacturers (6 samples, Table S1) and up and downstream from a WWTP located in Skövde, Sweden (2 samples, Table S1). Multiple samples (5–6) were collected at each site and pooled in order to minimize the spatial variability. Sequencing was done at GATC Biotech (Konstanz, Germany) using a Roche Genome Sequencer FLX Titanium [42]. Barcode-based multiplexing were used to sequence the eight samples simultaneously. Low-quality reads were removed according to the manufacturer's recommendation. Statistics from the sequencing is summarized in Table S4. The metagenomic data has been deposited at the NCBI Sequence Read Archive under accession number SRP002078. Full details regarding DNA amplification and sequencing are available in the Supporting Information (Text S1).

### Chemical analysis

The effluent from the Indian treatment plant has previously been screened for more than 200 different pharmaceuticals, solvents, pesticides and metals [11], [12], [43]. In this study we performed analysis of 8 fluoroquinolones and 46 sulfonamide and sulfonamide-like substances in the effluent, river water and sediment (Table S2, S3). These samples were extracted as previously described [12], [44] but with minor modifications (Text S1, Table S1). Fluoroquinolones were analyzed by liquid chromatography coupled to an ion trap mass spectrometer and electro spray interface (LC-ESI-IT-MSMS). Sulfonamides were analyzed by liquid chromatography coupled to a triple quadruple and heated electro spray interface (LC-HESI-TSQ-MSMS). Two or three selective reaction monitoring (SRM) transitions were monitored for each analyte and several calibration standards covering all concentration ranges were measured before, in the middle and at the end of each sample sequence. Full scan qualitative analysis was done using an LTQ Orbitrap MS at a resolution of 60.000 at full-width-half-maximum (FWHM) and by comparing the full scan spectra with calculated masses (Text S1). The chemical analyses of river sediments are given in *ug*/*g* organic weight and *ug*/*g* dry weight instead of *ug* /*g* sediment due to the high and variable proportion of solid material (gravel) in the samples.

#### Computational analysis and bioinformatics

All sequencing reads were compared against the non-redundant protein database (nr) at NCBI GenBank using BLAST 2.2.18^33^ using the E-value cut-off 10^−5^ (best hit used). Taxonomic affiliation was extracted from the NCBI Taxonomy database. Functional annotation was done by comparing the reads against the Clusters of Orthologous Genes (COGs) [45] and TIGRFAMS [46] databases using RPSBLAST [47] (E-value cut-off of 10^-5^). A database of resistances genes was created from (1) all sequences in Antibiotic Resistance Database (ARDB) [48] and (2) sequences from known quinolone resistance genes consisting of sequences from *qnrA-D*, *qnrS*, *qepA*, *acrA-B*, *norA-C* and *oqxA-B* [29], [32], [49]. A read was annotated as a resistance gene according to its best BLAST hit (blastx or tblastx) if (1) the hit had an amino acid sequence similarity above a given threshold and (2) if the alignment was at least 25 amino acids. Genes from ARDB used a similarity threshold according to their recommendation, for other genes the similarity threshold was set to 90%.

The metagenomics were searched for *qnr*-like resistance genes as follows. A position-specific scoring matrix (PSSM) was created by aligning sequences from the five classes of known qnr-,*qnrS1* (BAD88776) using ClustalW 2.0 [50]. All reads were compared to the PSSM using RPSBLAST (E-value cut-off 10^−10^) and then to all known mobile qnr-genes [29]. Reads with a significant hit to the *qnr* PSSM and that aligned with at least 25 amino acids with 90% sequence similarity to a known *qnr* gene were annotated as known. Note that known *qnr* genes matched the PSSM with an E-value of 10^−88^ or better while the corresponding E-values for the more general COG1357 where below 10^−15^.

The nucleotide sequences for all integrases available in the database INTEGRALL [51] were downloaded (1323 sequences) and grouped using single-link hierarchical clustering with a cut-off of 90% sequence similarity over at least 50 amino acids. Unknown integrases that clustered together with known integrases were re-annotated accordingly, resulting in 887 integrases of specific type and 436 integrases of unknown type. The integrase annotation pipeline was analogous to the resistance gene pipeline with an amino acid similarity threshold of 90%.

All sequences from the ISFinder [52] were downloaded (2565 sequences, 19 families of insertion sequences). All reads were compared against the collection of insertion sequences using BLAST. A read was annotated as an insertion sequence according to the best BLAST hit if (1) the hit had a sequence similarity above 80% and (2) if the alignment was at least 50 bases. For transposases associated with insertion sequence common regions (ISCR), we used the annotation suggested by Toleman *et al.* [37] http://www.cardiff.ac.uk/medic/aboutus/departments/medicalmicrobiology/genetics/iscr/iscr_elements.html) to extract 32 ISCR peptides (ISCR1-8 and ISCR14). The annotation pipeline was analogous to the resistance gene pipeline with an amino acid similarity threshold of 95%.

All sequence reads were aligned to (1) plasmid sequences available in the NCBI RefSeq database (2138 sequences) and (2) sequences in GenBank nucleotide database annotated as plasmids (\organism feature = “plasmid”) (9815 sequences). The alignment was performed using BLAT^41^ (“−fine −maxGap = 3” for maximum sensitivity) using the known plasmid as the reference sequence. The plasmid coverage was calculated by counting the number of reads aligned at each base. To remove effects from single point mutations and sequencing errors we used a 101 bases wide moving average to smooth the coverage (50 bases at each side). Then a conservative estimate of the total coverage was calculated using 25^th^ percentile of the coverage distribution.

*De-novo* assembly of novel plasmids was performed by concatenating overlapping in a step-wise fashion (using BLAST, minimum overlap of 200 bases and a 95% sequence similarity) until the fragment was found to be circular. After assembly, the plasmids were manually quality controlled and open reading frames (ORFs) were predicted using Glimmer 3.02 [53]. The plasmids were then verified by amplification using PCR with back-to-back primers (Text S1, Text S2 and Figure S6).

### Statistical analysis

All statistical calculations were done in R-2.8.1 (www.r-project.org). Tests for differences in taxonomic distribution were done using a *2*×*k* contingency table (χ^2^-test). Clustering of genus abundance profiles was performed by hierarchical clustering using the scale-invariant Pearson correlation metric. The logarithm of the number of observations was used to reduce the impact of highly abundant genera. Rarefaction curves were derived by randomly sampling reads from the metagenomes and calculating the number of unique genera [41], [54]. Each curve was calculated 100 times and smoothed using cubic-spline interpolation. The statistical analysis of gene-families, resistance genes and mobile elements was performed by ShotgunFunctionalizeR 1.03 using custom-made annotations and the Poisson model for direct comparisons [24]. To remove effects caused by unequal proportion of bacteria between samples (and thus decrease the intersample variability), we used the number of identified bacterial genes as the offset in the Poisson model. False-discovery rate (FDR) was used to correct for multiple testing and to estimate the number of false positives [55]. Gene-families with an FDR below 0.10 were considered significant.

## Supporting Information

Text S1 Supporting materials and methods.
(DOCX)

Text S2 Supporting data containing DNA sequencing of the novel plasmids pHIRE-D1 and pHIRE-U1.
(DOCX)

Figure S1 Taxonomic composition of the metagenomes from the eight sampling sites. The classification was assigned based on BLAST comparison against the NCBI GenBank non-redundant protein database (nr).
(PDF)

Figure S2 Rarefaction curves of the bacterial genera for the communities in India. Taxonomic affiliation was assigned based on BLAST comparison against the NCBI GenBank non-redundant protein database (nr).
(PDF)

Figure S3 Rarefaction curves of the bacterial genera for the communities in Sweden. Taxonomic affiliation was assigned based on BLAST comparison against the NCBI GenBank non-redundant protein database (nr).
(PDF)

Figure S4 Taxonomic distribution of highly abundant bacterial phyla. Taxonomic affiliation was assigned based on BLAST comparison against the NCBI GenBank non-redundant protein database (nr).
(PDF)

Figure S5 Abundance profiles for the bacterial genera in the sediment samples. The relations between the samples were calculated by hierarchical clustering using Pearson correlation distance metric.
(PDF)

Figure S6 PCR-verification of the novel plasmids using back-to-back outward-facing primers. As predicted, pHIRE-D1 (5174 bases) was found in the two tested downstream sites while pHIRE-U1 (2682 bases) was found in one of the upstream sites.
(PDF)

Table S1 Detailed information regarding the sampling sites.
(PDF)

Table S2 Chemical measurements of antibiotics in the river sediments. The results are given as analyte per organic weight (ng/g). The numbers in the parenthesis is the standard deviation. Substances marked with ^*^ were only measured qualitatively.
(PDF)

Table S3 Chemical measurements of antibiotics in the river sediments. The results are given as analyte per dry weight (ng/g). The numbers in the parenthesis is the standard deviation. Substances marked with ^*^ were only measured qualitatively.
(PDF)

Table S4 Results from the massively parallel pyrosequencing. % is given in relation to the total number of reads.
(PDF)

Table S5 Abundance of bacterial genera in the sediment around the Indian and Swedish waste water treatment plants.
(PDF)

Table S6 Significant clusters of orthologous genes (COG) between sites up and downstream from the Indian treatment plant.
(PDF)

Table S7 Significant clusters of orthologous genes (COG) between the upstream sites at the Indian treatment plant and the sites at the Swedish treatment plant.
(PDF)

Table S8 Significant clusters of orthologous genes (COG) between the downstream sites at the Indian treatment plant and the sites at the Swedish treatment plant.
(PDF)

Table S9 Significant TIGRFAM gene families between the sites up and downstream from the Indian treatment plant.
(PDF)

Table S10 Significant TIFRFAM gene families between the Indian upstream sites and the sites up and downstream from the Swedish treatment plant.
(PDF)

Table S11 Significant TIGRFAM gene families between the Indian downstream sites and the sites up and downstream from the Swedish treatment plant.
(PDF)

Table S12 Resistance genes identified in the metagenomes.
(PDF)

Table S13 Elements associated with horizontal gene transfer identified in the metagenomes. The insertion sequence family name is given in parantheses where applicable.
(PDF)

Table S14 Resistance genes and mechanisms of horizontal gene transfer with a significantly different relative abundance between the Indian up and downstream metagenomes.
(PDF)

Table S15 Resistance genes and mechanisms of horizontal gene transfer with a significantly different relative abundance between the Indian upstream and Swedish metagenomes.
(PDF)

Table S16 Resistance genes and mechanisms of horizontal gene transfer with a significantly different relative abundance between the Indian downstream and Swedish metagenomes.
(PDF)
