# Supplementary material for: Pyrosequencing of Antibiotic-Contaminated River Sediments Reveals High Levels of Resistance and Gene Transfer Elements
Source: PLoS One. 2011 Feb 16;6(2):e17038. doi: 10.1371/journal.pone.0017038 (PMC3040208; doi:10.1371/journal.pone.0017038)
Supplement: Text S1 — Supporting materials and methods. (DOCX) [file pone.0017038.s001.docx]

**Supporting materials and methods**

***Sample collection***

Samples were collected in a gradient up and downstream from a waste water treatment plant (WWTP) located in Patancheru, Hyderabad, India (table S1) and up and downstream from a WWTP located in Skövde, Sweden. Multiple samples (5 to 6) were collected at each site to minimize the spatial variability.

***DNA extraction and sequencing***

The DNA extraction was performed by using the MoBio PowerSoilTM DNA Isolation Kit (Solona Beach, CA). To complete homogenization and cell lysis the power bead tubes with sediment and solution C1 were incubated at 70 °C for 10 min. with a briefly vortex after 5 and 10 min. The DNA concentration was measured using a Nanodrop (Thermo Fisher Scientific Inc) and retained at -80 °C until the amplification of purified genomic DNA was performed. The amplification was done using REPLI-g mini kit (Qiagen). 10 ng of DNA was lysed and denaturated by adding a denaturation buffer and was then incubated for 3 min at RT. The denaturation was stopped by a neutralization buffer and a master mix containing a reaction buffer and DNA polymerase was added. The amplification reaction was performed by incubating the samples at 30 °C for 16 h in a thermal cycler and the reaction was then stopped by heating for 3 min at 65 °C.

Sequencing was done at GATC Biotech (Konstanz, Germany) using a Roche Genome Sequencer FLX Titanium (*1*). Barcode-based multiplexing where used to sequence the eight samples simultaneously. Low-quality reads were removed according to the manufacturer’s recommendation. See table S4 for statistics from the sequencing.

***Validation of novel plasmids***

To confirm the presence of the novel plasmids with PCR, back-to-back outward-facing primers (with adjacent 5’ ends) were designed inside the putative mob gene in pHIRE-D1 and the *qnrD* gene in pHIRE-U1 allowing amplification of circular or repetitive linear plasmid sequences.

| Primer name | Sequence |
| --- | --- |
| pPHIRE-D1-mob-F1 | 5'-GGGCGGCACAATCGACTA-3' |
| pPHIRE-D1-mob-R1 | 5'-TGTGGTCGTTGTCTGCCGT-3' |
| pPHIRE-U1-mob-F1 | 5'-TTCGTGGCTCCGATCTTTCT-3' |
| pPHIRE-U1-mob-R1 | 5'-ACACGGCGCCAGTTATCA-3' |

The 5 or 6 REPLI-g amplified DNA samples from each of the Indian sites were pooled and diluted to 50 ng/μl. Long-range PCR was then performed using the Platinum Pfx proofreading enzyme (Invitrogen, Carlsbad, CA, USA) and the above primers on DNA from each of the Indian sites. Each 50 μl PCR reaction mixture contained a final concentration 0.3 mM each dNTP, 1 mM MgSO4, 0.3 mM each primer, 250 ng template DNA and 1 U Pfx enzyme in 1X Pfx Amplification Buffer. PCR amplification was performed through 45 cycles of 30 s at 94 °C, 30 s at 55 °C and 12 min at 68 °C with a 5 min initial denaturation step at 94 °C and a 20 min final extension step at 68 °C. PCR products were analyzed with agarose gel electrophoresis.

***Chemical analysis***

*Chemicals*

Ciprofloxacin, difloxacin, enoxacin, enrofloxacin, kanamycin, lomefloxacin, netilmycin, norfloxacin, ofloxacin, pefloxacin, ribostamycin, streptomycin, sulfanilamide, sulfadimethoxine, sulfamerazine, sulfamethazine, sulfamethizole, sulfamethoxazol, sulfamethoxypyridazine, sulfamoxol, sulfaphenazole, sulfapyridine and sulfathiazol were of analytical grade (> 98 %) and obtained from Sigma Aldrich. Acetonitrile, methanol, and water (Lichrosolv, Hypergrade) were obtained from Merck and formic acid (puriss pa) was obtained from Fluka. C13 labeled surrogate standards; 13C4-ciprofloxacin, and 13C6-sulfamethoxazol were obtained from Cambridge Isotope Laboratories (Andover, MA, USA).

*Soil extraction*

One gram of the sediment samples were extracted using a method described previously (2), however the samples were extracted with a solution of water containing 25% methanol, 5% triethylamine and 5% ethylenediaminetetraacetic acid. 50 ng of each surrogate standard was added to the sediment before extraction.

*LC-HESI-TSQ-MSMS*

Triple stage quadrupole MS/MS TSQ Quantum Ultra EMR (Thermo Fisher Scientific, San Jose, CA, USA) coupled with an Accela LC pump (Thermo Fisher Scientific, San Jose, CA, USA) and a PAL HTC autosampler (CTC Analytics AG, Zwingen, Switzerland) were used as analytical system for quantification of sulfonamides.

Five µL of the sample was loaded by 5 µl loop onto a Hypersil GOLD aQ TM column (50 mm x 2.1 mm ID x 5 µm particles, Thermo Fisher Scientific, San Jose, CA, USA) preceded by a guard column (2 mm×2.1 mm i.d, 5 µm particles) of the same packing material and from the same manufacturer.

A gradient of flow and MeOH and ACN in water (all solvents buffered by 0.1% formic acid) was used for elution of analytes. The elution conditions were programmed as follows: 200 µL min-1 100% water for 1 min isocratically, then composition is changed to 20/30/50 water/ACN/MeOH and flow of 300 µL min-1 at 8 min. Then the column was washed by 100% ACN at a flow of 300 µl min-1 for 0.5 minutes and thereafter100% water at a flow of 300 µl min-1 for 4 minutes.

Heated electrospray ionization (HESI) in positive ion mode was used for ionisation of target compounds. The setting of key parameters was as follows: ionisation voltage 3.5 kV, sheath gas 50 and auxiliary gas 35 arbitrary units, vaporiser temperature 100 C, capillary temperature 325 C, collision gas was argon at 1.5 ml min-1. Both first and third quadrupole were operated at resolution 0.7 full-width-half-maximum (FWHM). Two or three SRM transitions were monitored for each analyte. Mass transition and parameters are shown in the table below. Scan time for single compound method was set 100 ms for IS transitions and 200ms for native compounds transitions. In method, where three compounds were analyzed together, scan times 40 ms and 80 ms were used. Samples were quantified using internal standard method. Several calibration standards covering all concentration range were measured before in the middle and at the end of sample sequences. The maximum difference between results at quantification and qualification mass transition was set to 30% as criterion for positive identification.

*LC-ESI-IT-MSMS*

Ion trap MS/MS LCQ Fleet MS (Thermo Fisher Scientific, San Jose, CA, USA) coupled with a Surveyor LC pump (Thermo Fisher Scientific, San Jose, CA, USA) and a Thermo autosampler (Thermo Fisher Scientific, San Jose, CA, USA) were used as analytical system for quantification of fluoroquinolones.

Twenty µL of the sample was loaded by 20µl loop onto a Hypersil GOLD aQ TM column (50 mm x 2.1 mm ID x 5 µm particles, Thermo Fisher Scientific, San Jose, CA, USA) preceded by a guard column (2 mm×2.1 mm i.d, 5 µm particles) of the same packing material and from the same manufacturer.

A gradient of flow and MeOH and ACN in water (all solvents buffered by 0.1% formic acid) was used for elution of analytes. The elution conditions were programmed as follows: 200 µL min-1 10% methanol in water for 1 min isocratically, then composition is changed to 30/10/60 water/ACN/MeOH and flow of 250 µL min-1 at 8 min. Then the column was washed by mixture ACN/MeOH 60/40 and flow of 300 µl min-1 in 9 minutes. These parameters were kept for 1 min and then they were switched to starting conditions and let 4 min to equilibrate for next run.

Electrospray ionization (ESI) in positive ion mode was used for ionisation of target compounds. The setting of key parameters was as follows: ionisation voltage 4 kV, sheath gas 50 and auxiliary gas 5 arbitrary units, capillary temperature 325 C, collision gas was helium at 1.5 ml min-1and a resolution 1.0 FWHM was used. Two or three SRM transitions were monitored for each analyte. Mass transitions, collision energies and tube lens voltage are shown in the table below. Scan time for single compound method was set 100 ms for IS transitions and 200ms for native compounds transitions. In method, where three compounds were analyzed together, scan times 40 ms and 80 ms were used. Samples were quantified using internal standard method with labeled ciprofloxacin as surrogate standard. Several calibration standards covering all concentration range were measured before in the middle and at the end of sample sequences. The maximum difference between results at quantification and qualification mass transition was set to 30% as criterion for positive identification.

*LC-HESI-LTQ-HR-MSMS*

High Resolution MS/MS was performed using a LTQ Orbitrap XL MS (Thermo Fisher Scientific, San Jose, CA, USA) coupled with a Accela LC pump (Thermo Fisher Scientific, San Jose, CA, USA) and a Thermo autosampler (Thermo Fisher Scientific, San Jose, CA, USA) were used as analytical system for qualitative analysis of antibiotic residues in the samples.

Five µL of the sample was loaded by 5µl loop onto a Hypersil GOLD aQ TM column (50 mm x 2.1 mm ID x 5 µm particles, Thermo Fisher Scientific, San Jose, CA, USA) preceded by a guard column (2 mm×2.1 mm i.d, 5 µm particles) of the same packing material and from the same manufacturer.

A gradient of flow and MeOH and ACN in water (all solvents buffered by 0.1% formic acid) was used for elution of analytes. The elution conditions were programmed as follows: 200 µL min-1 100% water for 1 min isocratically, then composition is changed to 20/30/50 water/ACN/MeOH and flow of 300 µL min-1 at 8 min. Then the column was washed by 100% ACN at a flow of 300 µl min-1 for 0.5 minutes and thereafter 100% water at a flow of 300 µl min-1 for 4 minutes.

Heated electrospray ionization (ESI) in positive ion mode was used for ionisation of target compounds. The setting of key parameters was as follows: ionisation voltage 5 kV, sheath gas 60 and auxiliary gas 40 arbitrary units, vaporiser temperature 200 C, capillary temperature 350 C, collision gas was helium at 2 ml min-1. Analysis was done in full scan (50-500 m/z) and at a resolution of 60000 FWHM (3).

Qualitative analysis of all sediment samples was performed by comparing the full scan spectra with calculated accurate masses (shown in the table below). Qualitative analysis was also done of all samples of river surface water and treated effluent from a previous investigation (4). Tentatively limit of detection was estimated at 100 ng/L based on responses of surrogate standards.

*Mass transitions and parameters used in the quantitative analytical methods.*

|  | PREI ^a^  *m/z* | PROI ^b^  *m/z* | CE ^c^  *V* | TL ^d^  *V* | LoQ_Soi_l ^e^  *ng g^-1^* | LoQ_Water_ ^f^  *ng L^-1^* |
| --- | --- | --- | --- | --- | --- | --- |
| Sulfamethoxazol | 254 | 92.2 | 28 | 108 | 10 | 1 |
|  | 254 | 108.2 | 22 | 108 |  |  |
|  | 254 | 156 | 15 | 108 |  |  |
| Sulfamethoxazol ^13^C_6_ | 260 | 98.3 | 25 | 97 |  |  |
|  | 260 | 114.2 | 23 | 97 |  |  |
|  | 260 | 162.1 | 15 | 97 |  |  |
| Sulfanilamide | 173.0 | 156.2 | 10 | 79 | 10 | 1 |
|  | 173.0 | 65.3 | 38 | 79 |  |  |
|  | 173.0 | 76.0 | 41 | 79 |  |  |
| Sulfapyridine | 250.0 | 156.1 | 17 | 75 | 10 | 1 |
|  | 250.0 | 184.2 | 17 | 75 |  |  |
|  | 250.0 | 92.3 | 28 | 75 |  |  |
| Sulfathiazol | 256.0 | 156.1 | 15 | 90 | 10 | 1 |
|  | 256.0 | 92.3 | 26 | 90 |  |  |
|  | 256.0 | 108.2 | 26 | 90 |  |  |
| Sulfamerazine | 265.0 | 92.2 | 29 | 83 | 10 | 1 |
|  | 265.0 | 108.3 | 25 | 83 |  |  |
|  | 265.0 | 172.1 | 17 | 83 |  |  |
| Sulfamoxol | 268.1 | 156.2 | 15 | 83 | 10 | 1 |
|  | 268.1 | 92.3 | 27 | 83 |  |  |
|  | 268.1 | 108.2 | 24 | 83 |  |  |
| Sulfamethizole | 271.0 | 156.1 | 14 | 78 | 10 | 1 |
|  | 271.0 | 92.3 | 27 | 78 |  |  |
|  | 271.0 | 108.2 | 23 | 78 |  |  |
| Sulfamethazine | 279.1 | 186.1 | 17 | 77 | 10 | 1 |
|  | 279.1 | 124.3 | 22 | 77 |  |  |
|  | 279.1 | 92.3 | 29 | 77 |  |  |
| Sulfamethoxypyridazine | 281.0 | 156.1 | 17 | 86 | 10 | 1 |
|  | 281.0 | 92.2 | 29 | 86 |  |  |
|  | 281.0 | 108.2 | 26 | 86 |  |  |
| Sulfadimethoxine | 311.0 | 156.2 | 21 | 92 | 10 | 1 |
|  | 311.0 | 108.2 | 30 | 92 |  |  |
|  | 311.0 | 92.3 | 31 | 92 |  |  |
| Sulfaphenazole | 315.1 | 158.2 | 28 | 93 | 10 | 1 |
|  | 315.1 | 160.2 | 23 | 93 |  |  |
|  | 315.1 | 131.2 | 42 | 93 |  |  |
| Ciprofloxacin | 332.1 | 314.1 | 30 | 70 | 20 |  |
|  | 332.1 | 288.2 | 30 | 70 |  |  |
|  | 332.1 | 268.3 | 30 | 70 |  |  |
| Ciprofloxacin ^13^C_4_ | 336 | 291.1 | 27 | 70 |  |  |
|  | 336 | 318.1 | 27 | 70 |  |  |
| Difloxacin | 400.2 | 356.1 | 33 | 100 | 20 |  |
|  | 400.2 | 382.1 | 33 | 100 |  |  |
| Enoxacin | 320.5 | 276.1 | 28 | 90 | 20 |  |
|  | 320.5 | 302.1 | 28 | 90 |  |  |
| Enrofloxacin | 360.2 | 316.1 | 34 | 105 | 20 |  |
|  | 360.2 | 342.1 | 34 | 105 |  |  |
| Lomefloxacin | 352.3 | 308.1 | 37 | 110 | 20 |  |
|  | 352.3 | 265.1 | 37 | 110 |  |  |
|  | 352.3 | 288.1 | 37 | 110 |  |  |
| Ofloxacin | 364.2 | 318.2 | 30 | 45 | 20 |  |
|  | 364.2 | 344.1 | 30 | 45 |  |  |
| Pefloxacin | 334.3 | 290.1 | 26 | 95 | 20 |  |
|  | 334.3 | 316.1 | 26 | 95 |  |  |
| Norfloxacin | 320.5 | 277.1 | 28 | 90 | 20 |  |
|  | 320.5 | 303.1 | 28 | 90 |  |  |

^a^ Precursor ion, ^b^ Product ion, ^c^ Collision energy, ^d^ Tube lens, ^e^ Limit of quantification in soil samples, ^f^ Limit of quantification in water samples

| Name | Molecular formula | Accurate mass ^a^ |
| --- | --- | --- |
| Sulfabenzamide | C13H12N2O3S | 277.0647 |
| Sulfacarbamide | C7H9N3O3S | 216.0443 |
| Sulfacetamide | C8H10N2O3S | 215.0490 |
| Sulfachlorpyridazine | C10H9ClN4O2S | 285.0213 |
| Sulfachrysoidine | C13H13N5O4S | 336.0766 |
| Sulfaclozine | C10H9ClN4O2S | 285.0213 |
| Sulfadiazine | C10H10N4O2S | 251.0603 |
| Sulfadicramide | C11H14N2O3S | 255.0803 |
| Sulfadimethoxine | C12H14N4O4S | 311.0814 |
| Sulfadimidine | C12H14N4O2S | 279.0916 |
| Sulfadoxine | C12H14N4O4S | 311.0814 |
| Sulfafurazole | C11H13N3O3S | 268.0756 |
| Sulfaguanidine | C7H10N4O2S | 215.0603 |
| Sulfamerazine | C11H12N4O2S | 265.0759 |
| Sulfamethizole | C9H10N4O2S2 | 271.0323 |
| Sulfamethoxazole | C10H11N3O3S | 254.0599 |
| Sulfamethoxypyridazine | C11H12N4O3S | 281.0708 |
| Sulfamethylthiazole | C10H11N3O2S2 | 270.0371 |
| Sulfametomidine | C12H14N4O3S | 295.0865 |
| Sulfametopyrazine | C11H12N4O3S | 281.0708 |
| Sulfametrole | C9H10N4O3S2 | 287.0273 |
| Sulfamonomethoxine | C11H12N4O3S | 281.0708 |
| Sulfamoxole | C11H13N3O3S | 268.0756 |
| Sulfanilamide | C6H8N2O2S | 173.0385 |
| Sulfapyridine | C11H11N3O2S | 250.0650 |
| Sulfaquinoxaline | C14H12N4O2S | 301.0759 |
| Sulfathiazole | C9H9N3O2S2 | 256.0214 |
| Sulfathiourea | C7H9N3O2S2 | 232.0214 |
| Sulfatroxazole | C11H13N3O3S | 268.0756 |
| Sulfisomidine | C12H14N4O2S | 279.0916 |
| Acetyl-Sulfadiazine | C12H12N4O3S | 293.0709 |
| Acetyl-Sulfadimethoxine | C14H16N4O5S | 353.0921 |
| Acetyl-Sulfamethazine | C14H16N4O3S | 321.1022 |
| Acetyl-Sulfamethoxazole | C12H13N3O4S | 296.0706 |
| Acetyl-Sulfathiazole | C11H11N3O3S2 | 298.0321 |
| Pterine-sulfathiazole | C16H13N8O3S | 429.0552 |
| 7,8-Dihydropterine-sulfathiazole | C16H15N8O3S | 431.0709 |
| 4-Hydroxy-sulfathiazole | C9H11N3O3S2 | 274.0321 |
| 4-Amino-2-hydroxybenzoic acid | C7H7NO3 | 154.0504 |
| Para-Aminobenzoic acid | C7H7NO2 | 138.0555 |
| 4-Acetylamino- benzenesulfonyl chloride | C8H8ClNO3S | 233.9992 |
| 4-Acetylamino- benzenesulfonic acid | C8H8NO4S | 215.0253 |

^a^ [M + H]^+^

**References**

1. Margulies M*, et al.* (2005) Genome sequencing in microfabricated high-density picolitre reactors. (Translated from eng) *Nature* 437(7057):376-380 (in eng).

2. Lindberg RH, Wennberg P, Johansson MI, Tysklind M, & Andersson BA (2005) Screening of human antibiotic substances and determination of weekly mass flows in five sewage treatment plants in Sweden. (Translated from eng) *Environ Sci Technol* 39(10):3421-3429 (in eng).

3. Kellmann M MH, Zomer P, Mol H. (2009) Full Scan MS in Comprehensive Qualitative and Quantitative Residue Analysis in Food and Feed Matrices. *Journal of the American Society for Mass Spectrometry* 20:13.

4. Fick J*, et al.* (2009) Contamination of surface, ground, and drinking water from pharmaceutical production. (Translated from eng) *Environ Toxicol Chem* 28(12):2522-2527 (in eng).
