# Supplementary material for: Pyrosequencing of Antibiotic-Contaminated River Sediments Reveals High Levels of Resistance and Gene Transfer Elements
Source: PLoS One. 2011 Feb 16;6(2):e17038. doi: 10.1371/journal.pone.0017038 (PMC3040208; doi:10.1371/journal.pone.0017038)
Supplement: Text S2 — Supporting data containing DNA sequencing of the novel plasmids pHIRE-D1 and pHIRE-U1. (DOCX) [file pone.0017038.s002.docx]

**Supporting Data**

***Plasmid sequences***

>pHIRE-D1

TGGGTGAAAAGGGCGTTTATTCTTCCTATACGTTGTCGGCAGCGGGCCAAAAAGGAATACGTCCATGCCCATCGAGGTGAAACCGGCTGTGAGCGCGGGTTCAAGCATATAGCCCGACAGGCGCGTATCCTTGCCGATCACGACACGATGGCGGTGGTCACCGCGACGAAAGACACGGCCAGCCGCCATGCCGACGCGCAAGGCGGTTTCCGCCGTCATCGCGCCTTCGTTGGCTTTGCCACGAATACCGTCTGTGCCGAAATATTTGCGCACCATAAGGTCGATTATCCTGTCGTCGGGTCGCCCTCAAAGGGGACATGCCTGCTGAACCGCGAATATAGAGAAATATCCCGAATGTGCAGTTAACGAATTCTTGCGGTTTCTTCAGCGCCGCCAATACCGCCAGCCCGTCGCGCAAGGGGCGCGGCTCGTGTGTGCGGATGAAGTCAGCTCCACCTGCGGCGGCGGCAAGCTCTGCAGCGAGTGTCGCGGCCCCGACATCCCCCGGACCACGGCCTGTGAGCGCGCGCAGAAAGGATTTGCGCGAAACAGACAGAAGCACCGGCAAATCGAAGCGCACGCGCAATTCATCGAACCGCGCCAGCACCGAGAGCGAGGTTTCGGGAGCAGCCCCCAGAAAAAACCCCATGCCGGGATCAAGGACAAGGCGGTTGCGTTTGATACCGGCACCCGTCAGCGCCGCGATGCGCGCGTCAAAGAACGCCGCAATGTGATCCATGATGTCGCCAGCGGGTGCCTCGCGCCGATCTGCCTGCCCGTCTTGCACCGAATGCATAACGACGAGTTTGGCAGATGATTTCGCCAATTGCGGATAGAACGCAGCGTCTGGAAAACCGCGAATATCATTGAGATAGGCCACACCACGCGACAAGGCATAGGCTTGCGTCGCGGGTTGATAACTGTCGAGCGAGACGGGAATGCCATCTGCCTTGAGCGCGTCCAGCACCGGCGCGA

TACGCGCGATTTCTGTGTCGGACGAAACAGGCGCGGCGTCGGGATTGCTGGATGCCGGACCGAGGTCGATCACATCTGCCCCCTCGGCCATCAGCTTACGCGCCTGCGCAATGGCTGCGTCTGGCGCCAGATACCGGCCTCCATCGGAGAAACTGTCCGAGGTTATGTTGACGATGCCGAAAATGATGAGCGATTTATTCATGGGGGCTTCTATAATAATAATAATCGAGCATGAGTCTCATACGGATGCTCGGGTCGAAAGGGAATCCCCAGGCGAGTAACCTGTTTGCGGTGATCCATTAGCTGCAGGAGCAGAAGAGCATACATCTGGAAGCAAAGCCAGGAAAGCGGCCTATGGAGCTGTGCGGCAGCGCTCAGTAGGCAATTTTTCAAAATATTGTTAAGCCTTTTCTGAGCATGGTATTTTTCATGGTATTACCAATTAGCAGGAAAATAAGCCATTGAATATAAAAGATAAAAATGTCTTGTTTACAATAGAGTGGGTTTAGCCGTTAATGATACCTTTTTGCTACTTCTATGCTACCTGATGCTACCTTTTTGCTACTTTTATGCTACCTGATGCTACTTTTCTGCTACTTTTATGCTACCTGATGATACTTTTATGCTACCCGCAAAGGTTTGCACAATTCAGCAAGTTTAGAACATAGGCACGTTATGGATTGTATACAATCCCGTGCGAAAGGAGAGCCTTTCAAACCCGAAAACCATAGCCCAGACACACCAGTCGGGGCTATGGTTTTTTAGCCTCTACGAGTCTAGTTACGGTGGGGTGCAATTGTTGCAATCGCTTAGCGATTCGTCATC

GTTCCAGTGCAGCTCTTCACCGCAATCGCACCAAACTCTAAAATTGTCATCATCACGAACATGGTGCGTTGCGATAGTAATGATGTCGTCTAACTGTAAACCTTCGATAAGTCGTTGCTGCCCTTGGGCTTCAAGCTGTTCTTGTTCGGTCAGTTCTGCCTGCTTTTCAGGGCATTCAGGGAGGTGAAGACTAGGCGGCAGTTTCGACCGTGTGATGTGGGTTGGTGGTTCTACTGGCTCGTCATCATCCAAAAGCAAGTAGCGGCCAGTTTTTCGCTTAGTGCACTCCGTGGCTGTGCCGTGACTTCCTCACTGGGTACGCTCCAGAGCGTCACACCATCGAGGTTTAAGCCGTCCATATCGGCCAAAGTGAAAACACCGGCCTCGCTTTTATTTGGCGTATAACCAAAGCCGTTAGGCAACCAGTAGCCCTTGTGGCTTTCAATGCGATAATAAGTCATGGCTCAATCCTCAAGCAGATTTTCATATGCTTGCTTGAAATGTTTTGCTGGTATTGGCTCACCGTGAAAGAGGATTCGTTTTTCTTTGAACGCTTCTTTTGCCTGCAATCGTCTGGCGTTATTGAGCAGCTCAAGCAAACCAACTTCAACTAGCAATGTGCGCAATTTTCGTTCACTTACATTGCAAGCAATGGCTATTTCTTCAATGAGATTTTTCACACTAAATTTCCTCTAAGCGTTGCTGCACTTGGGCTTCAAGTTGTTCTTGCTCGGTTAATTCAGACTCTCTTTTTCCGCTCAATCACTGTAGTAAATGCGATCCTGTCACCGTTAAACACGAAGTTTGGATTGATGAAATAGCGGC

CTTGGCGCATGGTTTTAGCGACAATTTGAGCCTTTTCCATTTCATTGATACCGCGCTTAAAAGTGGCAAGGCTAAGCTTTAAAGGCTTTAGTGTTTTTATGAACGTCCAAAAATTCTTCTAGAGTTAGGCTATCCAAGTCCACTTCATCTTTTGATAATGCTCTGTTTGTACTACCCAAAGCAGCACACCAAAAGCCTTGATACCTGCGGCTGAAAGGCCAAAAGTTAGTCCTATATTTGCTGTAAATAGCTTTACAAACTGTTCACCATCAACGCGCTTAAAAGTGGTTACGTGCGTTCCTTGAACCTCTCCTGTGGATTGGTTCACGAGTATGTTTTCATCACGTCCTAGTCGTGACAATCTTACTTGTCTGCCCTTGACTGGAACAATCATGTCATCAAGAAATGGATTTGTTTTGTAGCGAGAGAGGCTCATTTTAATACCCTAAATTGATCGACTGAATTCGATTTTAGATAGAGGGTAGCTCAAAATCAAGTTATTTTTTGAGCTACCGTAGTATGTAGATTGAGCTACCGTAGTACAGGTTCTGAAAAGTGGGTCTAGTATTTTATGCGGGTTACAGGAGTTTCCCTCTTAGTTCTTATTCGGGGCGAATAAGAACCCTCTAAGGGGCGTTGCCCCGCCAGCGCTTCGCTTGGCACCCCAAAAACAAATAATGCCTTCGCTTCGCTCAGAAAAATTTTTGTTACTTGCAGCGGCCTACGGCCTCGCTCTAAGGGGGCGTTGCCCCCTAGCGCGCTTCGCTTGCCTCCCCCACGTTCGCCACGTGGCAGGGGTGCATGGCGGGCGGGCTTCGCCCTACT

TCATGCCCCCTACCACCAAGCGTTGGTCGCTACGGCCAGCATCAAGGGTGCGGCTTCGCCCGAAATGCTCACCCGTTCGGTGCTCGGGCTGCGCCCTGCGCTCCGCGTGCGGCTTCCGCTTTCGCCCTTGACACTGTCCTACGCTCAGCTGTTTGAACGGAAAGGGGGTCAAGTGACCGTGGAATAAATAGCGCCCTTGGGCGCGGTTTATGCCGCGAAAGCACTTGATGCCCTTGGAGTGACTACACGCTTGGGCATGGGGGCAGGTAGAGCGAAGCGCAGCGCAGCGGCCACCTGCACCCCTGCCACGTGGCGAACGGACAAGCCCCCTTGGGGGGCGGCGTCAGCGGGGGATGCAACGAGTGAAACGAGGCGCAGGGCGCAGCCCGTAGAGCAAGGCCGTAGGCCGCTGCAACCCGCTTGCGGGTCACGATTTTACGAAGTAAAGTCTAGTGAGTACTCTTTGAAACAAAGAGGCCCGCCGGAGGCACTGCGTTTTCCGTCGGGATTGCACCAGCACAGACCGAGGGGGAACGCATGGCTTTTGCGATCATGAGAGCGAAAAAGCTCAACGGCATGGGGGCTGTCGCCGCCTCCCTGCAACACTGTTACAGGGATAGGGACACACCCAATGCCGATGCCGAGCGAACGGCAGACAACGACCACAGGGCGGCACAATCGACTAATGAGGCGATGGGCAAGCTGAGGGAGTTGTTGCCAGAGAAGCGGCGCAAGGATGCGGTGCTGGCGGTTGAGTACGTCATGACGGCATCACCGGAATGGTGGACGAAAGCGACCAGCGAACAGCAGGCGGATTTCTTCGAC

CAGGCGCACAAGTGGCTAGCCGATAAATACGGAGCTGACCGCATTATCACGGCCACGGTTCATAGGGATGAAACCAGCCCACACCTGAGCGCGTTTGTGGTGCCACTGACGGCTGACGGTCGCTTATCCGCCAAAGACTTCATCGGCAACCGCAGCAAAATGACAGCTGACCAAACCAGTTTTGCCAAGGCCGTGCAGCATTTGGGGCTTGAGCGTGGCATCGAGCGCAGTCGAGCCACTCACACCAGCATCAAACAGCACTATGCAGCGATTGAGAGGGGCATTAAAAATCAAGTTGAAATCAGCCCCGAAGCCTTAGAGCCACGGGCTTATAAGCGTGAGGGGTTAGCTGAAAAGTTGCGATTAACAACCCGTGTTGAAAGTACGGAAGCCGTAGCGGCTCGTTTATCGGCATCAGTGAATGAGGGGTTTGCGGGTACGATTGCAAATGCCGCTGAAAGCGCCCAGAACGCCCGTAGAGCGCGAGAAATTCAAAAGACAATGGTTGACCAGCAGAAGAAGCTAAAAACGCTTCAGGAGCCGTTTAAAGGGCTTTCACGCGAACAGGTAGCGGAGGTCATCAAGATGGCCACAACCATGCAGCGCGAAAACGAAGCGGCCAAGGCCGCGAAGTTGCAACACCAAAAGGAACAACGACAGCAGCAACGCAGCAAAAACAGGGGGAATGATTCCGCGTCTGGCACAGCCAGGCACCTGATGGCATGAAATGCTGCGTTCACAGCGCCGCGGCAGGGATCCGCCGTGCTGGTTGTCGGAAAAGGAGCCGCTAGTGGGAAAGAGGAGGGTAAATTTTCAGCGTTGCTG

GCTCCCCGTCAGCCGGATTGGGTTGCATCGCAGGGGTGTCGAAAGAGTCAACTGCGGTCCAAAGCTGTTGGACT

>pHIRE-U1 TTGTGATTTAACGGGTGGGGCGCTTGGCGAGCTTGATGCAAGGCGAATTAATTTAGATGGAGTGAAGTTGGATGGAGAGCAGGCGCTTCAGCTTGTTGAGAGTTTAGGTGTTATTGTTCACCGATAAAATCTAGGTAAAAAACGCCTAATGCCCCAATGTGGTACTAATCAAAACGGAGCGAAGCGACATCTTCATCTTGTCTTTGCTTTTCTCCATGATCAGTTATTGGGTCATGGTCTCTTAGCTCGTTAATCTGGCTAAGTTTGGTTAGGTTGGGCTTCTTTCGACACTTCGCAATCCAAGAAATCCCTAGTCCGTTGATTGGTGCTCTTATCAGGTACGCACTGTAATCTTTCGATACCGCAGCGTGATTCGGAGGTAGAGCCGGAATTTCGTTTTCCGGTACGTTTTACTACCACAATTTGCCTGTTTATCCTTTCGGAGACCGGGCTAAGTCCGAGTCAATCAAGTGGTGCATTTGGGTTAATGTCGTTGGCTGGTATTTAATGAGAACGTGACAAGCGCCTCTCACGTTGCTAAGATTGGATTGCGACGTCTAATCTGAGCAACGAATGCACAAGATTTAGAGTACCAGAGCC

CTGTGATGCTGTCACGGGGCTTTGTTACATCTGGAGCTTGGTAAACCTCATAAATTCAATTCGCAACGCTCGCCGCAAGGCGTGAACTTGTTTCACGTCGAGGAAGCGGAATTTCTCGAAAGCCTTATACTGTCTCCGTTCACACATGATCGGAGGGTGTCTCTATGTCTCAATTATTTCGTTTGGCATCGCCAGAAGATTACGAATACATCGAAGAATTAAATTTATGGGAATTGTCATTTGATAAGCGACCGGTTCAAGGGGTTCGTTGTGAAGATCCGGTGATCGGGGCAGAAAAATATAATCAAGGGCGTGAAAAATTTAAGGCGTTAGCGTCACGGCATAAAAAACACTGGAAACCGAACGGATTGACCTTTTCGGAGTTGATCCGCTGGGCAATAGAATGCGGAACTCCAGAACAATGTCAGCTTGTTTTAGCGTTATACCATTGCACAAACGATGATGATTATCAGGCGATGGGAATTCAGTTAAGCCGAAGTATTGATAATGCAAATGTCAGTCCGATCATCTTTTTCACAGGTAAAAATCAACGGTTATTAGGTGATATTTCGATGCTAAAACAGCTCGAAGAAAAGGTGC

GCTGACGTTAGGTTTACCTATTATTTTATGTATTATCCTATCTATTATTCTATCTCTTATTTTATCTATTTCGTCTGTAATAGGTAGAATAATACATAGAATAAGATACAGAATAATAGATAGACACAAGATGACGTTGTGTTATTTCTTACCCATTAATCCACCTGTTATTCTACCTATTACTTTACCCGTTATTGCACCCAATTTAGGTATAACGGGCAGAATAACGGGTGAAATAATGGGTAGAATCATAGTTTTACTAACTTGCACCCCATGATACTTTCAGTATCAATGCCACTCATTTTCTGGACGAAAATGAATGACATAAGGCTCCGCCCTCTGGCTATTTTCTTCGGCTGGACGCCTGCGAAAAAAACCATTCACCCAAGCCCGTCAAACTCACTGCGTTCGTCTGACCACCCCCTTTTTCGCCCCTGTATTTGGTCGCTCAAAAATGTGGTGGTCAGACGCTTCGCTTGACGGGGGGCTTTGCCCCCAAAACGAAACACGTTCACTGGGGTTCACTTGCCATCGCTGGGGCGTGTCATTTCGTTTCGGCTCCCCCTGTTTTTCCCTTTCTTCGCTGGACGCTCATCAGGA

TAAAAACAAAAAGCCTACCGCCCTCGTTTTACTCGGTTGGTCAAATCGCTTCTTGCCACAAAACAAGTTTGTGACTTTCAGCGATTTCGCATCGTTTTACTTGTCAGTTTGCACGATGTTAAAAACGCACTCAGAAGCCCGCAAAACCGTTCTAAACTTTCAGCGTAGATTTGGGTGTTTTATAGGGTGATAGTGTCTTAAAATCGTTTCGAGGGGCTTTAATGGGGATGAACGGGAGGTTTTTGCTAACTAACTCGCCCGTTTAACATAAAAGGGATTGTATGAATCAATTTTAGCTAGAGTTTAAGGTTGTTCAAATTAATGTACAATGATTGCACTGTATAAATAACCAGGTGTAGCATGTATGGAAAAGCACTTTATCAATGAAAAGTTTTCACGAGATCAATTTACGGGGAATAGAGTTAAAAATATTGCCTTTTCAAATTGTGATTTTTCAGGGGTTGATTTAACTGATACTGAATTTGTTGATTGTAGTTTTTACGACAGGAATAGCTTGGAAGGGTGTGATTTTAATAGAGCCAAACTAAAAAACGCTAGCTTTAAAAGCTGCGATTTATCAATGAGTAATTTTAAAAACAT

TAGCGCCTTAGGTCTTGAAATTAGTGAGTGTTTAGCTCAAGGAGCTGATTTTCGAGGGGCTAATTTTATGAATATGATAACTACAAGGTCATGGTTTTGTAGTGCTTATATAACCAAGACAAATCTTAGTTACGCTAATTTTTCTAGAGTCATATTAGAAAAGTGCGAACTGTGGGAAAATCGCTGGAATGGCACTGTGATAACTGGCGCCGTGTTTCGTGGCTCCGATCTTTCTTGTGGGGAGTTTTCATCGTTTGATTGGTCTTTGGCTGATTTTACTGG
