# Supplementary material for: Pyrosequencing of Antibiotic-Contaminated River Sediments Reveals High Levels of Resistance and Gene Transfer Elements
Source: PLoS One. 2011 Feb 16;6(2):e17038. doi: 10.1371/journal.pone.0017038 (PMC3040208; doi:10.1371/journal.pone.0017038)
Supplement: Figure S1 — Taxonomic composition of the metagenomes from the eight sampling sites. The classification was assigned based on BLAST comparison against the NCBI GenBank non-redundant protein database (nr). (PDF) [file pone.0017038.s003.pdf]

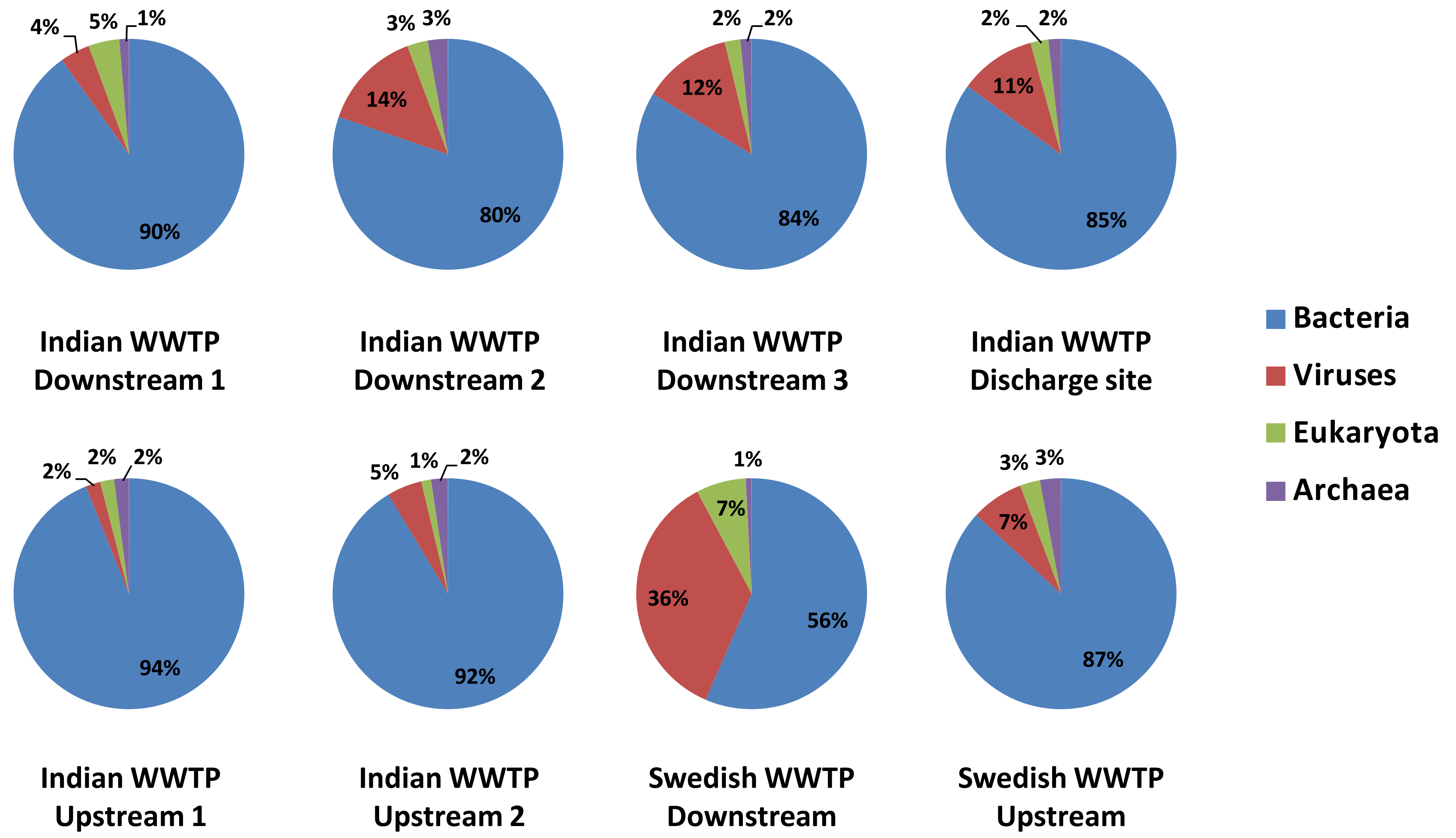
4%
5%
1%
90%
Indian WWTP
Downstream 1
3%
3%
14%
80%
Indian WWTP
Downstream 2
2%
2%
12%
84%
Indian WWTP
Downstream 3
2%
2%
11%
85%
Indian WWTP
Discharge site
Bacteria
Viruses
Eukaryota
Archaea
2%
2%
2%
94%
Indian WWTP
Upstream 1
5%
1%
2%
92%
Indian WWTP
Upstream 2
1%
7%
36%
56%
Swedish WWTP
Downstream
3%
3%
7%
87%
Swedish WWTP
Upstream
