# Supplementary figures and images for: Pyrosequencing of Antibiotic-Contaminated River Sediments Reveals High Levels of Resistance and Gene Transfer Elements
Source: PLoS One. 2011 Feb 16;6(2):e17038. doi: 10.1371/journal.pone.0017038 (PMC3040208; doi:10.1371/journal.pone.0017038)

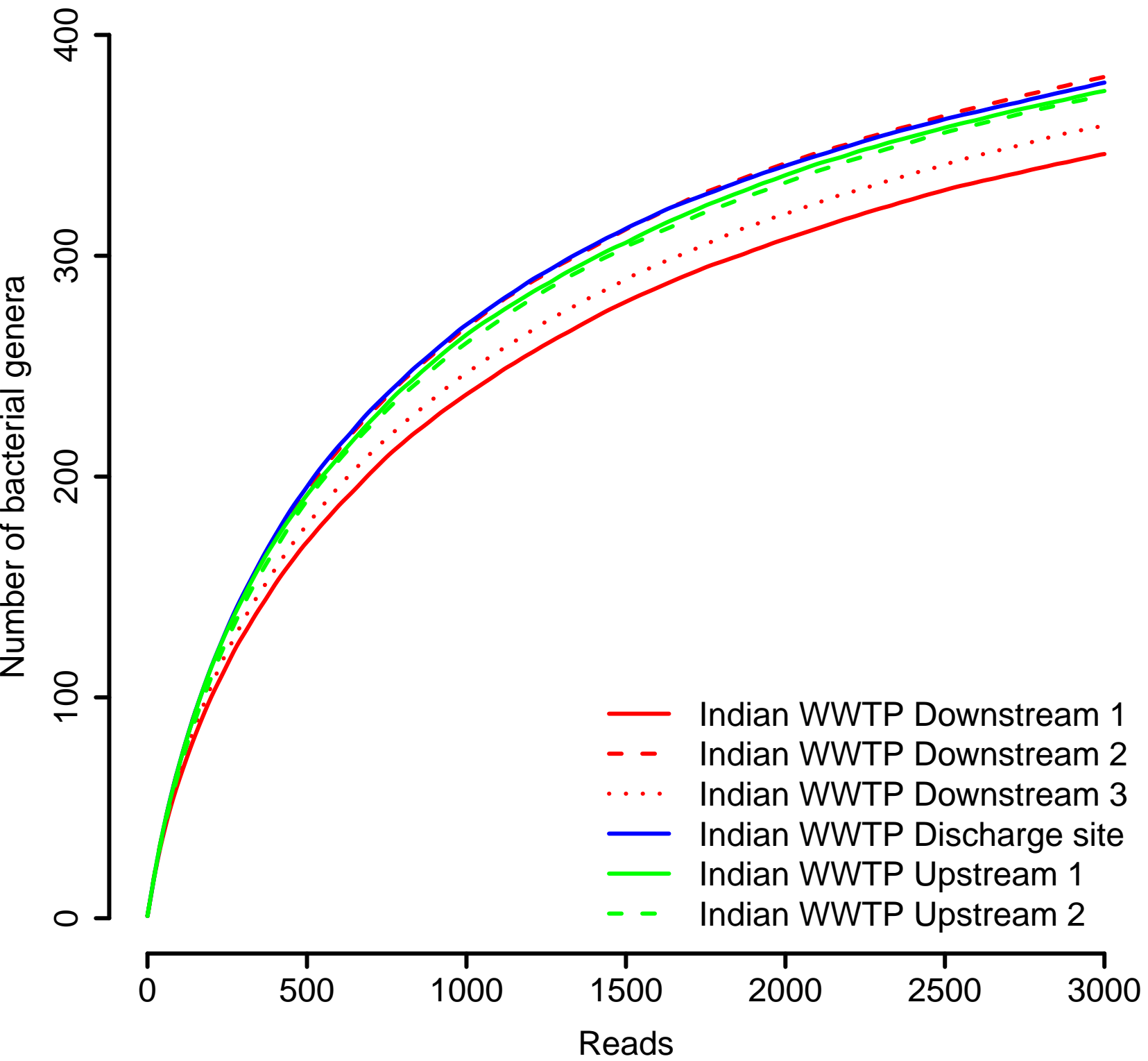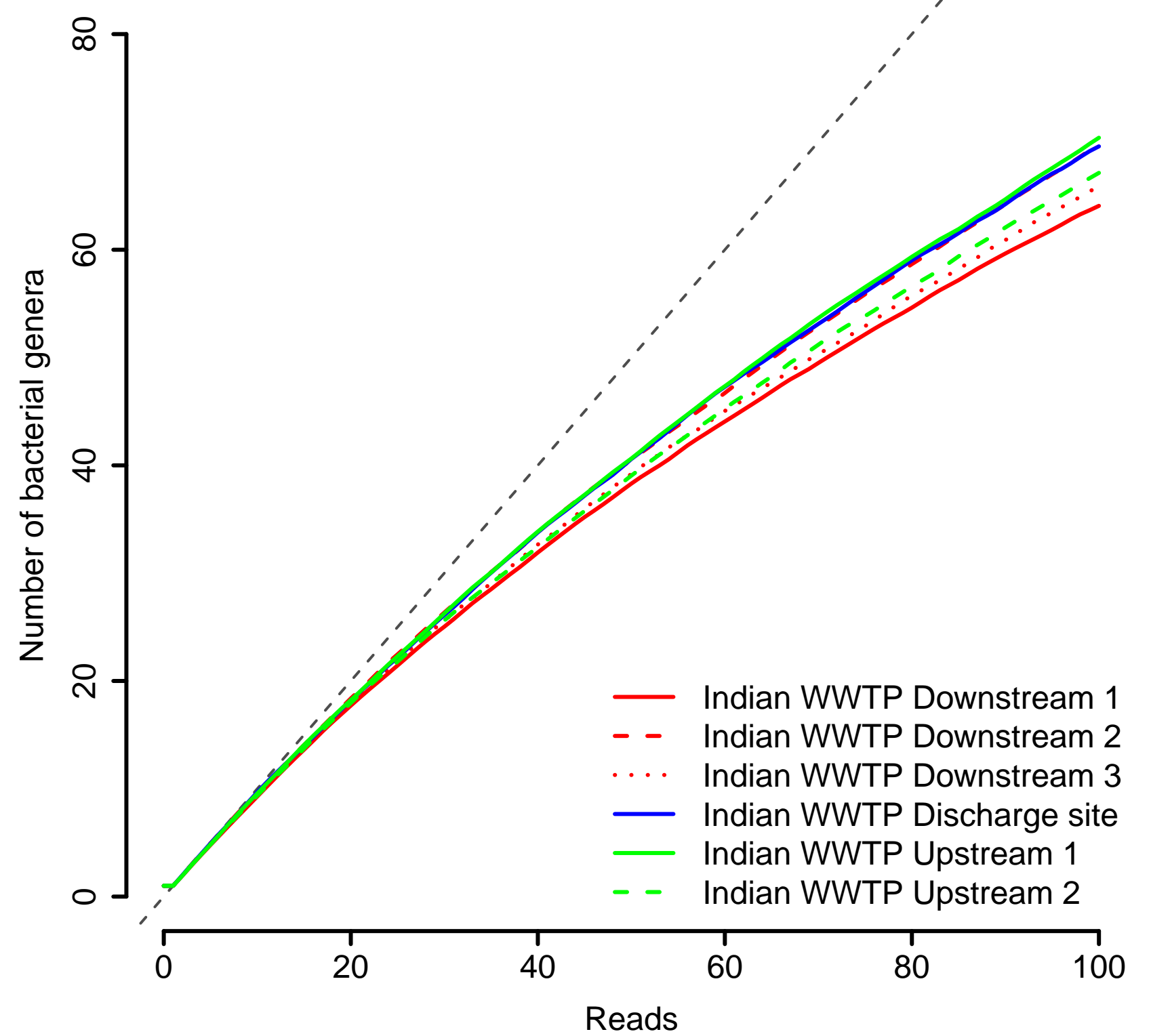

Supplement: Figure S2 — Rarefaction curves of the bacterial genera for the communities in India. Taxonomic affiliation was assigned based on BLAST comparison against the NCBI GenBank non-redundant protein database (nr). (PDF) [file pone.0017038.s004.pdf]

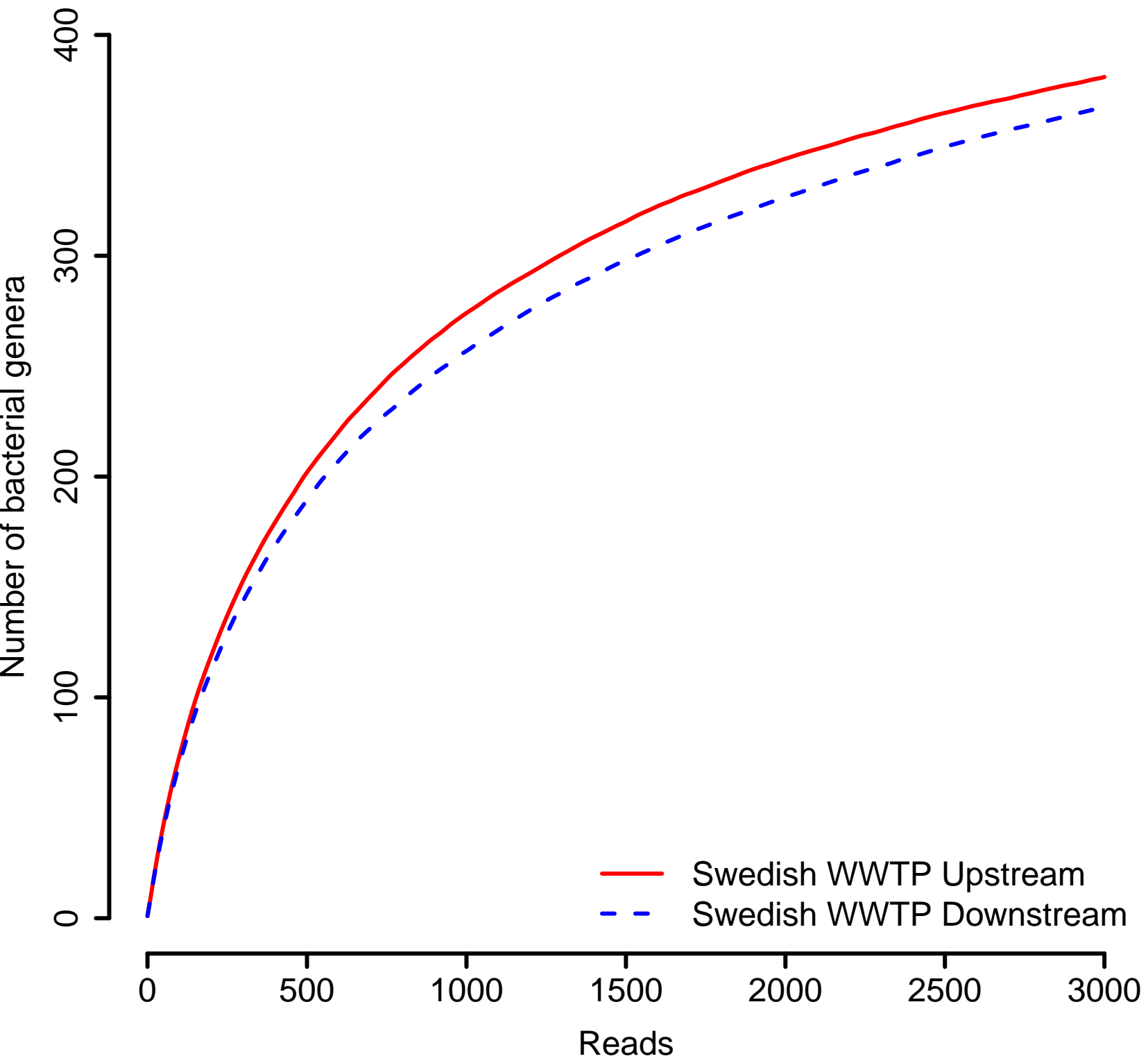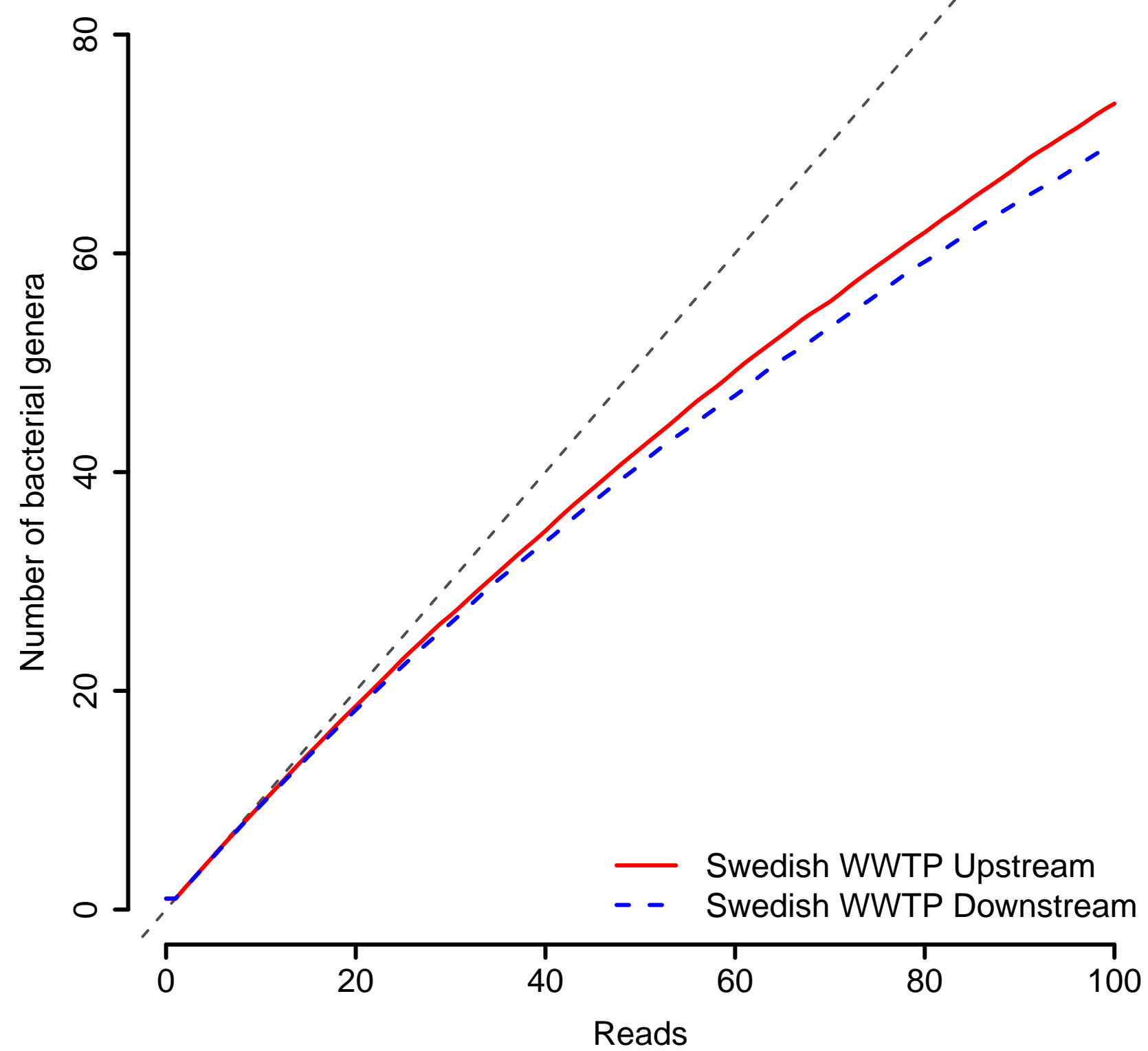

Supplement: Figure S3 — Rarefaction curves of the bacterial genera for the communities in Sweden. Taxonomic affiliation was assigned based on BLAST comparison against the NCBI GenBank non-redundant protein database (nr). (PDF) [file pone.0017038.s005.pdf]

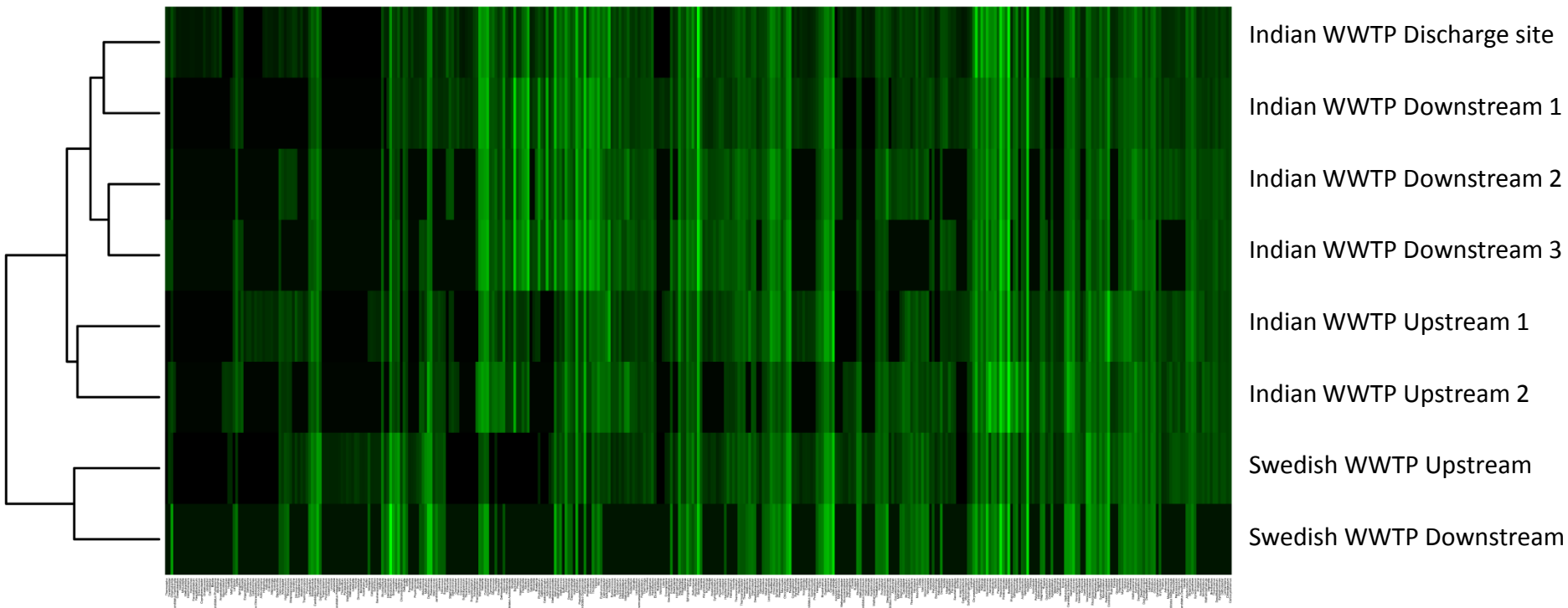

Supplement: Figure S5 — Abundance profiles for the bacterial genera in the sediment samples. The relations between the samples were calculated by hierarchical clustering using Pearson correlation distance metric. (PDF) [file pone.0017038.s007.pdf]
