# Supplementary material for: Pyrosequencing of Antibiotic-Contaminated River Sediments Reveals High Levels of Resistance and Gene Transfer Elements
Source: PLoS One. 2011 Feb 16;6(2):e17038. doi: 10.1371/journal.pone.0017038 (PMC3040208; doi:10.1371/journal.pone.0017038)
Supplement: Figure S4 — Taxonomic distribution of highly abundant bacterial phyla. Taxonomic affiliation was assigned based on BLAST comparison against the NCBI GenBank non-redundant protein database (nr). (PDF) [file pone.0017038.s006.pdf]

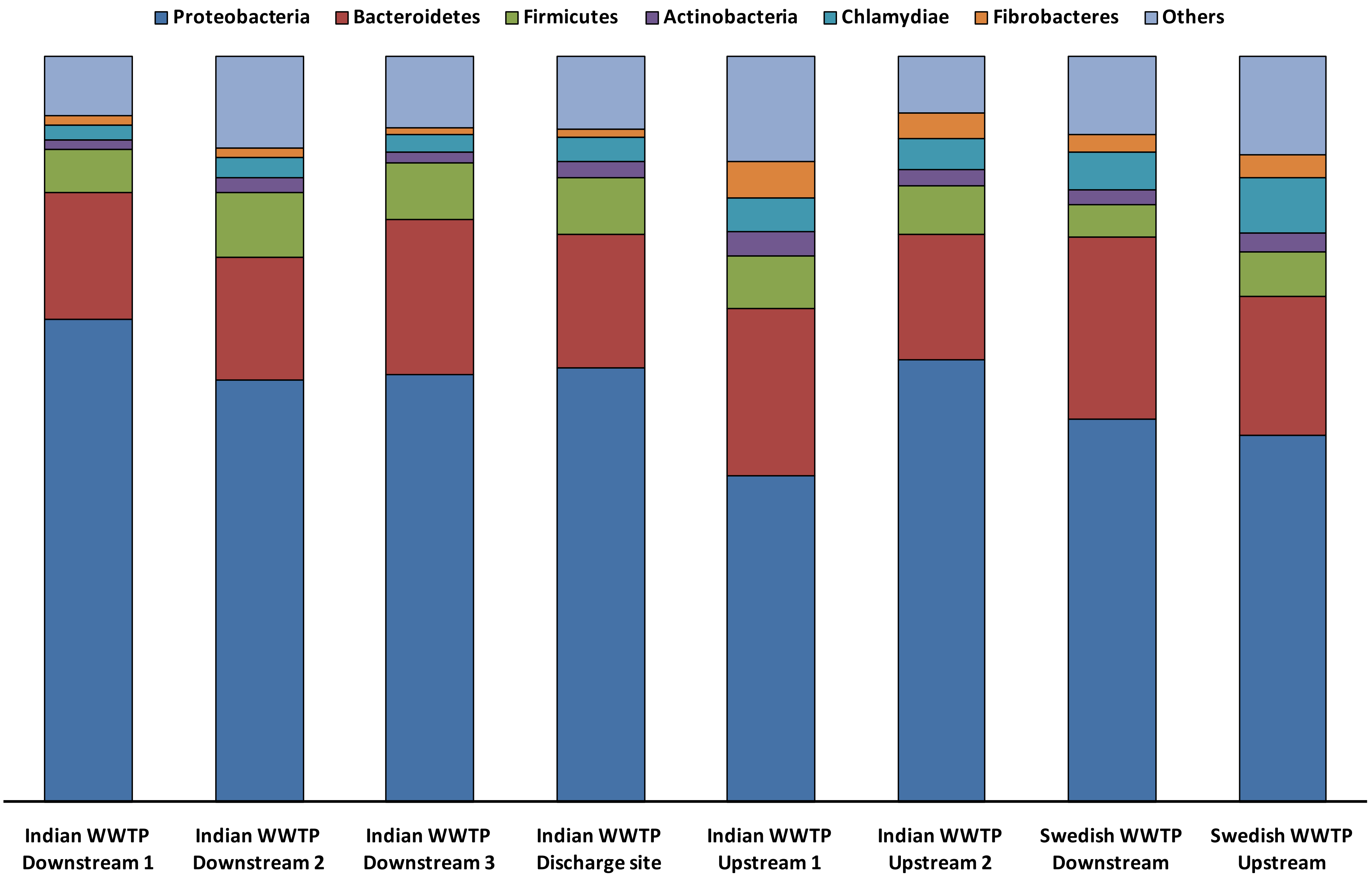
Proteobacteria
Bacteroidetes
Firmicutes
Actinobacteria
Chlamydiae
Fibrobacteres
Others
Indian WWTP Downstream 1
Indian WWTP Downstream 2
Indian WWTP Downstream 3
Indian WWTP Discharge site
Indian WWTP Upstream 1
Indian WWTP Upstream 2
Swedish WWTP Downstream
Swedish WWTP Upstream
