# Supplementary material for: Pyrosequencing of Antibiotic-Contaminated River Sediments Reveals High Levels of Resistance and Gene Transfer Elements
Source: PLoS One. 2011 Feb 16;6(2):e17038. doi: 10.1371/journal.pone.0017038 (PMC3040208; doi:10.1371/journal.pone.0017038)
Supplement: Figure S6 — PCR-verification of the novel plasmids using back-to-back outward-facing primers. As predicted, pHIRE-D1 (5174 bases) was found in the two tested downstream sites while pHIRE-U1 (2682 bases) was found in one of the upstream sites. (PDF) [file pone.0017038.s008.pdf]

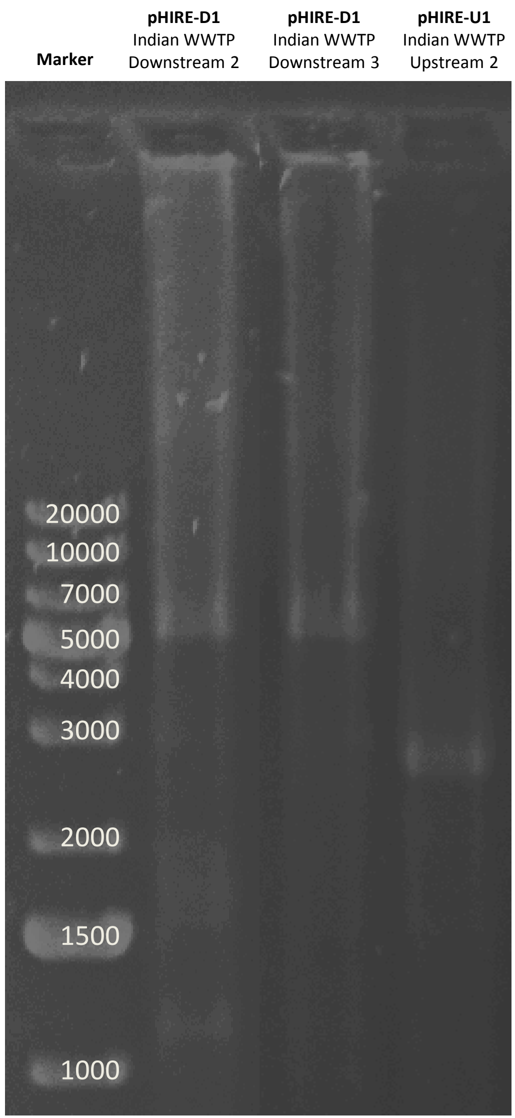
Marker
pHIRE-D1
Indian WWTP
Downstream 2
pHIRE-D1
Indian WWTP
Downstream 3
pHIRE-U1
Indian WWTP
Upstream 2
20000
10000
7000
5000
4000
3000
2000
1500
1000
