# Supplementary material for: Pyrosequencing of Antibiotic-Contaminated River Sediments Reveals High Levels of Resistance and Gene Transfer Elements
Source: PLoS One. 2011 Feb 16;6(2):e17038. doi: 10.1371/journal.pone.0017038 (PMC3040208; doi:10.1371/journal.pone.0017038)
Supplement: Table S1 — Detailed information regarding the sampling sites. (PDF) [file pone.0017038.s009.pdf]

**Table S1**

| *Site* | *ID* | *Distance from WWTP* | *Number of replicated samples* | *GPS coordinates* |
|---|---|---|---|---|
| ***Indian WWTP Downstream 1*** | *R2* | *2.3 km downstream* | *5* | N 17° 33. 077<br>E 78° 13.983 |
| ***Indian WWTP Downstream 2*** | *R3* | *2.7 km downstream* | *5* | N 17° 33. 211<br>E 78° 13.846 |
| ***Indian WWTP Downstream 3*** | *R1* | *17.5 km downstream* | *5* | N 17° 37. 945<br>E 78° 09. 813 |
| ***Indian WWTP Discharge site*** | *R4* | *50 m downstream* | *5* | N 17° 32. 394<br>E 78° 14. 552 |
| ***Indian WWTP Upstream 1*** | *R5* | *1.9 km upstream* | *5* | N 17° 37. 357<br>E 78° 15. 537 |
| ***Indian WWTP Upstream 2*** | *R6* | *2.2 km upstream* | *5* | N 17° 32. 440<br>E 78° 15. 707 |
| ***Swedish WWTP Downstream*** | *N* | *25 - 230 m downstream* | *6* | N 58° 23. 350<br>E 13° 52. 900 |
| ***Swedish WWTP Upstream*** | *U* | *5 - 100 m upstream* | *5* | N 58° 23. 500<br>E 13° 52. 700 |
