# Supplementary material for: Pyrosequencing of Antibiotic-Contaminated River Sediments Reveals High Levels of Resistance and Gene Transfer Elements
Source: PLoS One. 2011 Feb 16;6(2):e17038. doi: 10.1371/journal.pone.0017038 (PMC3040208; doi:10.1371/journal.pone.0017038)
Supplement: Table S2 — Chemical measurements of antibiotics in the river sediments. The results are given as analyte per organic weight (ng/g). The numbers in the parenthesis is the standard deviation. Substances marked with * were only measured qualitatively. (PDF) [file pone.0017038.s010.pdf]

Table S2

Substance	Detection Limit	Indian WWTP Downstream 1	Indian WWTP Downstream 2	Indian WWTP Downstream 3	Indian WWTP Discharge site	Indian WWTP Upstream 1	Indian WWTP Upstream 2	Swedish WWTP Downstream	Swedish WWTP Upstream
Fluroquinolones									
**Ciprofloxacin**	20	914044 (90758)	252783 (98639)	51987 (15629)	529215 (133837)	5244 (4244)	7149 (3070)	N.D.	N.D.
**Difloxacin**	20	N.D.	N.D.	N.D.	N.D.	1523 (1033)	N.D.	N.D	N.D
**Enoxacin**	20	N.D.	N.D.	N.D.	N.D.	N.D.	N.D.	N.D	N.D
**Enrofloxacin**	20	102865 (40407)	34503 (4347)	11452 (3743)	59646 (2222)	9422 (1438)	4608 (5349)	N.D	N.D
**Lomefloxacin**	20	N.D	N.D	N.D	N.D	3748 (3580)	844 (327)	N.D	N.D
**Ofloxacin**	20	1218 (674)	1857 (1997)	3545 (3346)	762 (187)	N.D	N.D	N.D	N.D
**Pefloxacin**	20	12386 (20647)	10958 (4528)	4719 (1899)	18111 (5846)	6000 (1438)	3977 (3696)	N.D	N.D
**Norfloxacin**	20	N.D	N.D	N.D	N.D	N.D	N.D	N.D	N.D
Sulfonamides and sulfonamide-like substances									
**Sulfamethoxazol**	1	N.D.	N.D.	N.D	N.D	N.D	N.D	N.D	N.D
**Sulfanilamide**	1	N.D.	N.D.	N.D	N.D	N.D	N.D	N.D	N.D
**Sulfapyridine**	1	N.D.	N.D.	N.D	N.D	N.D	N.D	N.D	N.D
**Sulfathiazol**	1	N.D.	N.D.	N.D	N.D	N.D	N.D	N.D	N.D
**Sulfamerazine**	1	N.D.	N.D.	N.D	N.D	N.D	N.D	N.D	N.D
**Sulfamoxol**	1	N.D.	N.D.	N.D	N.D	N.D	N.D	N.D	N.D
**Sulfamethizole**	1	N.D.	N.D.	N.D	N.D	N.D	N.D	N.D	N.D
**Sulfamethazine**	1	N.D.	N.D.	N.D	N.D	N.D	N.D	N.D	N.D
**Sulfamethoxypyridazine**	1	N.D.	N.D.	N.D	N.D	N.D	N.D	N.D	N.D
**Sulfadimethoxine**	1	N.D.	N.D.	N.D	N.D	N.D	N.D	N.D	N.D
**Sulfaphenazole**	1	N.D.	N.D.	N.D	N.D	N.D	N.D	N.D	N.D
**Sulfabenzamide***	500	N.D.	N.D.	N.D	N.D	N.D	N.D	N.D	N.D
**Sulfacarbamide***	500	N.D.	N.D.	N.D	N.D	N.D	N.D	N.D	N.D
**Sulfacetamide***	500	N.D.	N.D.	N.D	N.D	N.D	N.D	N.D	N.D
**Sulfachlorpyridazine***	500	N.D.	N.D.	N.D	N.D	N.D	N.D	N.D	N.D
**Sulfachrysoidine***	500	N.D.	N.D.	N.D	N.D	N.D	N.D	N.D	N.D
**Sulfaclozine***	500	N.D.	N.D.	N.D	N.D	N.D	N.D	N.D	N.D

**Sulfadiazine***	500	N.D.	N.D.	N.D	N.D	N.D	N.D	N.D	N.D
**Sulfadicramide***	500	N.D.	N.D.	N.D	N.D	N.D	N.D	N.D	N.D
**Sulfadimidine***	500	N.D.	N.D.	N.D	N.D	N.D	N.D	N.D	N.D
**Sulfadoxine***	500	N.D.	N.D.	N.D	N.D	N.D	N.D	N.D	N.D
**Sulfafurazole***	500	N.D.	N.D.	N.D	N.D	N.D	N.D	N.D	N.D
**Sulfaguanidine***	500	N.D.	N.D.	N.D	N.D	N.D	N.D	N.D	N.D
**Sulfamethylthiazole***	500	N.D.	N.D.	N.D	N.D	N.D	N.D	N.D	N.D
**Sulfametomidine***	500	N.D.	N.D.	N.D	N.D	N.D	N.D	N.D	N.D
**Sulfametopyrazine***	500	N.D.	N.D.	N.D	N.D	N.D	N.D	N.D	N.D
**Sulfametrole***	500	N.D.	N.D.	N.D	N.D	N.D	N.D	N.D	N.D
**Sulfamonomethoxine***	500	N.D.	N.D.	N.D	N.D	N.D	N.D	N.D	N.D
**Sulfaquinoxaline***	500	N.D.	N.D.	N.D	N.D	N.D	N.D	N.D	N.D
**Sulfathiourea***	500	N.D.	N.D.	N.D	N.D	N.D	N.D	N.D	N.D
**Sulfatroxazole***	500	N.D.	N.D.	N.D	N.D	N.D	N.D	N.D	N.D
**Sulfisomidine***	500	N.D.	N.D.	N.D	N.D	N.D	N.D	N.D	N.D
**Acetyl-Sulfadiazine***	500	N.D.	N.D.	N.D	N.D	N.D	N.D	N.D	N.D
**Acetyl-Sulfadimethoxine***	500	N.D.	N.D.	N.D	N.D	N.D	N.D	N.D	N.D
**Acetyl-Sulfamethazine***	500	N.D.	N.D.	N.D	N.D	N.D	N.D	N.D	N.D
**Acetyl-Sulfamethoxazole***	500	N.D.	N.D.	N.D	N.D	N.D	N.D	N.D	N.D
**Acetyl-Sulfathiazole***	500	N.D.	N.D.	N.D	N.D	N.D	N.D	N.D	N.D
**Pterine-sulfathiazole***	500	N.D.	N.D.	N.D	N.D	N.D	N.D	N.D	N.D
**7,8-Dihydropterine-sulfathiazole***	500	N.D.	N.D.	N.D	N.D	N.D	N.D	N.D	N.D
**4-Hydroxy-sulfathiazole***	500	N.D.	N.D.	N.D	N.D	N.D	N.D	N.D	N.D
**4-Amino-2-hydroxybenzoic acid***	500	N.D.	N.D.	N.D	N.D	N.D	N.D	N.D	N.D
**Para-Aminobenzoic acid***	500	N.D.	N.D.	N.D	N.D	N.D	N.D	N.D	N.D
**4-Acetylamino- benzenesulfonyl chloride***	500	N.D.	N.D.	N.D	N.D	N.D	N.D	N.D	N.D
**4-Acetylamino- benzenesulfonic* acid**	500	N.D.	N.D.	N.D	N.D	N.D	N.D	N.D	N.D
