# Supplementary material for: Pyrosequencing of Antibiotic-Contaminated River Sediments Reveals High Levels of Resistance and Gene Transfer Elements
Source: PLoS One. 2011 Feb 16;6(2):e17038. doi: 10.1371/journal.pone.0017038 (PMC3040208; doi:10.1371/journal.pone.0017038)
Supplement: Table S3 — Chemical measurements of antibiotics in the river sediments. The results are given as analyte per dry weight (ng/g). The numbers in the parenthesis is the standard deviation. Substances marked with * were only measured qualitatively. (PDF) [file pone.0017038.s011.pdf]

Table S3

Substance	Detection Limit	Indian WWTP Downstream 1	Indian WWTP Downstream 2	Indian WWTP Downstream 3	Indian WWTP Discharge site	Indian WWTP Upstream 1	Indian WWTP Upstream 2	Swedish WWTP Downstream	Swedish WWTP Upstream
Fluroquinolones									
**Ciprofloxacin**	20	54248 (22427)	11551 (2990)	1700 (274)	18034 (3507)	449 (397)	1077 (233)	N.D.	N.D.
**Difloxacin**	20	N.D.	N.D.	N.D	N.D	146 (351)	N.D	N.D	N.D
**Enoxacin**	20	N.D.	N.D.	N.D	N.D	N.D	N.D	N.D	N.D
**Enrofloxacin**	20	5981 (2753)	1625 (87)	374 (68)	2054 (195)	912 (458)	651 (644)	N.D	N.D
**Lomefloxacin**	20	N.D.	N.D	N.D	N.D	373 (351)	134 (98)	N.D	N.D
**Ofloxacin**	20	62 (21)	80 (76)	121 (121)	26 (5)	N.D	N.D	N.D	N.D
**Pefloxacin**	20	486 (786)	502 (129)	157 (66)	611 (123)	575 (127)	581 (437)	N.D	N.D
**Norfloxacin**	20	N.D.	N.D.	N.D	N.D	N.D	N.D	N.D	N.D
Sulfonamides and sulfonamide-like substances									
**Sulfamethoxazol**	1	N.D.	N.D.	N.D	N.D	N.D	N.D	N.D	N.D
**Sulfanilamide**	1	N.D.	N.D.	N.D	N.D	N.D	N.D	N.D	N.D
**Sulfapyridine**	1	N.D.	N.D.	N.D	N.D	N.D	N.D	N.D	N.D
**Sulfathiazol**	1	N.D.	N.D.	N.D	N.D	N.D	N.D	N.D	N.D
**Sulfamerazine**	1	N.D.	N.D.	N.D	N.D	N.D	N.D	N.D	N.D
**Sulfamoxol**	1	N.D.	N.D.	N.D	N.D	N.D	N.D	N.D	N.D
**Sulfamethizole**	1	N.D.	N.D.	N.D	N.D	N.D	N.D	N.D	N.D
**Sulfamethazine**	1	N.D.	N.D.	N.D	N.D	N.D	N.D	N.D	N.D
**Sulfamethoxypyridazine**	1	N.D.	N.D.	N.D	N.D	N.D	N.D	N.D	N.D
**Sulfadimethoxine**	1	N.D.	N.D.	N.D	N.D	N.D	N.D	N.D	N.D
**Sulfaphenazole**	1	N.D.	N.D.	N.D	N.D	N.D	N.D	N.D	N.D
**Sulfabenzamide***	20	N.D.	N.D.	N.D	N.D	N.D	N.D	N.D	N.D
**Sulfacarbamide***	20	N.D.	N.D.	N.D	N.D	N.D	N.D	N.D	N.D
**Sulfacetamide***	20	N.D.	N.D.	N.D	N.D	N.D	N.D	N.D	N.D
**Sulfachlorpyridazine***	20	N.D.	N.D.	N.D	N.D	N.D	N.D	N.D	N.D
**Sulfachrysoidine***	20	N.D.	N.D.	N.D	N.D	N.D	N.D	N.D	N.D
**Sulfaclozine***	20	N.D.	N.D.	N.D	N.D	N.D	N.D	N.D	N.D

**Sulfadiazine***	20	N.D.	N.D.	N.D	N.D	N.D	N.D	N.D	N.D
**Sulfadicramide***	20	N.D.	N.D.	N.D	N.D	N.D	N.D	N.D	N.D
**Sulfadimidine***	20	N.D.	N.D.	N.D	N.D	N.D	N.D	N.D	N.D
**Sulfadoxine***	20	N.D.	N.D.	N.D	N.D	N.D	N.D	N.D	N.D
**Sulfafurazole***	20	N.D.	N.D.	N.D	N.D	N.D	N.D	N.D	N.D
**Sulfaguanidine***	20	N.D.	N.D.	N.D	N.D	N.D	N.D	N.D	N.D
**Sulfamethylthiazole***	20	N.D.	N.D.	N.D	N.D	N.D	N.D	N.D	N.D
**Sulfametomidine***	20	N.D.	N.D.	N.D	N.D	N.D	N.D	N.D	N.D
**Sulfametopyrazine***	20	N.D.	N.D.	N.D	N.D	N.D	N.D	N.D	N.D
**Sulfametrole***	20	N.D.	N.D.	N.D	N.D	N.D	N.D	N.D	N.D
**Sulfamonomethoxine***	20	N.D.	N.D.	N.D	N.D	N.D	N.D	N.D	N.D
**Sulfaquinoxaline***	20	N.D.	N.D.	N.D	N.D	N.D	N.D	N.D	N.D
**Sulfathiourea***	20	N.D.	N.D.	N.D	N.D	N.D	N.D	N.D	N.D
**Sulfatroxazole***	20	N.D.	N.D.	N.D	N.D	N.D	N.D	N.D	N.D
**Sulfisomidine***	20	N.D.	N.D.	N.D	N.D	N.D	N.D	N.D	N.D
**Acetyl-Sulfadiazine***	20	N.D.	N.D.	N.D	N.D	N.D	N.D	N.D	N.D
**Acetyl-Sulfadimethoxine***	20	N.D.	N.D.	N.D	N.D	N.D	N.D	N.D	N.D
**Acetyl-Sulfamethazine***	20	N.D.	N.D.	N.D	N.D	N.D	N.D	N.D	N.D
**Acetyl-Sulfamethoxazole***	20	N.D.	N.D.	N.D	N.D	N.D	N.D	N.D	N.D
**Acetyl-Sulfathiazole***	20	N.D.	N.D.	N.D	N.D	N.D	N.D	N.D	N.D
**Pterine-sulfathiazole***	20	N.D.	N.D.	N.D	N.D	N.D	N.D	N.D	N.D
**7,8-Dihydropterine-sulfathiazole***	20	N.D.	N.D.	N.D	N.D	N.D	N.D	N.D	N.D
**4-Hydroxy-sulfathiazole***	20	N.D.	N.D.	N.D	N.D	N.D	N.D	N.D	N.D
**4-Amino-2-hydroxybenzoic acid***	20	N.D.	N.D.	N.D	N.D	N.D	N.D	N.D	N.D
**Para-Aminobenzoic acid***	20	N.D.	N.D.	N.D	N.D	N.D	N.D	N.D	N.D
**4-Acetylamino- benzenesulfonyl chloride***	20	N.D.	N.D.	N.D	N.D	N.D	N.D	N.D	N.D
**4-Acetylamino- benzenesulfonic* acid**	20	N.D.	N.D.	N.D	N.D	N.D	N.D	N.D	N.D
