# Supplementary material for: Pyrosequencing of Antibiotic-Contaminated River Sediments Reveals High Levels of Resistance and Gene Transfer Elements
Source: PLoS One. 2011 Feb 16;6(2):e17038. doi: 10.1371/journal.pone.0017038 (PMC3040208; doi:10.1371/journal.pone.0017038)
Supplement: Table S4 — Results from the massively parallel pyrosequencing. % is given in relation to the total number of reads. (PDF) [file pone.0017038.s012.pdf]

Table S4

Site	*Number of sequences reads*	*Total number of bases (million)*	*Average read length*	*GenBank nonredundant proteins*
Indian WWTP Downstream 1	*75,765*	*27.68*	*365*	*45.1%*
Indian WWTP Downstream 2	*44,281*	*16.16*	*365*	*40.2%*
Indian WWTP Downstream 3	*39,764*	*14.27*	*359*	*43.1%*
Indian WWTP Discharge site	*88,374*	*32.81*	*371*	*42.3%*
Indian WWTP Upstream 1	*53,045*	*19.10*	*360*	*44.9%*
Indian WWTP Upstream 2	*39,034*	*13.78*	*353*	*47.5%*
Swedish WWTP Downstream	*37,895*	*13.98*	*369*	*35.7%*
Swedish WWTP Upstream	*63,365*	*22.91*	*362*	*42.3%*
Total	*441,523*	*160.69*	*364*	*42.9%*
