# Supplementary material for: Pyrosequencing of Antibiotic-Contaminated River Sediments Reveals High Levels of Resistance and Gene Transfer Elements
Source: PLoS One. 2011 Feb 16;6(2):e17038. doi: 10.1371/journal.pone.0017038 (PMC3040208; doi:10.1371/journal.pone.0017038)
Supplement: Table S5 — Abundance of bacterial genera in the sediment around the Indian and Swedish waste water treatment plants. (PDF) [file pone.0017038.s013.pdf]

Table S5

Genus	Indian WWTP Downstream 1	Indian WWTP Downstream 2	Indian WWTP Downstream 3	Indian WWTP Discharge site	Indian WWTP Upstream 1	Indian WWTP Upstream 2	Swedish WWTP Downstream	Swedish WWTP Upstream
Abiotrophia	0	0	0	15	0	0	0	0
Acaryochloris	26	18	11	22	45	10	0	32
Acholeplasma	22	0	0	38	0	0	0	0
Acidiphilium	18	13	20	30	18	25	0	17
Acidithiobacillus	51	22	19	84	67	88	15	37
Acidobacterium	24	14	0	37	71	48	10	58
Acidothermus	0	0	0	0	0	0	0	12
Acidovorax	110	61	67	254	190	144	95	168
Aciduliprofundum	12	12	0	0	0	0	0	33
Acinetobacter	699	205	248	405	189	202	61	59
Actinobacillus	522	239	225	144	0	0	0	0
Actinosynnema	0	0	0	0	15	0	0	0
Aeromonas	78	48	24	219	115	522	17	25
Agrobacterium	18	15	19	46	44	37	21	60
Akkermansia	14	0	15	21	11	30	10	99
Alcanivorax	14	11	0	24	0	20	0	36
Algoriphagus	157	89	142	167	214	56	39	185
Alicyclobacillus	18	24	10	22	12	0	0	21
Aliivibrio	228	139	98	123	18	19	0	0
Alistipes	106	46	69	142	70	109	16	32
Alkalilimnicola	0	0	0	27	19	0	0	22
Alkaliphilus	63	43	33	106	65	39	15	37
Allochromatium	55	31	26	103	100	55	17	48
Alteromonas	0	0	0	14	15	0	0	0
Anabaena	25	13	14	19	34	14	0	34
Anaerocellum	12	14	12	21	13	0	0	19
Anaerofustis	0	0	0	0	0	0	0	0
Anaeromyxobacter	61	32	25	64	176	51	42	131
Anaerotruncus	18	0	0	14	14	0	0	0
Anoxybacillus	0	0	0	0	14	0	0	0
Aquifex	0	0	0	0	0	0	0	14
Arcanobacterium	0	0	0	35	0	0	0	0
Archaeoglobus	0	0	0	13	0	10	0	27
Arcobacter	294	32	115	427	343	68	0	45
Aromatoleum	36	26	18	66	42	46	24	89
Arthrobacter	0	14	0	21	31	10	10	21
Arthrospira	28	21	0	21	26	10	0	15
Asticcacaulis	15	0	0	17	0	14	0	20
Aurantimonas	0	0	0	11	0	0	0	14
Azoarcus	38	23	15	67	44	47	20	75
Azorhizobium	0	0	0	12	12	12	0	14
Azotobacter	19	0	0	24	17	13	0	26
Bacillus	208	107	77	291	205	240	38	120
Bacteroides	1022	490	666	1236	770	1024	169	391
Bartonella	1232	436	403	227	99	255	0	0
Bdellovibrio	38	30	16	31	41	0	16	44
Beggiatoa	34	27	20	33	42	26	16	51
Beijerinckia	0	0	0	12	17	11	0	10

**Bermanella**	0	0	0	0	0	0	0	13
**Beutenbergia**	0	0	0	14	0	0	0	14
**Bifidobacterium**	26	17	13	34	10	22	0	12
**Blastopirellula**	52	50	20	101	149	41	50	171
**Blautia**	0	0	0	17	0	0	0	0
**Bordetella**	94	49	25	172	53	39	21	76
**Brachyspira**	14	0	14	21	10	0	0	11
**Bradyrhizobium**	43	19	15	45	77	53	35	178
**Brevibacillus**	15	14	12	31	25	18	0	25
**Brevundimonas**	10	0	0	11	0	19	0	13
**Brucella**	14	0	0	18	0	0	0	26
**Burkholderia**	1263	461	637	1585	360	255	133	339
**Caldicellulosiruptor**	19	12	12	19	0	0	0	0
**Caminibacter**	0	0	0	11	0	0	0	0
**Campylobacter**	183	33	49	170	74	55	0	28
**Candidatus Amoebophilus**	12	0	0	14	15	0	0	15
**Candidatus Azobacteroides**	20	12	20	16	16	12	0	0
**Candidatus Chloracidobacterium**	0	0	0	0	19	0	0	0
**Candidatus Cloacamonas**	69	49	54	70	26	22	0	26
**Candidatus Desulforudis**	13	11	0	26	21	12	0	34
**Candidatus Hamiltonella**	0	0	0	26	0	10	0	0
**Candidatus Korarchaeum**	0	0	0	10	0	0	0	17
**Candidatus Koribacter**	82	34	31	82	255	316	49	153
**Candidatus Kuenenia**	45	39	27	70	74	27	35	114
**Candidatus Methanoregula**	17	24	0	23	16	31	0	38
**Candidatus Pelagibacter**	0	0	0	0	0	0	0	10
**Candidatus Phytoplasma**	0	0	0	23	0	0	0	0
**Candidatus Protochlamydia**	0	20	0	41	0	14	0	11
**Candidatus Solibacter**	255	105	75	217	706	187	100	475
**Capnocytophaga**	41	27	36	61	33	27	14	33
**Carboxydibrachium**	18	0	0	10	16	0	0	0
**Carboxydothermus**	30	16	14	40	30	19	0	31
**Catenulispora**	0	0	0	0	14	0	0	12
**Caulobacter**	30	18	0	30	24	62	19	56
**Cellulomonas**	0	0	0	0	11	0	0	0
**Cellvibrio**	24	0	14	21	21	31	11	34
**Cenarchaeum**	0	0	0	0	19	0	0	0
**Chelativorans**	0	0	0	20	0	0	0	0
**Chitinophaga**	609	186	192	300	847	94	184	593
**Chlamydia**	14	19	0	87	0	0	0	0
**Chlamydophila**	22	30	13	57	10	20	58	17
**Chlorobaculum**	26	11	11	22	22	15	0	26
**Chlorobium**	104	51	55	131	85	76	29	115
**Chloroflexus**	126	145	73	183	179	34	41	99
**Chloroherpeton**	51	28	38	78	54	26	28	82
**Chromobacterium**	17	0	0	17	0	0	10	31
**Chryseobacterium**	97	34	43	159	104	72	38	67
**Chthoniobacter**	95	49	50	106	200	95	66	342
**Citrobacter**	933	315	411	362	52	62	0	0
**Clostridium**	378	280	299	524	302	209	64	236
**Collinsella**	0	0	0	14	13	0	0	0
**Colwellia**	10	0	0	16	14	0	0	17

**Comamonas**	369	164	168	271	207	160	63	67
**Congregibacter**	15	0	0	14	11	0	0	22
**Coprococcus**	13	0	0	11	0	0	0	0
**Coprothermobacter**	0	0	0	10	0	0	0	0
**Corynebacterium**	11	13	0	18	0	16	0	13
**Coxiella**	0	0	0	11	0	10	0	18
**Croceibacter**	41	19	34	41	24	0	0	22
**Crocosphaera**	12	0	0	0	16	0	0	11
**Cupriavidus**	280	86	101	892	259	263	68	187
**Cyanothece**	96	49	49	135	174	73	30	102
**Cytophaga**	160	51	60	114	161	34	62	193
**Dechloromonas**	149	62	47	158	113	235	125	232
**Dehalococcoides**	49	36	33	58	36	17	17	230
**Deinococcus**	0	22	10	27	39	17	10	31
**Delftia**	32	12	12	54	20	11	16	41
**Denitrovibrio**	26	16	13	27	20	0	0	10
**Desulfatibacillum**	69	60	48	86	73	70	26	119
**Desulfitobacterium**	36	24	29	44	44	32	0	48
**Desulfobacterium**	63	50	49	91	47	48	21	72
**Desulfococcus**	66	46	49	99	44	59	20	100
**Desulfohalobium**	0	0	13	16	18	18	0	19
**Desulfonatronospira**	23	17	23	30	21	24	11	43
**Desulfotalea**	40	17	26	54	22	53	29	42
**Desulfotomaculum**	41	36	32	77	47	24	19	62
**Desulfovibrio**	137	84	78	206	130	168	28	127
**Desulfuromonas**	102	36	51	113	55	43	15	37
**Dethiobacter**	29	23	13	40	27	18	0	39
**Dethiosulfovibrio**	40	17	16	50	18	68	0	0
**Diaphorobacter**	22	21	0	55	13	27	16	22
**Dickeya**	24	0	0	22	11	12	0	12
**Dictyoglomus**	33	26	21	43	30	17	0	33
**Dinoroseobacter**	0	0	0	0	0	0	0	21
**Dokdonia**	33	14	20	45	21	15	12	25
**Dyadobacter**	240	81	129	166	294	71	89	247
**Elusimicrobium**	10	10	13	0	12	10	0	17
**Endoriftia**	0	0	0	11	11	0	0	0
**Enterobacter**	27	12	15	32	16	52	0	0
**Enterococcus**	0	0	0	10	0	0	0	0
**Erwinia**	0	0	0	13	14	15	0	13
**Erythrobacter**	42	29	16	55	42	31	14	61
**Escherichia**	819	391	327	565	258	280	28	315
**Eubacterium**	26	18	25	33	19	20	0	11
**Exiguobacterium**	0	0	0	0	10	0	0	0
**Faecalibacterium**	0	0	0	10	0	0	0	0
**Fervidobacterium**	0	0	0	11	0	0	0	0
**Fibrobacter**	10	0	14	23	18	20	0	19
**Flavobacterium**	202	86	105	281	225	70	359	388
**Francisella**	14	0	0	20	0	0	0	12
**Frankia**	15	0	0	19	41	10	0	50
**Fusobacterium**	32	16	16	39	20	17	0	18
**Gallibacterium**	48	43	15	38	0	0	0	0
**Gallionella**	54	26	23	61	28	26	42	111

**Gemmata**	72	34	22	75	163	57	29	160
**Gemmatimonas**	52	18	11	37	125	0	21	108
**Geobacillus**	62	60	40	88	63	31	12	69
**Geobacter**	988	359	284	892	526	652	210	694
**Geodermatophilus**	11	0	0	0	19	0	0	13
**Gloeobacter**	23	0	0	23	49	13	0	41
**Gluconacetobacter**	0	0	0	13	0	11	0	14
**Gluconobacter**	0	0	0	0	0	11	0	0
**Gramella**	70	55	94	119	54	19	21	56
**Haemophilus**	118	47	60	31	15	12	0	0
**Hafnia**	0	0	0	0	0	0	0	0
**Hahella**	14	10	0	26	18	14	0	38
**Haliangium**	28	13	17	39	68	14	14	70
**Haloarcula**	0	0	0	0	0	0	0	0
**Halomonas**	0	0	0	11	0	0	0	0
**Halorhodospira**	0	0	0	10	0	0	0	0
**Halothermothrix**	17	26	21	26	30	13	10	32
**Halothiobacillus**	16	0	0	20	15	12	0	14
**Helicobacter**	65	14	16	88	17	17	0	19
**Heliobacterium**	14	12	18	35	26	10	0	33
**Herminiimonas**	20	0	0	17	10	0	23	36
**Herpetosiphon**	94	93	64	138	171	34	38	89
**Hirschia**	0	0	0	0	0	0	0	23
**Histophilus**	0	0	0	14	11	0	0	0
**Hoeflea**	0	0	0	0	0	12	0	12
**Holdemania**	21	11	20	37	11	10	0	0
**Hydrogenivirga**	21	0	12	28	24	0	0	19
**Hyphomicrobium**	0	10	0	11	21	10	15	40
**Hyphomonas**	0	0	0	15	0	0	0	29
**Idiomarina**	0	13	0	30	12	0	0	10
**Janibacter**	0	0	0	12	0	0	0	0
**Jannaschia**	0	0	0	0	0	0	0	10
**Janthinobacterium**	20	0	0	28	18	18	31	46
**Klebsiella**	27	0	11	29	38	34	0	0
**Kordia**	86	35	41	94	46	28	18	70
**Kosmotoga**	16	30	17	35	16	0	0	19
**Kribbella**	0	0	0	17	13	0	0	0
**Labrenzia**	13	0	0	18	29	17	14	39
**Lactobacillus**	26	17	17	44	29	21	0	21
**Lactococcus**	14	0	0	17	0	0	0	0
**Laribacter**	18	0	10	19	22	20	12	0
**Lawsonia**	0	0	0	0	0	0	0	10
**Leeuwenhoekiella**	55	48	32	86	57	23	16	42
**Legionella**	27	16	12	32	25	31	0	45
**Lentisphaera**	31	17	17	37	24	18	0	40
**Leptospira**	38	20	32	43	38	25	16	44
**Leptospirillum**	25	24	23	47	52	24	31	67
**Leptothrix**	27	10	14	34	36	15	32	191
**Limnobacter**	0	0	0	19	13	0	13	19
**Listeria**	18	0	0	0	11	0	0	0
**Loktanella**	0	0	0	0	0	0	0	12
**Lutiella**	19	12	0	23	25	16	17	42

**Lyngbya**	39	18	11	32	48	13	12	23
**Magnetococcus**	29	17	0	22	15	20	0	21
**Magnetospirillum**	66	25	22	99	54	62	21	73
**Mannheimia**	16	0	0	0	0	0	0	0
**Maricaulis**	0	0	0	0	0	0	0	11
**Marinobacter**	35	30	26	63	49	26	24	43
**Marinomonas**	16	0	0	20	19	14	15	25
**Mariprofundus**	13	14	0	29	18	0	0	27
**Meiothermus**	32	24	19	48	60	14	15	38
**Mesorhizobium**	42	39	0	68	56	38	25	89
**Methanocaldococcus**	0	0	0	13	0	0	0	17
**Methanococcoides**	41	21	13	31	0	0	0	25
**Methanococcus**	17	12	0	16	0	0	0	27
**Methanoculleus**	20	18	0	27	0	22	0	21
**Methanohalophilus**	0	0	0	0	0	0	0	0
**Methanopyrus**	0	0	0	0	0	0	0	15
**Methanosaeta**	45	76	30	84	17	100	0	31
**Methanosarcina**	75	82	40	95	52	52	24	91
**Methanosphaerula**	18	15	10	23	18	28	0	30
**Methanospirillum**	44	42	13	46	17	80	0	20
**Methanothermobacter**	16	14	0	33	0	0	0	20
**Methylacidiphilum**	0	0	0	0	0	0	0	19
**Methylibium**	20	0	12	30	24	13	23	191
**Methylobacillus**	79	25	24	83	10	13	14	27
**Methylobacterium**	51	30	26	74	126	78	50	85
**Methylocella**	10	0	0	0	0	10	0	12
**Methylococcus**	22	14	13	45	28	52	27	115
**Methylophaga**	444	123	121	380	90	128	30	39
**Methylophilus**	0	0	0	10	0	0	0	0
**Methylotenera**	655	133	212	415	26	53	81	107
**Methylovorus**	158	45	38	130	21	18	31	52
**Micrococcus**	51	26	22	114	71	62	0	0
**Microcoleus**	40	24	18	40	62	12	12	47
**Microcystis**	34	22	15	41	35	16	16	46
**Micromonospora**	14	0	0	11	19	0	0	0
**Microscilla**	219	83	114	208	214	66	77	246
**Mitsuokella**	0	0	0	10	0	0	0	0
**Moorella**	28	23	20	48	36	10	0	56
**Moraxella**	29	0	16	16	10	0	0	0
**Mycobacterium**	34	30	16	53	70	45	21	71
**Myxococcus**	46	20	18	48	83	24	17	67
**Natranaerobius**	19	12	19	24	0	0	0	11
**Natrialba**	0	0	0	11	0	0	0	0
**Nautilia**	58	0	18	52	18	11	0	16
**Neisseria**	400	132	165	247	120	198	26	42
**Neptuniibacter**	0	0	0	16	0	13	0	13
**Nitratiruptor**	65	0	12	50	16	12	0	30
**Nitrobacter**	21	14	0	29	38	28	15	44
**Nitrococcus**	13	0	0	19	20	17	13	41
**Nitrosococcus**	23	24	0	37	38	14	23	45
**Nitrosomonas**	295	177	156	451	182	190	124	208
**Nitrosopumilus**	0	11	0	29	45	0	0	23

**Nitrosospira**	30	33	0	33	58	25	29	84
**Nitrospira**	0	0	0	0	12	0	14	41
**Nocardioides**	11	0	0	17	25	0	0	15
**Nodularia**	16	0	0	22	14	0	0	10
**Nostoc**	53	19	23	65	101	23	27	53
**Novosphingobium**	36	17	14	36	30	39	17	69
**Oceanibulbus**	0	0	0	0	0	0	0	11
**Oceanicola**	12	0	0	16	10	14	0	36
**Oceanobacillus**	0	0	0	10	0	0	0	0
**Ochrobactrum**	23	12	0	45	15	15	0	27
**Octadecabacter**	0	0	0	19	13	13	0	30
**Oligotropha**	0	0	0	16	14	11	0	16
**Opitutus**	76	41	38	90	146	58	39	169
**Oxalobacter**	19	0	0	16	0	11	0	0
**Paenibacillus**	52	32	33	86	55	27	0	60
**Parabacteroides**	264	115	156	319	171	276	49	109
**Paracoccus**	75	29	30	95	55	74	19	31
**Parvibaculum**	17	16	0	40	29	29	0	50
**Pasteurella**	302	115	125	99	15	36	0	0
**Pectobacterium**	81	28	40	72	42	49	0	21
**Pedobacter**	293	133	168	278	340	118	129	305
**Pelobacter**	301	116	166	244	101	128	41	179
**Pelodictyon**	33	18	17	30	12	27	10	30
**Pelotomaculum**	18	23	0	30	36	18	0	52
**Persephonella**	19	0	0	24	11	0	0	25
**Petrotoga**	12	12	11	23	10	0	0	13
**Phenylobacterium**	20	15	0	18	29	130	0	33
**Photobacterium**	319	111	131	52	17	0	0	11
**Photorhabdus**	114	27	42	34	19	17	0	10
**Planctomyces**	101	84	61	166	151	82	83	292
**Planococcus**	37	0	0	30	0	10	0	0
**Plesiocystis**	20	15	0	25	31	14	0	50
**Plesiomonas**	0	0	0	0	0	13	0	0
**Polaribacter**	79	40	49	57	54	18	28	61
**Polaromonas**	97	40	35	170	147	93	130	247
**Polynucleobacter**	15	0	0	22	16	10	0	12
**Porphyromonas**	75	53	48	102	63	56	15	26
**Prevotella**	47	49	36	58	36	71	11	11
**Prochlorococcus**	0	0	0	0	0	0	0	11
**Propionibacterium**	12	0	0	19	0	0	0	0
**Prosthecochloris**	18	0	0	21	19	0	0	17
**Proteus**	82	21	39	24	18	11	0	0
**Providencia**	395	121	210	125	75	20	0	10
**Pseudoalteromonas**	165	61	108	68	24	16	0	40
**Pseudomonas**	833	365	442	869	545	677	243	342
**Pseudovibrio**	25	0	0	13	19	0	0	18
**Psychrobacter**	46	17	22	29	16	0	0	14
**Psychroflexus**	59	35	39	79	34	25	21	38
**Psychromonas**	15	0	0	17	0	0	0	17
**Pyrobaculum**	0	0	0	0	0	0	0	13
**Pyrococcus**	0	0	0	12	13	0	0	29
**Ralstonia**	178	78	93	478	142	85	42	101

**Reinekea**	11	0	0	43	0	0	0	14
**Rhizobium**	92	38	43	96	101	61	25	122
**Rhodobacter**	22	13	21	52	15	46	31	121
**Rhodococcus**	0	12	0	18	28	13	19	34
**Rhodoferax**	34	15	28	77	58	27	89	261
**Rhodopirellula**	28	39	31	54	84	31	32	138
**Rhodopseudomonas**	33	21	14	46	62	44	33	112
**Rhodospirillum**	25	18	0	39	48	46	11	47
**Rhodothermus**	63	38	38	82	91	36	32	72
**Rickettsia**	10	0	0	0	0	0	0	0
**Robiginitalea**	54	25	33	60	48	18	16	31
**Roseburia**	28	30	14	33	15	0	0	0
**Roseiflexus**	163	151	92	221	277	62	31	146
**Roseobacter**	23	17	13	42	27	28	20	50
**Roseovarius**	16	0	0	23	17	16	13	52
**Rubrobacter**	26	0	0	21	83	10	0	32
**Ruegeria**	0	0	0	14	0	15	0	24
**Ruminococcus**	22	19	13	38	20	20	0	11
**Saccharophagus**	15	0	13	26	15	15	0	23
**Saccharopolyspora**	0	0	0	0	10	0	0	13
**Sagittula**	0	0	0	0	10	0	0	13
**Salinibacter**	18	13	0	38	23	0	0	13
**Salinispora**	0	0	0	0	18	0	0	0
**Salmonella**	286	122	111	171	153	75	10	0
**Sebaldella**	13	0	10	13	0	0	0	0
**Selenomonas**	26	22	0	53	15	17	0	0
**Serratia**	12	0	0	13	11	0	0	0
**Shewanella**	109	52	45	204	94	309	23	99
**Shigella**	47	14	16	28	14	15	0	0
**Sideroxydans**	35	21	14	44	32	21	72	143
**Sinorhizobium**	20	16	12	37	44	43	0	42
**Sorangium**	60	26	17	61	124	36	26	116
**Sphaerobacter**	58	69	41	98	142	21	25	77
**Sphingobacterium**	164	66	84	177	118	55	52	118
**Sphingobium**	18	0	10	30	12	23	0	0
**Sphingomonas**	82	43	22	73	111	90	16	74
**Sphingopyxis**	39	14	12	28	25	34	11	39
**Spirosoma**	234	85	121	192	301	76	103	267
**Stackebrandtia**	0	0	0	11	11	0	0	0
**Staphylococcus**	47	22	0	40	15	12	0	0
**Stenotrophomonas**	44	108	14	53	18	45	61	82
**Stigmatella**	46	23	14	46	73	26	14	78
**Streptococcus**	38	28	24	52	35	21	0	17
**Streptomyces**	52	26	22	61	91	37	16	72
**Streptosporangium**	0	12	0	21	30	10	0	25
**Subdoligranulum**	17	0	0	10	0	0	0	0
**Sulfitobacter**	14	0	19	32	13	40	0	16
**Sulfolobus**	0	0	0	0	12	0	0	13
**Sulfurihydrogenibium**	22	17	11	19	19	13	10	15
**Sulfurimonas**	296	32	60	256	35	36	11	150
**Sulfurospirillum**	120	18	22	112	106	48	0	24
**Sulfurovum**	94	12	30	84	15	30	0	35

**Symbiobacterium**	19	14	21	44	34	0	0	20
**Synechococcus**	55	40	32	63	81	52	14	71
**Synechocystis**	14	0	0	17	17	0	0	13
**Syntrophobacter**	76	72	48	104	101	67	46	152
**Syntrophomonas**	16	17	10	25	20	14	0	27
**Syntrophus**	121	107	100	191	69	144	38	176
**Teredinibacter**	0	0	12	17	0	12	0	20
**Thauera**	60	30	22	108	58	89	26	74
**Thermanaerovibrio**	28	17	13	50	0	71	0	0
**Thermoanaerobacter**	42	36	32	58	52	22	10	55
**Thermoanaerobacterium**	22	14	10	23	17	12	0	15
**Thermobaculum**	55	46	42	86	78	29	10	66
**Thermococcus**	17	30	21	36	21	11	0	70
**Thermodesulfovibrio**	10	22	0	35	31	16	19	88
**Thermofilum**	0	0	0	0	0	0	0	15
**Thermomicrobium**	24	40	23	68	62	15	0	31
**Thermomonospora**	0	0	0	10	10	0	0	15
**Thermoplasma**	0	0	0	0	0	0	0	11
**Thermosinus**	24	22	20	32	41	36	0	47
**Thermosipho**	0	0	11	24	0	0	0	0
**Thermotoga**	30	19	20	47	16	16	0	41
**Thermus**	15	12	0	21	26	10	0	14
**Thioalkalivibrio**	17	25	11	52	28	28	22	74
**Thiobacillus**	331	155	165	212	61	58	31	67
**Thiomicrospira**	11	0	0	14	0	0	0	0
**Tolumonas**	14	0	0	19	14	47	0	10
**Treponema**	32	17	16	30	17	13	0	24
**Trichodesmium**	0	0	0	13	20	0	0	11
**Variovorax**	33	14	0	52	43	19	32	123
**Verminephrobacter**	29	12	14	53	37	41	18	55
**Verrucomicrobium**	89	37	57	128	95	91	56	375
**Vibrio**	439	184	191	230	91	107	31	54
**Victivallis**	20	25	24	29	19	25	0	28
**Wolinella**	47	0	10	23	0	11	0	25
**Xanthobacter**	10	0	0	16	20	15	0	26
**Xanthomonas**	75	48	40	152	105	169	58	95
**Xylella**	54	26	36	64	70	49	23	21
**Yersinia**	169	86	85	116	55	42	12	25
**Zymomonas**	50	21	19	80	33	37	0	0
