# Supplementary material for: Pyrosequencing of Antibiotic-Contaminated River Sediments Reveals High Levels of Resistance and Gene Transfer Elements
Source: PLoS One. 2011 Feb 16;6(2):e17038. doi: 10.1371/journal.pone.0017038 (PMC3040208; doi:10.1371/journal.pone.0017038)
Supplement: Table S6 — Significant clusters of orthologous genes (COG) between sites up and downstream from the Indian treatment plant. (PDF) [file pone.0017038.s014.pdf]

Table S6

GeneFamily	Coefficient	AIC	P-value	FDR	Annotation
**COG0294**	3.41	888.22	0	0	Dihydropteroate synthase and related enzymes
**COG1109**	2.12	225.87	3.06E-55	5.89E-52	Phosphomannomutase
**COG3668**	1.41	184.85	2.00E-44	2.57E-41	Plasmid stabilization system protein
**COG3570**	2.93	208.97	5.96E-43	5.73E-40	Streptomycin 6-kinase
**COG3231**	4.02	133.44	1.70E-37	1.31E-34	Aminoglycoside phosphotransferase
**COG1357**	-2.22	39.68	8.00E-26	5.13E-23	Uncharacterized low-complexity proteins
**COG3108**	-3.49	34.56	4.31E-14	2.37E-11	Uncharacterized protein conserved in bacteria
**COG0711**	1.43	58.55	2.83E-10	1.36E-07	F0F1-type ATP synthase, subunit b
**COG0483**	1.71	184.36	3.80E-08	1.62E-05	Archaeal fructose-1,6-bisphosphatase and related enzymes of inositol monophosphatase family
**COG5616**	-2.02	32.57	8.47E-08	3.26E-05	Predicted integral membrane protein
**COG3903**	-2.05	36.50	4.16E-07	0.000146	Predicted ATPase
**COG0488**	0.64	64.95	1.83E-06	0.000588	ATPase components of ABC transporters with duplicated ATPase domains
**COG5424**	-21.79	13.86	6.00E-06	0.001777	Pyrroloquinoline quinone (Coenzyme PQQ) biosynthesis protein C
**COG2026**	-0.53	52.21	3.79E-05	0.010367	Cytotoxic translational repressor of toxin-antitoxin stability system
**COG0823**	-0.81	47.00	4.06E-05	0.010367	Periplasmic component of the Tol biopolymer transport system
**COG3905**	-1.62	28.82	4.58E-05	0.010367	Predicted transcriptional regulator
**COG0820**	0.89	46.60	4.58E-05	0.010367	Predicted Fe-S-cluster redox enzyme
**COG1961**	-0.53	52.21	5.06E-05	0.010823	Site-specific recombinases, DNA invertase Pin homologs
**COG1801**	-2.46	16.69	8.60E-05	0.017407	Uncharacterized conserved protein
**COG1228**	-1.12	34.09	9.68E-05	0.018621	Imidazolonepropionase and related amidohydrolases
**COG0155**	1.15	99.22	0.000104	0.019112	Sulfite reductase, beta subunit (hemoprotein)
**COG3119**	-0.62	45.39	0.000148	0.025806	Arylsulfatase A and related enzymes
**COG2984**	-1.93	34.48	0.00017	0.027945	ABC-type uncharacterized transport system, periplasmic

					component
**COG0372**	-1.25	29.09	0.000174	0.027945	Citrate synthase
**COG5059**	18.73	39.43	0.000192	0.029583	Kinesin-like protein
**COG5302**	-21.39	14.07	0.000219	0.031274	Post-segregation antitoxin (ccd killing mechanism protein) encoded by the F plasmid
**COG0610**	0.97	35.32	0.000219	0.031274	Type I site-specific restriction-modification system, R (restriction) subunit and related helicases
**COG1410**	-0.83	35.33	0.000232	0.031823	Methionine synthase I, cobalamin-binding domain
**COG1529**	-0.71	46.10	0.000284	0.03762	Aerobic-type carbon monoxide dehydrogenase, large subunit CoxL/CutL homologs
**COG3193**	-2.83	16.94	0.000365	0.046785	Uncharacterized protein, possibly involved in utilization of glycolate and propanediol
**COG1009**	-0.75	32.54	0.000422	0.052381	NADH:ubiquinone oxidoreductase subunit 5 (chain L)/Multisubunit Na+/H+ antiporter, MnhA subunit
**COG1917**	-1.60	33.01	0.000513	0.061628	Uncharacterized conserved protein, contains double-stranded beta-helix domain
**COG0665**	-1.11	27.16	0.000557	0.064961	Glycine/D-amino acid oxidases (deaminating)
**COG1075**	-25.20	9.61	0.000742	0.083245	Predicted acetyltransferases and hydrolases with the alpha/beta hydrolase fold
**COG2946**	-0.41	87.01	0.000757	0.083245	Putative phage replication protein RstA
**COG2602**	0.98	52.00	0.000888	0.091066	Beta-lactamase class D
**COG2352**	-1.96	19.78	0.000894	0.091066	Phosphoenolpyruvate carboxylase
**COG0002**	-1.24	25.04	0.000923	0.091066	Acetylglutamate semialdehyde dehydrogenase
**COG2141**	-1.24	26.99	0.000923	0.091066	Coenzyme F420-dependent N5,N10-methylene tetrahydromethanopterin reductase and related flavin-dependent oxidoreductases
