# Supplementary material for: Pyrosequencing of Antibiotic-Contaminated River Sediments Reveals High Levels of Resistance and Gene Transfer Elements
Source: PLoS One. 2011 Feb 16;6(2):e17038. doi: 10.1371/journal.pone.0017038 (PMC3040208; doi:10.1371/journal.pone.0017038)
Supplement: Table S7 — Significant clusters of orthologous genes (COG) between the upstream sites at the Indian treatment plant and the sites at the Swedish treatment plant. (PDF) [file pone.0017038.s015.pdf]

Table S7

GeneFamily	Coefficient	AIC	P-value	FDR	Annotation
**COG0553**	-2.34	77.17	1.11E-26	4.29E-23	Superfamily II DNA/RNA helicases, SNF2 family
**COG2026**	2.35	31.91	2.75E-22	5.28E-19	Cytotoxic translational repressor of toxin-antitoxin stability system
**COG4710**	4.12	22.08	7.28E-19	9.34E-16	Predicted DNA-binding protein with an HTH domain
**COG5281**	-20.58	36.22	1.07E-17	1.03E-14	Phage-related minor tail protein
**COG1357**	2.31	28.97	4.27E-17	3.29E-14	Uncharacterized low-complexity proteins
**COG5527**	0.97	64.27	1.50E-16	9.61E-14	Protein involved in initiation of plasmid replication
**COG0513**	-1.36	47.51	1.78E-14	9.78E-12	Superfamily II DNA and RNA helicases
**COG3668**	2.07	23.11	6.28E-14	3.02E-11	Plasmid stabilization system protein
**COG1961**	1.33	45.82	8.28E-12	3.54E-09	Site-specific recombinases, DNA invertase Pin homologs
**COG5655**	1.78	34.17	2.84E-10	1.09E-07	Plasmid rolling circle replication initiator protein and truncated derivatives
**COG2946**	1.03	49.51	1.55E-09	5.43E-07	Putative phage replication protein RstA
**COG3108**	3.35	32.18	8.67E-09	2.78E-06	Uncharacterized protein conserved in bacteria
**COG3378**	1.94	27.71	2.97E-08	8.80E-06	Predicted ATPase
**COG5534**	20.36	28.33	3.14E-07	8.63E-05	Plasmid replication initiator protein
**COG1192**	0.84	36.98	4.90E-07	0.000126	ATPases involved in chromosome partitioning
**COG0294**	1.42	21.82	3.53E-05	0.008489	Dihydropteroate synthase and related enzymes
**COG4886**	-2.79	15.75	6.89E-05	0.015582	Leucine-rich repeat (LRR) protein
**COG0532**	-1.35	21.58	7.90E-05	0.016877	Translation initiation factor 2 (IF-2; GTPase)
**COG4983**	2.17	31.49	0.000134	0.027193	Uncharacterized conserved protein
**COG1486**	21.76	13.28	0.000149	0.028596	Alpha-galactosidases/6-phospho-beta-glucosidases, family 4 of glycosyl hydrolases
**COG3041**	2.63	16.46	0.000225	0.041265	Uncharacterized protein conserved in bacteria
**COG0817**	24.66	10.93	0.000298	0.049897	Holliday junction resolvasome, endonuclease subunit
**COG1951**	23.66	11.30	0.000298	0.049897	Tartrate dehydratase alpha subunit/Fumarate hydratase class I, N-terminal domain

**COG0069**	-1.56	18.68	0.000319	0.050641	Glutamate synthase domain 2
**COG3696**	-0.57	31.02	0.000348	0.050641	Putative silver efflux pump
**COG3505**	1.82	23.84	0.000355	0.050641	Type IV secretory pathway, VirD4 components
**COG4942**	1.82	19.60	0.000355	0.050641	Membrane-bound metallopeptidase
**COG3598**	2.06	21.62	0.000433	0.058479	RecA-family ATPase
**COG0474**	-0.69	24.73	0.000441	0.058479	Cation transport ATPase
**COG3905**	1.77	22.52	0.000615	0.078811	Predicted transcriptional regulator
**COG3692**	2.49	13.98	0.000789	0.095571	Uncharacterized protein conserved in bacteria
**COG1410**	0.95	25.92	0.000795	0.095571	Methionine synthase I, cobalamin-binding domain
