# Supplementary material for: Pyrosequencing of Antibiotic-Contaminated River Sediments Reveals High Levels of Resistance and Gene Transfer Elements
Source: PLoS One. 2011 Feb 16;6(2):e17038. doi: 10.1371/journal.pone.0017038 (PMC3040208; doi:10.1371/journal.pone.0017038)
Supplement: Table S8 — Significant clusters of orthologous genes (COG) between the downstream sites at the Indian treatment plant and the sites at the Swedish treatment plant. (PDF) [file pone.0017038.s016.pdf]

Table S8

GeneFamily	Coefficient	AIC	P-value	FDR	Annotation
**COG0294**	4.83732	882.9636	0	0	Dihydropteroate synthase and related enzymes
**COG3668**	3.473867	179.9825	1.03E-90	1.98E-87	Plasmid stabilization system protein
**COG1109**	2.149108	224.3091	1.60E-52	2.05E-49	Phosphomannomutase
**COG3570**	20.37122	202.1669	6.47E-52	6.22E-49	Streptomycin 6-kinase
**COG0553**	-2.44114	86.51388	2.69E-41	2.07E-38	Superfamily II DNA/RNA helicases, SNF2 family
**COG3231**	20.073	127.2489	5.55E-39	3.56E-36	Aminoglycoside phosphotransferase
**COG5281**	-21.5854	36.21681	8.97E-28	4.93E-25	Phage-related minor tail protein
**COG5527**	1.076983	97.55947	3.66E-26	1.76E-23	Protein involved in initiation of plasmid replication
**COG5655**	2.053102	53.74112	4.73E-18	2.02E-15	Plasmid rolling circle replication initiator protein and truncated derivatives
**COG0711**	2.449213	55.00266	7.25E-16	2.79E-13	F0F1-type ATP synthase, subunit b
**COG0513**	-1.03611	66.25597	4.67E-14	1.63E-11	Superfamily II DNA and RNA helicases
**COG4710**	3.645639	33.3332	1.19E-13	3.82E-11	Predicted DNA-binding protein with an HTH domain
**COG2026**	1.828561	44.96162	2.82E-13	8.35E-11	Cytotoxic translational repressor of toxin-antitoxin stability system
**COG3328**	1.456498	46.78137	2.96E-10	8.12E-08	Transposase and inactivated derivatives
**COG1804**	-1.52485	40.40488	1.26E-09	3.24E-07	Predicted acyl-CoA transferases/carnitine dehydratase
**COG2602**	2.602289	48.50502	1.72E-08	4.14E-06	Beta-lactamase class D
**COG0820**	1.211376	47.18473	7.36E-07	0.000167	Predicted Fe-S-cluster redox enzyme
**COG3598**	2.387178	29.99889	9.86E-07	0.000201	RecA-family ATPase
**COG0243**	-1.06531	32.6891	9.91E-07	0.000201	Anaerobic dehydrogenases, typically selenocysteine-containing
**COG2352**	-2.50567	19.66826	1.08E-06	0.000209	Phosphoenolpyruvate carboxylase
**COG1593**	-1.39968	29.40679	2.26E-06	0.000414	TRAP-type C4-dicarboxylate transport system, large permease component
**COG3041**	2.857181	28.60766	2.88E-06	0.000504	Uncharacterized protein conserved in bacteria

**COG3333**	-1.79678	32.26377	3.14E-06	0.000525	Uncharacterized protein conserved in bacteria
**COG1028**	-0.5674	43.24647	4.89E-06	0.000784	Dehydrogenases with different specificities (related to short-chain alcohol dehydrogenases)
**COG3181**	-0.91174	56.93775	6.61E-06	0.001017	Uncharacterized protein conserved in bacteria
**COG2060**	-2.37214	18.95494	8.92E-06	0.001319	K+-transporting ATPase, A chain
**COG0665**	-1.35495	30.00449	1.16E-05	0.001655	Glycine/D-amino acid oxidases (deaminating)
**COG3590**	-1.99485	21.77857	1.50E-05	0.002059	Predicted metalloendopeptidase
**COG4942**	1.898331	30.48405	2.41E-05	0.003198	Membrane-bound metallopeptidase
**COG0488**	0.562759	63.00203	3.33E-05	0.004269	ATPase components of ABC transporters with duplicated ATPase domains
**COG3378**	1.45408	40.37061	3.86E-05	0.004788	Predicted ATPase
**COG4642**	19.74466	30.68517	4.56E-05	0.00548	Uncharacterized protein conserved in bacteria
**COG1961**	0.800554	45.58435	4.84E-05	0.005643	Site-specific recombinases, DNA invertase Pin homologs
**COG1008**	-1.01975	42.79718	5.36E-05	0.006066	NADH:ubiquinone oxidoreductase subunit 4 (chain M)
**COG3696**	-0.53416	42.04681	6.56E-05	0.007133	Putative silver efflux pump
**COG1883**	20.70021	22.26912	6.68E-05	0.007133	Na+-transporting methylmalonyl-CoA/oxaloacetate decarboxylase, beta subunit
**COG0069**	-1.35779	24.88907	7.38E-05	0.007528	Glutamate synthase domain 2
**COG3164**	-3.02892	14.44343	7.44E-05	0.007528	Predicted membrane protein
**COG5276**	-2.01035	21.69716	8.88E-05	0.008594	Uncharacterized conserved protein
**COG1290**	-1.42954	31.1688	8.94E-05	0.008594	Cytochrome b subunit of the bc complex
**COG1845**	-1.21736	34.319	9.42E-05	0.008636	Heme/copper-type cytochrome/quinol oxidase, subunit 3
**COG1529**	-0.77454	44.06892	9.43E-05	0.008636	Aerobic-type carbon monoxide dehydrogenase, large subunit CoxL/CutL homologs
**COG1024**	-0.85523	32.20973	1.00E-04	0.008752	Enoyl-CoA hydratase/carnithine racemase

**COG1009**	-0.8317	35.79486	0.0001	0.008752	NADH:ubiquinone oxidoreductase subunit 5 (chain L)/Multisubunit Na+/H+ antiporter, MnhA subunit
**COG0861**	-1.25914	33.95779	0.000123	0.010545	Membrane protein TerC, possibly involved in tellurium resistance
**COG0483**	1.010072	187.6465	0.000145	0.012098	Archaeal fructose-1,6-bisphosphatase and related enzymes of inositol monophosphatase family
**COG4941**	-22.4651	12.00655	0.000154	0.012643	Predicted RNA polymerase sigma factor containing a TPR repeat domain
**COG2946**	0.621086	83.26417	0.000172	0.013793	Putative phage replication protein RstA
**COG0085**	-0.85837	32.44095	0.000244	0.01916	DNA-directed RNA polymerase, beta subunit/140 kD subunit
**COG1192**	0.570769	43.12763	0.000251	0.019306	ATPases involved in chromosome partitioning
**COG4625**	-1.59384	27.53733	0.000261	0.019323	Uncharacterized protein with a C-terminal OMP (outer membrane protein) domain
**COG1734**	-1.59384	27.21696	0.000261	0.019323	DnaK suppressor protein
**COG0265**	-0.76716	33.48066	0.000274	0.01988	Trypsin-like serine proteases, typically periplasmic, contain C-terminal PDZ domain
**COG1149**	21.49954	18.23877	0.00031	0.021288	MinD superfamily P-loop ATPase containing an inserted ferredoxin domain
**COG2855**	21.49954	18.92465	0.00031	0.021288	Predicted membrane protein
**COG5059**	18.49954	39.42822	0.00031	0.021288	Kinesin-like protein
**COG0043**	-1.26248	24.52076	0.000334	0.022565	3-polyprenyl-4-hydroxybenzoate decarboxylase and related decarboxylases
**COG0304**	-0.7306	36.47608	0.000357	0.023709	3-oxoacyl-(acyl-carrier-protein) synthase
**COG4983**	1.94089	30.97676	0.000368	0.023998	Uncharacterized conserved protein
**COG1419**	-2.33578	18.22497	0.000409	0.026234	Flagellar GTP-binding protein
**COG0863**	1.055371	30.7231	0.000434	0.02739	DNA modification methylase
**COG5534**	20.44238	24.16563	0.000456	0.028118	Plasmid replication initiator protein

**COG4948**	-0.93706	31.06455	0.000468	0.028118	L-alanine-DL-glutamate epimerase and related enzymes of enolase superfamily
**COG0583**	-0.67207	38.26443	0.000469	0.028118	Transcriptional regulator
**COG4974**	0.482378	62.81666	0.000475	0.028118	Site-specific recombinase XerD
**COG1122**	2.387178	28.50933	0.000538	0.031236	ABC-type cobalt transport system, ATPase component
**COG1231**	-21.2828	10.12696	0.000552	0.031236	Monoamine oxidase
**COG1462**	-20.2828	16.46142	0.000552	0.031236	Uncharacterized protein involved in formation of curli polymers
**COG1005**	-0.86247	38.60624	0.000599	0.03342	NADH:ubiquinone oxidoreductase subunit 1 (chain H)
**COG5616**	-1.52485	24.80144	0.000614	0.033764	Predicted integral membrane protein
**COG4805**	-1.60489	22.72097	0.000639	0.034293	Uncharacterized protein conserved in bacteria
**COG4972**	-1.70717	27.08625	0.000642	0.034293	Tfp pilus assembly protein, ATPase PilM
**COG0460**	-1.21119	27.61382	0.000695	0.036614	Homoserine dehydrogenase
**COG1007**	-0.8317	34.30341	0.000721	0.037464	NADH:ubiquinone oxidoreductase subunit 2 (chain N)
**COG1639**	2.346356	21.16203	0.000761	0.039009	Predicted signal transduction protein
**COG0365**	-0.68712	35.41986	0.000913	0.046227	Acyl-coenzyme A synthetases/AMP-(fatty) acid ligases
**COG0035**	21.31722	18.6919	0.000993	0.048333	Uracil phosphoribosyltransferase
**COG0817**	21.31722	17.28319	0.000993	0.048333	Holliday junction resolvasome, endonuclease subunit
**COG1951**	20.31722	21.02497	0.000993	0.048333	Tartrate dehydratase alpha subunit/Fumarate hydratase class I, N-terminal domain
**COG0859**	-1.11938	27.52567	0.001037	0.049848	ADP-heptose:LPS heptosyltransferase
**COG1574**	-0.95953	28.87123	0.001137	0.053886	Predicted metal-dependent hydrolase with the TIM-barrel fold
**COG1032**	-0.75374	30.27511	0.001149	0.053886	Fe-S oxidoreductase
**COG0183**	-0.69817	35.13863	0.001165	0.053886	Acetyl-CoA acetyltransferase

**COG3320**	-2.21799	17.23922	0.001177	0.053886	Putative dehydrogenase domain of multifunctional non-ribosomal peptide synthetases and related enzymes
**COG2337**	1.296533	30.78524	0.001239	0.056069	Growth inhibitor
**COG0602**	-1.93031	20.16251	0.001445	0.064632	Organic radical activating enzymes
**COG4969**	2.259344	38.46698	0.001515	0.067	Tfp pilus assembly protein, major pilin PilA
**COG1999**	-1.62016	20.44168	0.001577	0.068152	Uncharacterized protein SCO1/SenC/PrrC, involved in biogenesis of respiratory and photosynthetic systems
**COG2079**	-1.62016	21.47354	0.001577	0.068152	Uncharacterized protein involved in propionate catabolism
**COG1138**	-1.18	25.17271	0.001651	0.070584	Cytochrome c biogenesis factor
**COG1960**	-0.49523	35.77116	0.001891	0.077146	Acyl-CoA dehydrogenases
**COG0412**	-1.20639	27.11187	0.001914	0.077146	Dienelactone hydrolase and related enzymes
**COG1858**	-0.9783	32.05088	0.001936	0.077146	Cytochrome c peroxidase
**COG2156**	-25.0597	8.998584	0.002005	0.077146	K+-transporting ATPase, c chain
**COG3910**	-25.0597	8.998584	0.002005	0.077146	Predicted ATPase
**COG2047**	-22.0597	9.382839	0.002005	0.077146	Uncharacterized protein (ATP-grasp superfamily)
**COG3603**	-22.0597	9.382839	0.002005	0.077146	Uncharacterized conserved protein
**COG4648**	-22.0597	9.382839	0.002005	0.077146	Predicted membrane protein
**COG5257**	-22.0597	9.382839	0.002005	0.077146	Translation initiation factor 2, gamma subunit (eIF-2gamma; GTPase)
**COG1848**	-21.0597	13.77525	0.002005	0.077146	Predicted nucleic acid-binding protein, contains PIN domain
**COG3712**	2.212824	23.40338	0.002137	0.081386	Fe2+-dicitrate sensor, membrane component
**COG2814**	-0.64165	39.83976	0.002237	0.084027	Arabinose efflux permease
**COG3889**	-0.72348	29.75045	0.00225	0.084027	Predicted solute binding protein
**COG2761**	-2.62346	15.23113	0.002294	0.084519	Predicted dithiol-disulfide isomerase involved in polyketide biosynthesis
**COG0535**	-0.98585	28.19528	0.002311	0.084519	Predicted Fe-S

					oxidoreductases
**COG0834**	1.503677	24.35363	0.002329	0.084519	ABC-type amino acid transport/signal transduction systems, periplasmic component/domain
**COG0372**	-1.08301	28.51916	0.002408	0.086568	Citrate synthase
