# Supplementary material for: Pyrosequencing of Antibiotic-Contaminated River Sediments Reveals High Levels of Resistance and Gene Transfer Elements
Source: PLoS One. 2011 Feb 16;6(2):e17038. doi: 10.1371/journal.pone.0017038 (PMC3040208; doi:10.1371/journal.pone.0017038)
Supplement: Table S9 — Significant TIGRFAM gene families between the sites up and downstream from the Indian treatment plant. (PDF) [file pone.0017038.s017.pdf]

Table S9

GeneFamily	Coefficient	AIC	P-value	FDR	Annotation
**TIGR01496**	-3.80	64.47	0	0	dihydropteroate synthase
**TIGR01455**	-2.85	37.16	4.10E-94	5.89E-91	phosphoglucosamine mutase
**TIGR01629**	1.20	140.64	6.87E-30	6.57E-27	phage/plasmid replication protein, gene II/X family
**TIGR03434**	1.04	64.56	1.15E-11	8.26E-09	Acidobacterial duplicated orphan permease
**TIGR01642**	1.04	35.96	4.88E-10	2.80E-07	U2 snRNP auxilliary factor, large subunit, splicing factor
**TIGR02794**	0.80	48.41	1.31E-07	6.28E-05	protein TolA
**TIGR02763**	-0.74	80.93	7.20E-07	0.000295	scaffolding protein
**TIGR02416**	21.86	13.50	1.29E-05	0.004627	carbon-monoxide dehydrogenase, large subunit
**TIGR02082**	0.99	28.69	2.99E-05	0.009437	methionine synthase
**TIGR00048**	-1.01	42.28	3.29E-05	0.009437	radical SAM enzyme, Cfr family
**TIGR03436**	2.05	28.49	7.14E-05	0.018616	VWFA-related Acidobacterial domain
**TIGR01652**	-19.92	21.58	9.13E-05	0.021837	phospholipid-translocating P-type ATPase, flippase
**TIGR01622**	1.01	25.02	0.000414	0.091362	splicing factor, CC1-like family
**TIGR02294**	1.23	29.57	0.000454	0.093133	nickel ABC transporter, periplasmic nickel-binding protein
**TIGR03619**	21.41	12.18	0.000502	0.093861	probable F420-dependent oxidoreductase, Rv2161c family
**TIGR02866**	1.70	24.37	0.000523	0.093861	cytochrome c oxidase, subunit II
**TIGR00348**	-1.03	27.08	0.000557	0.09407	type I site-specific deoxyribonuclease, HsdR family
