# Supplementary material for: Pyrosequencing of Antibiotic-Contaminated River Sediments Reveals High Levels of Resistance and Gene Transfer Elements
Source: PLoS One. 2011 Feb 16;6(2):e17038. doi: 10.1371/journal.pone.0017038 (PMC3040208; doi:10.1371/journal.pone.0017038)
Supplement: Table S10 — Significant TIFRFAM gene families between the Indian upstream sites and the sites up and downstream from the Swedish treatment plant. (PDF) [file pone.0017038.s018.pdf]

Table S10

GeneFamily	Coefficient	AIC	P-value	FDR	Annotation
**TIGR01629**	2.07	134.70	2.26E-46	6.50E-43	phage/plasmid replication protein. gene II/X family
**TIGR02763**	-1.53	439.16	2.51E-31	3.60E-28	scaffolding protein
**TIGR01541**	-20.65	40.76	7.19E-19	6.87E-16	phage tail tape measure protein. lambda family
**TIGR02794**	1.40	46.08	5.11E-13	3.66E-10	protein TolA
**TIGR01389**	-1.48	41.54	6.10E-12	3.50E-09	ATP-dependent DNA helicase RecQ
**TIGR01613**	2.01	27.02	4.86E-09	2.32E-06	phage/plasmid primase. P4 family. C-terminal domain
**TIGR02768**	0.77	87.62	9.43E-07	0.000386699	Ti-type conjugative transfer relaxase TraA
**TIGR03453**	1.27	23.12	3.30E-06	0.001184891	plasmid partitioning protein RepA
**TIGR02385**	2.44	26.75	4.64E-06	0.001374351	addiction module toxin. RelE/StbE family
**TIGR01496**	1.54	21.28	4.79E-06	0.001374351	dihydropteroate synthase
**TIGR02419**	-21.13	15.08	3.41E-05	0.008897982	phage/conjugal plasmid C-4 type zinc finger protein. TraR family
**TIGR03434**	0.59	57.66	8.63E-05	0.019943037	Acidobacterial duplicated orphan permease
**TIGR02668**	-2.75	17.56	9.04E-05	0.019943037	probable molybdenum cofactor biosynthesis protein A
**TIGR02680**	0.93	23.33	0.000178465	0.034786634	conserved hypothetical protein TIGR02680
**TIGR00487**	-1.24	22.28	0.000181875	0.034786634	translation initiation factor IF-2
**TIGR02601**	-1.47	21.75	0.000255605	0.045833256	autotransporter-associated beta strand repeat
**TIGR02168**	0.84	23.06	0.000300892	0.05077996	chromosome segregation protein SMC
**TIGR02745**	-2.61	16.39	0.0003575	0.056981548	cytochrome c oxidase accessory protein CcoG
**TIGR02188**	-1.00	21.13	0.000493602	0.073031938	acetate--CoA ligase
**TIGR00228**	24.61	10.77	0.000509111	0.073031938	crossover junction endodeoxyribonuclease RuvC
