# Supplementary material for: Pyrosequencing of Antibiotic-Contaminated River Sediments Reveals High Levels of Resistance and Gene Transfer Elements
Source: PLoS One. 2011 Feb 16;6(2):e17038. doi: 10.1371/journal.pone.0017038 (PMC3040208; doi:10.1371/journal.pone.0017038)
Supplement: Table S11 — Significant TIGRFAM gene families between the Indian downstream sites and the sites up and downstream from the Swedish treatment plant. (PDF) [file pone.0017038.s019.pdf]

Table S11

GeneFamily	Coefficient	AIC	P-value	FDR	Annotation
**TIGR01496**	5.34	59.73	0	0	dihydropteroate synthase
**TIGR01455**	2.95	36.03	2.71E-93	3.88E-90	phosphoglucosamine mutase
**TIGR01541**	-21.52	40.76	4.24E-23	4.05E-20	phage tail tape measure protein, lambda family
**TIGR02763**	-0.79	429.60	4.96E-15	3.56E-12	scaffolding protein
**TIGR01642**	-1.19	42.75	2.70E-13	1.55E-10	U2 snRNP auxilliary factor, large subunit, splicing factor
**TIGR01389**	-1.34	51.80	1.68E-12	8.04E-10	ATP-dependent DNA helicase RecQ
**TIGR02768**	0.95	54.19	1.73E-11	7.09E-09	Ti-type conjugative transfer relaxase TraA
**TIGR02168**	1.29	29.60	4.77E-11	1.71E-08	chromosome segregation protein SMC
**TIGR00048**	1.29	42.98	1.16E-06	0.00037	radical SAM enzyme, Cfr family
**TIGR02601**	-1.97	24.57	1.81E-06	0.00052	autotransporter-associated beta strand repeat
**TIGR01629**	0.87	35.57	2.56E-06	0.000626	phage/plasmid replication protein, gene II/X family
**TIGR03253**	-1.60	31.83	2.62E-06	0.000626	formyl-CoA transferase
**TIGR02419**	-21.00	15.08	3.77E-06	0.000833	phage/conjugal plasmid C-4 type zinc finger protein, TraR family
**TIGR00786**	-1.40	27.81	6.15E-06	0.001261	TRAP transporter, DctM subunit
**TIGR03194**	-1.53	26.28	9.72E-06	0.00186	4-hydroxybenzoyl-CoA reductase, alpha subunit
**TIGR02037**	-0.98	27.61	1.05E-05	0.001882	protease Do
**TIGR00601**	-1.06	33.19	1.17E-05	0.001982	UV excision repair protein Rad23
**TIGR02385**	2.28	27.63	1.74E-05	0.00278	addiction module toxin, RelE/StbE family
**TIGR01613**	1.48	36.32	7.09E-05	0.01071	phage/plasmid primase, P4 family, C-terminal domain
**TIGR02866**	-1.87	26.61	8.57E-05	0.012292	cytochrome c oxidase, subunit II
**TIGR03436**	-2.04	18.04	0.000102	0.013951	VWFA-related Acidobacterial domain
**TIGR01972**	-0.91	29.89	0.000152	0.019126	proton-translocating NADH-quinone oxidoreductase, chain M
**TIGR02416**	-22.59	10.53	0.00016	0.019126	carbon-monoxide dehydrogenase, large subunit
**TIGR02621**	-20.59	12.43	0.00016	0.019126	CRISPR-associated helicase Cas3, Anaes-subtype
**TIGR02099**	-2.76	15.55	0.000207	0.023226	conserved hypothetical protein TIGR02099
**TIGR01109**	20.66	17.85	0.00021	0.023226	sodium ion-translocating decarboxylase, beta subunit
**TIGR00680**	-2.23	16.77	0.00024	0.025203	K+-transporting ATPase, A subunit

**TIGR03453**	1.01	27.85	0.000246	0.025203	plasmid partitioning protein RepA
**TIGR01830**	-0.54	35.07	0.000295	0.029205	3-oxoacyl-[acyl-carrier-protein] reductase
**TIGR03365**	-22.46	11.38	0.000414	0.039559	7-cyano-7-deazaguanosine (preQ0) biosynthesis protein QueE
**TIGR03339**	-1.41	22.45	0.000499	0.046174	aminoethylphosphonate catabolism associated LysR family transcriptional regulator
**TIGR02169**	0.92	29.30	0.000689	0.061764	chromosome segregation protein SMC
**TIGR02668**	-1.68	21.16	0.000739	0.064208	probable molybdenum cofactor biosynthesis protein A
**TIGR01770**	-0.88	31.64	0.00081	0.066386	proton-translocating NADH-quinone oxidoreductase, chain N
**TIGR03207**	-0.88	27.71	0.00081	0.066386	cyclohexanecarboxyl-CoA dehydrogenase
**TIGR01127**	2.41	17.26	0.000873	0.068709	threonine dehydratase
**TIGR02013**	-0.83	27.67	0.000886	0.068709	DNA-directed RNA polymerase, beta subunit
**TIGR02224**	0.66	45.52	0.00102	0.075942	tyrosine recombinase XerC
**TIGR01571**	-23.30	10.12	0.001078	0.075942	uncharacterized Cys-rich domain
**TIGR02435**	-23.30	10.12	0.001078	0.075942	precorrin-3B synthase
**TIGR01085**	1.28	24.51	0.001106	0.075942	UDP-N-acetylmuramyl-tripeptide synthetases
**TIGR03329**	-2.56	18.10	0.001139	0.075942	putative aminophosphonate oxidoreductase
**TIGR01845**	-1.10	27.16	0.001139	0.075942	efflux transporter, outer membrane factor (OMF) lipoprotein, NodT family
**TIGR01929**	-2.07	21.18	0.001165	0.075942	naphthoate synthase
**TIGR01974**	-0.74	32.89	0.001444	0.09204	proton-translocating NADH-quinone oxidoreductase, chain L
