# Supplementary material for: Pyrosequencing of Antibiotic-Contaminated River Sediments Reveals High Levels of Resistance and Gene Transfer Elements
Source: PLoS One. 2011 Feb 16;6(2):e17038. doi: 10.1371/journal.pone.0017038 (PMC3040208; doi:10.1371/journal.pone.0017038)
Supplement: Table S12 — Resistance genes identified in the metagenomes. (PDF) [file pone.0017038.s020.pdf]

Table S12

ID	Indian WWTP Downstream 1	Indian WWTP Downstream 2	Indian WWTP Downstream 3	Indian WWTP Discharge site	Indian WWT Upstream 1	Indian WWTP Upstream 2	Swedish WWTP Downstream	Swedish WWTP Upstream	Total	Gene	Resistance
**ARGENE000142**	1119	490	489	129	9	12	0	0	2248	sul2	Sulfonamide
**ARGENE000144**	185	66	78	8	3	7	0	0	347	aph6id	Streptomycin
**ARGENE000034**	139	47	58	9	0	3	0	0	256	aph33ib	Streptomycin
**ARGENE000272**	15	3	8	12	24	12	6	25	105	Baca	Bacitracin
**ARGENE200007**	1	1	0	1	44	1	0	0	48	qnrD	Fluoroquinolone
**ARGENE200006**	0	0	1	0	21	13	0	0	35	qnrVC	Fluoroquinolone
**ARGENE000026**	1	0	1	3	3	5	0	0	13	bl1_cmy2	Beta-lactam class C
**ARGENE000107**	0	0	0	1	3	9	0	0	13	bl2_ges	Beta-lactam class A
**ARGENE200008**	0	0	0	2	5	6	0	0	13	qnrS	Fluoroquinolone
**ARGENE000244**	0	0	0	2	5	3	0	0	10	ant3ia	Aminoglycosides
**ARGENE000043**	7	0	1	0	0	1	0	0	9	dfra15	Trimethoprim
**ARGENE000141**	0	2	2	2	0	0	0	0	6	sul1	Sulfonamide
**ARGENE000134**	0	0	0	0	0	0	0	6	6	aac3iia	Aminoglycosides
**ARGENE000220**	2	0	0	0	4	0	0	0	6	tsnr	Thiostrepton
**ARGENE000171**	0	0	1	1	0	1	0	0	3	aac6ib	Aminoglycosides
**ARGENE000321**	1	0	0	0	0	2	0	0	3	acrb	Multidrug efflux pump
**ARGENE000376**	1	0	0	0	0	1	0	0	2	tetc	Tetracycline
**ARGENE000164**	0	1	0	0	1	0	0	0	2	bl3_shw	Beta-lactam class B
**ARGENE000375**	0	0	0	0	2	0	0	0	2	teta	Tetracycline
**ARGENE000343**	0	0	0	0	1	1	0	0	2	ceob	Chloramphenicol
**ARGENE000276**	0	0	0	1	0	0	0	0	1	ermf	Macrolide
**ARGENE000274**	0	0	1	0	0	0	0	0	1	ermc	Macrolide
**ARGENE000266**	1	0	0	0	0	0	0	0	1	mexb	Multidrug efflux pump
**ARGENE000259**	0	0	0	1	0	0	0	0	1	mexw	Multidrug efflux pump
**ARGENE000252**	1	0	0	0	0	0	0	0	1	mpha	Macrolide
**ARGENE000185**	0	0	0	1	0	0	0	0	1	bl2c_pse1	Beta-lactam class A
**ARGENE000166**	0	1	0	0	0	0	0	0	1	ant2ia	Aminoglycosides
**ARGENE000153**	0	1	0	0	0	0	0	0	1	bl2d_lcr1	Beta-lactam class D
**ARGENE000081**	0	1	0	0	0	0	0	0	1	bl2d_oxa2	Beta-lactamase class D
**ARGENE000361**	0	0	0	0	0	1	0	0	1	tetq	Tetracycline
**ARGENE000339**	0	0	0	0	1	0	0	0	1	arna	Polymyxin
**ARGENE000316**	0	0	0	0	1	0	0	0	1	macb	Macrolide

**ARGENE000262**	0	0	0	0	1	0	0	0	1	mexi	Multidrug efflux pump
**ARGENE000187**	0	0	0	0	0	1	0	0	1	erea	Macrolide
**ARGENE000182**	0	0	0	0	0	1	0	0	1	bl1_sm	Beta-lactamase class C
**ARGENE000158**	0	0	0	0	0	1	0	0	1	dfrb1	Trimethoprim
**ARGENE000115**	0	0	0	0	0	1	0	0	1	catb3	Chloramphenicol
**Total**	1473	613	640	172	128	79	6	31	3146		
