# Supplementary material for: Pyrosequencing of Antibiotic-Contaminated River Sediments Reveals High Levels of Resistance and Gene Transfer Elements
Source: PLoS One. 2011 Feb 16;6(2):e17038. doi: 10.1371/journal.pone.0017038 (PMC3040208; doi:10.1371/journal.pone.0017038)
Supplement: Table S13 — Elements associated with horizontal gene transfer identified in the metagenomes. The insertion sequence family name is given in parantheses where applicable. (PDF) [file pone.0017038.s021.pdf]

Table S13

ID	Indian WWTP Downstream 1	Indian WWTP Downstream 2	Indian WWTP Downstream 3	Indian WWTP Discharge site	Indian WWTP Upstream 1	Indian WWTP Upstream 2	Swedish WWTP Upstream	Swedish WWTP Downstream	Total	Gene/Element
**ARGENE400003**	113	94	47	32	4	3	0	1	294	ISCR2 transposase[a]
**ARGENE100006**	47	21	23	5	4	5	1	0	106	intI1
**ARGENE302403**	0	38	5	15	1	0	0	0	59	ISVsa3 (IS91)[a]
**ARGENE301609**	1	1	2	6	5	2	0	0	17	ISSod9 (Tn3)
**ARGENE301612**	4	2	2	2	2	1	0	1	14	ISSod4 (IS256)
**ARGENE301864**	3	3	0	2	1	1	0	0	10	ISXax1 (IS256)
**ARGENE300568**	2	2	1	2	0	1	1	0	9	ISStma11 (ISL3)
**ARGENE302383**	1	2	0	4	0	0	0	0	7	ISSba16 (IS30)
**ARGENE301407**	0	1	1	0	1	4	0	0	7	ISCARN40 (IS256)
**ARGENE300983**	1	0	2	2	0	2	0	0	7	IS1071 (Tn3)
**ARGENE300971**	0	1	0	3	0	2	0	0	6	ISEcp1 (IS1380)
**ARGENE301785**	0	0	0	1	1	4	0	0	6	IS3000 (Tn3)
**ARGENE100009**	0	0	2	1	0	2	0	0	5	Unspecified integrase
**ARGENE301280**	0	0	0	0	0	0	1	4	5	ISAzo18 (IS3)
**ARGENE301441**	0	1	0	0	0	2	1	1	5	ISArsp6 (Tn3)
**ARGENE300818**	1	1	1	1	0	0	0	0	4	IS6100 (IS6)
**ARGENE300748**	1	1	0	0	0	2	0	0	4	ISEnca1 (IS1380)
**ARGENE302333**	1	0	0	1	0	1	0	1	4	ISSsp5 (IS21)
**ARGENE300310**	2	1	1	0	0	0	0	0	4	IS26 (IS6)
**ARGENE300494**	0	0	0	0	0	1	0	2	3	ISAzvi2 (IS110)
**ARGENE301511**	1	0	0	0	0	0	0	2	3	IS1086 (IS30)
**ARGENE301199**	1	2	0	0	0	0	0	0	3	ISMsi3 (IS3)
**ARGENE301178**	0	2	0	1	0	0	0	0	3	ISMasp7 (IS1380)
**ARGENE300023**	1	0	0	0	1	1	0	0	3	ISThsp5 (IS21)
**ARGENE301897**	1	0	0	1	0	1	0	0	3	IS1247 (IS1380)
**ARGENE300311**	0	0	0	0	1	0	0	2	3	ISMex28 (IS5)
**ARGENE301045**	0	1	0	1	1	0	0	0	3	ISCARN27 (IS256)
**ARGENE302561**	0	0	0	3	0	0	0	0	3	ISPpu12 (ISL3)
**ARGENE301455**	0	0	0	0	0	0	0	3	3	ISAzo28 (IS110)
**ARGENE302158**	3	0	0	0	0	0	0	0	3	ISSse3 (IS3)
**ARGENE301980**	1	0	0	0	0	1	0	1	3	ISCARN70 (IS4)
**ARGENE301675**	0	0	0	2	0	1	0	0	3	IS1419 (IS481)
**ARGENE301721**	0	0	0	0	0	1	1	0	2	ISPps1 (IS91)
**ARGENE300954**	0	0	0	0	0	0	1	1	2	ISXau2 (IS3)
**ARGENE302116**	1	0	0	0	0	0	1	0	2	ISPpu7 (IS21)
**ARGENE300390**	0	0	0	1	1	0	0	0	2	IS881 (IS5)
**ARGENE300394**	0	0	0	1	0	1	0	0	2	IS5 (IS5)
**ARGENE301282**	0	0	0	0	0	0	1	1	2	ISAzo10 (IS3)
**ARGENE301625**	0	0	1	0	0	0	0	1	2	ISMch1 (IS3)

**ARGENE302128**	1	0	0	0	0	1	0	0	2	ISXac3 (IS3)
**ARGENE301344**	0	0	0	1	1	0	0	0	2	ISCte3 (IS3)
**ARGENE302286**	0	0	0	0	0	0	0	2	2	ISAzo9 (IS630)
**ARGENE301639**	0	0	1	0	0	0	0	1	2	ISBmu3 (IS21)
**ARGENE301084**	0	0	0	0	0	0	1	1	2	ISMdi21 (IS5)
**ARGENE300823**	0	0	0	1	0	1	0	0	2	ISKpn10 (IS3)
**ARGENE302507**	0	0	0	0	0	1	0	1	2	ISPosp7 (IS1380)
**ARGENE300981**	0	1	0	0	0	0	0	1	2	ISDar2 (IS4)
**ARGENE300036**	0	0	1	0	0	1	0	0	2	ISIlo12 (IS3)
**ARGENE301619**	1	0	0	0	1	0	0	0	2	ISEca11 (IS3)
**ARGENE301767**	0	0	0	0	1	0	0	1	2	ISSpma1 (IS3)
**ARGENE302515**	0	0	0	0	0	1	0	1	2	ISNeu4 (IS3)
**ARGENE300020**	1	0	0	0	0	1	0	0	2	ISThsp2 (IS21)
**ARGENE300599**	0	0	0	1	0	0	0	1	2	ISPre1 (IS5)
**ARGENE300597**	0	0	0	0	0	0	0	2	2	ISPre3 (IS66)
**ARGENE300689**	2	0	0	0	0	0	0	0	2	IS1541C (IS200/IS605)
**ARGENE301086**	0	0	0	0	1	1	0	0	2	ISMdi25 (IS30)
**ARGENE300716**	1	0	0	0	0	0	0	1	2	ISGdi8 (IS256)
**ARGENE302327**	0	0	1	0	0	0	0	1	2	ISPpu22 (IS3)
**ARGENE300161**	0	0	0	2	0	0	0	0	2	ISAde4 (IS3)
**ARGENE301965**	1	0	0	0	0	1	0	0	2	ISPme1 (IS1380)
**ARGENE302201**	0	0	0	0	0	1	0	1	2	ISApr6 (IS66)
**ARGENE302334**	0	0	1	0	0	0	0	1	2	ISSsp2 (IS3)
**ARGENE302332**	1	0	1	0	0	0	0	0	2	ISSsp4 (IS21)
**ARGENE302537**	1	0	0	0	0	1	0	0	2	ISEli1 (IS3)
**ARGENE300777**	0	0	0	0	0	0	1	1	2	ISPst3 (IS21)
**ARGENE301956**	1	0	0	0	0	1	0	0	2	ISPa29 (IS110)
**ARGENE300797**	0	0	0	0	0	2	0	0	2	ISUnCu5 (IS4)
**ARGENE300795**	0	0	0	0	1	1	0	0	2	ISUnCu3 (IS21)
**ARGENE301048**	0	0	0	0	0	0	0	2	2	ISCARN22 (IS66)
**ARGENE301122**	0	1	0	0	0	1	0	0	2	ISDde1 (IS5)
**ARGENE300947**	0	0	0	0	2	0	0	0	2	ISAzsp1 (IS3)
**ARGENE300551**	0	0	0	2	0	0	0	0	2	ISGdi17 (IS21)
**ARGENE300883**	0	1	0	0	0	0	0	1	2	ISCARN10 (IS200/IS605)
**ARGENE302141**	0	0	0	1	0	0	0	1	2	ISRso16 (IS3)
**ARGENE302143**	0	0	0	0	0	0	0	2	2	ISRso11 (IS3)
**ARGENE302562**	1	0	0	0	0	0	0	1	2	ISPpu19 (IS66)
**ARGENE302028**	0	0	0	0	0	1	1	0	2	ISCARN97 (IS110)
**ARGENE301914**	0	0	0	0	0	2	0	0	2	IS53 (IS21)
**ARGENE301714**	0	0	0	0	0	0	0	2	2	ISXo7 (IS630)
**ARGENE301717**	0	0	0	0	2	0	0	0	2	ISPpa2 (IS5)
**ARGENE301712**	0	0	0	1	1	0	0	0	2	ISPpa5 (IS66)

**ARGENE301927**	1	1	0	0	0	0	0	0	2	ISRme4 (IS21)
**ARGENE301279**	0	0	0	1	0	1	0	0	2	IS4351 (IS30)
**ARGENE301493**	0	0	1	0	0	1	0	0	2	ISMex39 (IS21)
**ARGENE301933**	0	1	0	0	0	1	0	0	2	ISBthe1 (IS4)
**ARGENE300289**	0	1	0	0	0	0	1	0	2	ISAZo16 (IS21)
**ARGENE300411**	1	0	0	1	0	0	0	0	2	ISMca3 (IS3)
**ARGENE301449**	1	0	0	0	1	0	0	0	2	ISAzo26 (IS91)
**ARGENE302438**	1	0	0	0	1	0	0	0	2	ISVsp19 (IS1380)
**ARGENE300253**	0	0	1	1	0	0	0	0	2	ISRme10 (IS30)
**ARGENE300671**	0	0	0	0	0	0	0	2	2	ISNieu3 (IS3)
**ARGENE100008**	0	0	0	1	0	0	0	0	1	intI2
**ARGENE300920**	0	0	0	0	0	1	0	0	1	ISDha10 (IS110)
**ARGENE302426**	0	0	0	0	0	1	0	0	1	ISAli4 (IS3)
**ARGENE302421**	0	1	0	0	0	0	0	0	1	ISAli2 (IS630)
**ARGENE300245**	1	0	0	0	0	0	0	0	1	IS1491 (IS21)
**ARGENE300391**	0	1	0	0	0	0	0	0	1	IS883 (IS66)
**ARGENE300669**	0	0	0	0	0	0	0	1	1	ISThsp19 (IS21)
**ARGENE300663**	0	0	0	0	0	1	0	0	1	ISThsp10 (IS21)
**ARGENE301281**	0	1	0	0	0	0	0	0	1	ISAzo19 (IS66)
**ARGENE301287**	0	0	0	1	0	0	0	0	1	ISAzo15 (IS66)
**ARGENE301285**	0	0	0	0	0	0	0	1	1	ISGur11 (IS481)
**ARGENE301624**	0	0	0	0	0	1	0	0	1	ISAcma31 (IS630)
**ARGENE301754**	0	0	0	0	0	1	0	0	1	ISPa32 (IS3)
**ARGENE301759**	1	0	0	0	0	0	0	0	1	ISNisp1 (IS3)
**ARGENE300933**	0	1	0	0	0	0	0	0	1	ISDha13 (IS200/IS605)
**ARGENE300506**	0	0	0	1	0	0	0	0	1	ISCce1 (IS481)
**ARGENE300501**	0	0	0	1	0	0	0	0	1	ISSpwi1 (IS3)
**ARGENE302412**	0	0	0	0	1	0	0	0	1	ISTesp1 (IS3)
**ARGENE301900**	0	0	0	0	0	1	0	0	1	ISCausp1 (IS3)
**ARGENE301958**	1	0	0	0	0	0	0	0	1	ISPa27 (IS256)
**ARGENE302287**	0	1	0	0	0	0	0	0	1	IS53K (IS21)
**ARGENE302284**	0	0	0	0	1	0	0	0	1	ISAzo5 (IS4)
**ARGENE300015**	0	0	0	0	1	0	0	0	1	ISThsp9 (Tn3)
**ARGENE300651**	0	0	0	1	0	0	0	0	1	ISMex22 (Tn3)
**ARGENE301749**	0	0	0	0	0	0	0	1	1	ISStma5 (IS3)
**ARGENE301406**	0	0	0	0	0	0	0	1	1	ISCARN47 (IS256)
**ARGENE302248**	0	0	0	1	0	0	0	0	1	ISAs2 (IS30)
**ARGENE302241**	0	0	0	0	0	0	0	1	1	ISAzo31 (IS5)
**ARGENE301644**	1	0	0	0	0	0	0	0	1	ISAzo34 (IS256)
**ARGENE300649**	0	0	0	1	0	0	0	0	1	ISSfr1 (IS110)
**ARGENE301510**	0	0	0	0	1	0	0	0	1	IS1087 (IS3)
**ARGENE301353**	0	0	0	0	0	1	0	0	1	ISMsm12 (IS1380)

**ARGENE300734**	1	0	0	0	0	0	0	0	1	ISCsp1 (IS256)
**ARGENE300917**	0	0	0	0	1	0	0	0	1	ISMdi13 (IS256)
**ARGENE300912**	0	1	0	0	0	0	0	0	1	ISWsu1 (IS3)
**ARGENE301994**	0	0	0	1	0	0	0	0	1	ISRta2 (IS256)
**ARGENE301491**	0	0	0	0	1	0	0	0	1	ISYps3 (Tn3)
**ARGENE302273**	0	0	1	0	0	0	0	0	1	IS1353 (IS3)
**ARGENE302277**	0	0	0	1	0	0	0	0	1	IS1356 (IS256)
**ARGENE302275**	0	0	0	0	1	0	0	0	1	IS1355 (IS5)
**ARGENE302505**	1	0	0	0	0	0	0	0	1	ISPosp5 (IS3)
**ARGENE302506**	0	0	0	0	0	0	0	1	1	ISPosp4 (IS4)
**ARGENE300354**	0	0	0	0	1	0	0	0	1	ISRsp3 (IS21)
**ARGENE300586**	0	0	0	1	0	0	0	0	1	IS1236 (IS3)
**ARGENE300369**	0	0	0	0	0	0	0	1	1	ISDsp1 (IS3)
**ARGENE301410**	0	0	0	0	0	1	0	0	1	ISAzca2 (IS66)
**ARGENE301984**	0	0	0	1	0	0	0	0	1	ISCARN78 (IS21)
**ARGENE301509**	0	1	0	0	0	0	0	0	1	ISJsp2 (IS5)
**ARGENE301504**	0	1	0	0	0	0	0	0	1	ISEnfa200 (IS200/IS605)
**ARGENE302088**	0	0	0	0	1	0	0	0	1	ISRm13 (IS5)
**ARGENE302264**	0	0	0	1	0	0	0	0	1	IS693 (IS66)
**ARGENE302263**	0	0	1	0	0	0	0	0	1	ISMaq1 (IS3)
**ARGENE300838**	0	0	0	0	0	0	0	1	1	ISPa8 (IS5)
**ARGENE302516**	0	0	0	0	0	0	0	1	1	ISNeu2 (IS982)
**ARGENE300542**	0	0	0	1	0	0	0	0	1	ISAac2 (IS5)
**ARGENE300545**	0	0	0	0	0	1	0	0	1	IS1051 (IS5)
**ARGENE300596**	0	0	0	0	1	0	0	0	1	ISPre4 (IS21)
**ARGENE300372**	0	0	0	0	0	0	0	1	1	ISDsp2 (IS3)
**ARGENE300684**	0	0	0	0	1	0	0	0	1	ISSso1 (IS110)
**ARGENE301376**	0	0	0	1	0	0	0	0	1	ISNmo3 (IS481)
**ARGENE301404**	0	0	0	0	0	0	0	1	1	ISCARN45 (IS256)
**ARGENE301401**	0	0	0	0	0	1	0	0	1	ISCARN48 (IS66)
**ARGENE301403**	0	0	0	0	0	0	0	1	1	ISCARN44 (IS630)
**ARGENE302071**	0	0	0	1	0	0	0	0	1	ISMex7 (IS3)
**ARGENE302078**	0	0	0	0	1	0	0	0	1	ISXca1 (IS3)
**ARGENE301883**	0	1	0	0	0	0	0	0	1	ISMex14 (IS256)
**ARGENE301885**	0	0	0	0	0	1	0	0	1	ISMex12 (IS110)
**ARGENE301888**	0	0	1	0	0	0	0	0	1	ISMex11 (IS3)
**ARGENE302326**	0	0	0	0	0	1	0	0	1	ISPpu21 (IS5)
**ARGENE302452**	1	0	0	0	0	0	0	0	1	IS1383 (IS110)
**ARGENE302450**	0	0	0	0	0	1	0	0	1	IS1384 (IS5)
**ARGENE300564**	0	0	0	1	0	0	0	0	1	ISRde2 (IS3)
**ARGENE300567**	0	0	0	0	1	0	0	0	1	ISStma10 (IS630)
**ARGENE300163**	0	0	0	0	0	0	0	1	1	ISAde3 (IS3)

**ARGENE300762**	0	1	0	0	0	0	0	0	1	ISGNB1-1 (IS5)
**ARGENE300784**	0	0	0	1	0	0	0	0	1	IS1133 (IS3)
**ARGENE300785**	0	0	1	0	0	0	0	0	1	IS1132 (IS256)
**ARGENE302065**	1	0	0	0	0	0	0	0	1	ISAbo1 (IS3)
**ARGENE302068**	0	0	0	0	0	0	1	0	1	ISPlu2 (IS200/IS605)
**ARGENE301896**	0	0	0	1	0	0	0	0	1	IS1240 (IS3)
**ARGENE301898**	0	1	0	0	0	0	0	0	1	IS1246 (IS5)
**ARGENE301789**	0	0	0	0	0	0	1	0	1	ISYen2B (IS21)
**ARGENE302193**	0	0	0	0	0	1	0	0	1	ISApr9 (IS1380)
**ARGENE302195**	0	0	0	0	0	1	0	0	1	ISApr8 (IS1380)
**ARGENE300577**	0	0	0	0	0	1	0	0	1	ISAs1 (ISAs1)
**ARGENE302293**	0	0	0	1	0	0	0	0	1	ISRasp1 (IS3)
**ARGENE300312**	0	0	0	0	0	1	0	0	1	ISVme1 (IS66)
**ARGENE301318**	0	0	0	0	0	1	0	0	1	IS1474 (IS21)
**ARGENE300779**	0	0	0	0	1	0	0	0	1	ISPst5 (IS5)
**ARGENE300950**	0	0	0	0	0	1	0	0	1	ISBusp2 (IS5)
**ARGENE301227**	0	0	0	1	0	0	0	0	1	ISSusp2 (IS3)
**ARGENE301553**	1	0	0	0	0	0	0	0	1	ISGme3 (IS5)
**ARGENE302052**	0	0	0	0	1	0	0	0	1	ISPsy17 (IS256)
**ARGENE302051**	0	0	0	1	0	0	0	0	1	ISAcma13 (IS3)
**ARGENE301049**	0	0	0	0	0	0	0	1	1	ISCARN20 (IS110)
**ARGENE302239**	0	0	0	0	0	1	0	0	1	ISThi1 (IS5)
**ARGENE302231**	1	0	0	0	0	0	0	0	1	ISPsy24 (IS3)
**ARGENE302540**	0	1	0	0	0	0	0	0	1	ISCARN114 (IS30)
**ARGENE300094**	0	0	0	0	0	1	0	0	1	IS1395 (IS256)
**ARGENE300097**	1	0	0	0	0	0	0	0	1	IS1396 (ISL3)
**ARGENE300548**	1	0	0	0	0	0	0	0	1	ISGdi10 (IS481)
**ARGENE300436**	0	0	0	0	0	1	0	0	1	ISSpu8 (IS630)
**ARGENE300327**	0	0	0	0	0	0	1	0	1	ISOba3 (IS1)
**ARGENE301051**	0	0	0	0	0	0	1	0	1	IS30D (IS30)
**ARGENE300949**	0	0	0	1	0	0	0	0	1	ISBusp1 (Tn3)
**ARGENE300414**	0	0	1	0	0	0	0	0	1	ISMca4 (IS3)
**ARGENE300417**	0	0	1	0	0	0	0	0	1	ISAzo41 (IS5)
**ARGENE302040**	0	0	0	0	0	1	0	0	1	ISHne4 (IS110)
**ARGENE302229**	0	0	1	0	0	0	0	0	1	ISPsy20 (IS21)
**ARGENE302225**	0	1	0	0	0	0	0	0	1	ISPsy28 (IS3)
**ARGENE301836**	1	0	0	0	0	0	0	0	1	ISSpo4 (IS5)
**ARGENE300159**	0	0	0	0	0	1	0	0	1	ISXo17 (IS3)
**ARGENE301332**	1	0	0	0	0	0	0	0	1	IS1193D (ISL3)
**ARGENE301330**	0	0	0	0	1	0	0	0	1	ISMex29 (IS110)
**ARGENE300552**	1	0	0	0	0	0	0	0	1	ISGdi14 (IS3)
**ARGENE301680**	0	0	0	1	0	0	0	0	1	ISLxc1 (IS21)

**ARGENE301201**	0	0	0	0	0	0	0	1	1	ISMsi5 (IS3)
**ARGENE300337**	0	0	0	0	1	0	0	0	1	ISBj4 (IS110)
**ARGENE301069**	1	0	0	0	0	0	0	0	1	ISPa16 (IS5)
**ARGENE301844**	0	0	0	0	0	1	0	0	1	ISAav1 (IS21)
**ARGENE301849**	0	0	0	0	0	0	0	1	1	ISCARN80 (IS110)
**ARGENE301448**	0	0	0	0	0	0	1	0	1	ISAzo27 (IS5)
**ARGENE302360**	0	0	0	0	0	0	0	1	1	ISBcen3 (IS110)
**ARGENE300439**	0	1	0	0	0	0	0	0	1	ISHar4 (IS30)
**ARGENE301825**	1	0	0	0	0	0	0	0	1	ISGsu1 (IS4)
**ARGENE301821**	0	0	0	1	0	0	0	0	1	ISRusp1 (IS5)
**ARGENE300639**	0	0	0	0	0	0	0	1	1	ISMrsp5 (IS66)
**ARGENE301320**	0	0	0	1	0	0	0	0	1	ISMex27 (IS21)
**ARGENE300527**	0	0	0	0	0	0	1	0	1	ISMsm2 (IS630)
**ARGENE301692**	0	0	0	1	0	0	0	0	1	IS1326 (IS21)
**ARGENE301852**	0	0	0	0	0	1	0	0	1	IS66-1 (IS66)
**ARGENE301613**	0	0	0	1	0	0	0	0	1	ISSod5 (IS256)
**ARGENE301451**	0	0	0	0	0	1	0	0	1	ISAzo20 (IS200/IS605)
**ARGENE301102**	0	0	0	0	1	0	0	0	1	ISGme2 (IS4)
**ARGENE300743**	0	0	0	0	0	1	0	0	1	IS640 (IS21)
**ARGENE300744**	0	0	0	1	0	0	0	0	1	IS1021 (IS5)
**ARGENE301133**	0	0	0	0	1	0	0	0	1	IS1F (IS1)
**ARGENE301861**	0	0	0	0	0	1	0	0	1	IS1403 (IS3)
**ARGENE300195**	0	0	0	0	0	0	1	0	1	ISRso9 (IS5)
**ARGENE300198**	0	0	0	0	1	0	0	0	1	ISRso5 (IS630)
**ARGENE300440**	0	0	0	0	0	0	0	1	1	ISHar7 (IS3)
**ARGENE301912**	0	0	0	0	1	0	0	0	1	ISVch3 (IS21)
**ARGENE301668**	0	0	0	1	0	0	0	0	1	IS1413 (IS256)
**ARGENE301669**	1	0	0	0	0	0	0	0	1	IS1411 (ISL3)
**ARGENE301267**	0	0	0	1	0	0	0	0	1	ISGur15 (IS21)
**ARGENE301829**	0	0	0	0	0	0	1	0	1	ISGsu4 (IS110)
**ARGENE300846**	0	0	0	0	0	1	0	0	1	ISRm7 (IS3)
**ARGENE300999**	0	0	0	0	1	0	0	0	1	IS222 (IS3)
**ARGENE302161**	0	0	0	0	0	1	0	0	1	ISPepr5 (IS21)
**ARGENE301713**	0	0	0	0	0	0	0	1	1	ISPpa4 (IS5)
**ARGENE302347**	0	0	0	0	1	0	0	0	1	ISCARN33 (IS5)
**ARGENE302342**	0	1	0	0	0	0	0	0	1	ISAau3 (IS5)
**ARGENE301146**	0	0	0	1	0	0	0	0	1	IS17 (IS5)
**ARGENE301926**	0	0	0	0	1	0	0	0	1	ISRme1 (IS5)
**ARGENE301347**	1	0	0	0	0	0	0	0	1	IS-LL6 (IS3)
**ARGENE300610**	1	0	0	0	0	0	0	0	1	ISAba2 (IS3)
**ARGENE300616**	0	0	0	0	0	0	0	1	1	ISXc5 (Tn3)
**ARGENE300508**	1	0	0	0	0	0	0	0	1	ISThsp21 (IS5)

**ARGENE301676**	0	0	0	1	0	0	0	0	1	ISSau1 (IS30)
**ARGENE301273**	0	0	0	0	0	1	0	0	1	ISAca1 (IS3)
**ARGENE300871**	1	0	0	0	0	0	0	0	1	ISBcen17 (IS3)
**ARGENE300876**	0	0	0	0	1	0	0	0	1	ISBcen18 (IS256)
**ARGENE300875**	0	0	0	0	0	0	1	0	1	ISBcen19 (IS66)
**ARGENE301474**	1	0	0	0	0	0	0	0	1	ISNmu3 (IS3)
**ARGENE300478**	1	0	0	0	0	0	0	0	1	ISAtu3 (IS3)
**ARGENE301708**	0	0	0	1	0	0	0	0	1	ISPca1 (IS4)
**ARGENE301492**	0	0	0	0	0	0	0	1	1	IS1311 (IS256)
**ARGENE302007**	0	0	0	0	0	1	0	0	1	IS1X1 (IS1)
**ARGENE301202**	0	0	0	0	0	1	0	0	1	ISMsi4 (IS3)
**ARGENE301200**	0	0	1	0	0	0	0	0	1	ISMsi2 (IS3)
**ARGENE301497**	0	0	0	1	0	0	0	0	1	ISSce1 (IS1)
**ARGENE300334**	0	0	0	0	0	1	0	0	1	ISR1 (IS3)
**ARGENE302167**	0	0	0	0	0	1	0	0	1	ISRssp8 (IS1380)
**ARGENE301220**	1	0	0	0	0	0	0	0	1	ISHha1 (IS3)
**ARGENE301638**	0	0	0	0	0	0	0	1	1	ISBmu2 (IS5)
**ARGENE301935**	0	0	0	0	0	1	0	0	1	ISCph2 (IS4)
**ARGENE302337**	0	0	0	0	0	0	1	0	1	ISMca2 (IS3)
**ARGENE300515**	0	0	0	0	1	0	0	0	1	ISBxe3 (IS481)
**ARGENE300609**	0	0	0	1	0	0	0	0	1	ISAba1 (IS4)
**ARGENE300601**	0	0	0	0	0	1	0	0	1	ISGur4 (IS4)
**ARGENE300603**	0	0	0	1	0	0	0	0	1	ISGur6 (IS3)
**ARGENE300602**	0	0	0	0	0	0	1	0	1	ISCod1 (IS3)
**ARGENE300216**	0	0	0	0	0	0	1	0	1	ISCARN6 (IS200/IS605)
**ARGENE301735**	0	0	0	1	0	0	0	0	1	ISRosp2 (IS3)
**ARGENE300346**	1	0	0	0	0	0	0	0	1	ISPen2 (IS3)
**ARGENE302299**	0	0	0	0	0	1	0	0	1	ISRso20 (IS3)
**ARGENE302100**	0	0	0	0	1	0	0	0	1	ISAnsp11 (IS3)
**ARGENE300258**	0	0	0	0	1	0	0	0	1	ISRme16 (IS5)
**ARGENE301485**	0	0	0	0	0	0	0	1	1	ISMex31 (IS110)
**ARGENE300257**	1	0	0	0	0	0	0	0	1	ISRme15 (IS3)
**ARGENE302434**	0	0	0	1	0	0	0	0	1	ISRpa2 (IS3)
**ARGENE300071**	0	0	1	0	0	0	0	0	1	ISPsp3 (IS5)
**ARGENE300076**	0	0	0	0	1	0	0	0	1	ISBlma7 (ISAs1)
**ARGENE300704**	0	0	0	0	0	1	0	0	1	ISMt1 (IS5)
**ARGENE300252**	0	0	0	1	0	0	0	0	1	IS50R (IS4)
**ARGENE300708**	0	0	0	0	0	1	0	0	1	ISApu1 (IS4)
**ARGENE300670**	0	0	1	0	0	0	0	0	1	ISNieu2 (IS3)

[a]It is known that the ISVsa3 insertion sequence contain an ISCR2 transposase and ARGENE400003 and ARGENE302403 may therefore correspond to the same mobile elements. Note, however, that the ISCR2 transposase (ARGENE400003) is detected based on the presence of the peptide sequence of the transposase and is therefore likely to represent other configurations than the ISVsa3 insertion sequence.
