# Supplementary material for: Pyrosequencing of Antibiotic-Contaminated River Sediments Reveals High Levels of Resistance and Gene Transfer Elements
Source: PLoS One. 2011 Feb 16;6(2):e17038. doi: 10.1371/journal.pone.0017038 (PMC3040208; doi:10.1371/journal.pone.0017038)
Supplement: Table S14 — Resistance genes and mechanisms of horizontal gene transfer with a significantly different relative abundance between the Indian up and downstream metagenomes. (PDF) [file pone.0017038.s022.pdf]

Table S14

GeneFamily	Coefficient	AIC	P-value	FDR	Annotation
**ARGENE000142**	4.19	40.97	0	0	*sul2*
**ARGENE000144**	3.08	36.56	5.36E-59	7.95E-56	aph6id (*strA*)
**ARGENE000034**	3.99	34.12	5.88E-50	5.81E-47	aph33ib (*strB*)
**ARGENE400003**	3.18	50.57	8.65E-47	6.42E-44	ISCR2 transposase
**ARGENE200007**	-3.69	67.63	1.07E-19	6.36E-17	*qnrD*
**ARGENE100006**	1.90	26.95	3.35E-12	1.58E-09	*intI1*
**ARGENE302403**	3.35	107.73	2.01E-09	7.47E-07	ISVsa3 (IS91)
**ARGENE200006**	-3.46	17.41	1.93E-08	5.73E-06	*qnrC*
**ARGENE200008**	-22.11	13.20	9.91E-07	0.000267	*qnrS*
**ARGENE000107**	-21.03	16.08	2.58E-06	0.00059	bl2_ges (*blaGES*)
**ARGENE000244**	-24.63	10.58	0.000124	0.02621	ant3ia (*aadA*)
