# Supplementary material for: Pyrosequencing of Antibiotic-Contaminated River Sediments Reveals High Levels of Resistance and Gene Transfer Elements
Source: PLoS One. 2011 Feb 16;6(2):e17038. doi: 10.1371/journal.pone.0017038 (PMC3040208; doi:10.1371/journal.pone.0017038)
Supplement: Table S15 — Resistance genes and mechanisms of horizontal gene transfer with a significantly different relative abundance between the Indian upstream and Swedish metagenomes. (PDF) [file pone.0017038.s023.pdf]

Table S15

GeneFamily	Coefficient	AIC	P-value	FDR	Annotation
**ARGENE200007**	20.19	62.24	4.29E-15	1.27E-11	*qnrD*
**ARGENE200006**	24.27	12.57	7.86E-07	0.000583	*qnrC*
**ARGENE000142**	22.27	14.05	7.86E-07	0.000583	*sul2*
**ARGENE200008**	21.79	13.20	0.000102	0.060504	*qnrS*
**ARGENE000107**	20.71	16.08	0.000189	0.080063	bl2_ges (*blaGES*)
