# Supplementary material for: Pyrosequencing of Antibiotic-Contaminated River Sediments Reveals High Levels of Resistance and Gene Transfer Elements
Source: PLoS One. 2011 Feb 16;6(2):e17038. doi: 10.1371/journal.pone.0017038 (PMC3040208; doi:10.1371/journal.pone.0017038)
Supplement: Table S16 — Resistance genes and mechanisms of horizontal gene transfer with a significantly different relative abundance between the Indian downstream and Swedish metagenomes. (PDF) [file pone.0017038.s024.pdf]

Table S16

GeneFamily	Coefficient	AIC	P-value	FDR	Annotation
**ARGENE000142**	26.46	30.91	0	0	*sul2*
**ARGENE000144**	23.61	26.82	5.93E-62	8.80E-59	aph6id (*strB*)
**ARGENE000034**	23.31	26.08	2.07E-46	2.04E-43	aph33ib (*strA*)
**ARGENE400003**	4.89	49.11	7.44E-46	5.52E-43	ISCR2 transposase
**ARGENE100006**	3.86	22.21	2.20E-16	1.30E-13	*intI1*
**ARGENE302403**	18.57	104.61	1.92E-09	8.12E-07	ISVsa3 (IS91)
